# Targeted Regulation of HSP70 by the ARP2/3 Complex in Mammary Epithelial Cells and Its Impact on Host Cell Apoptosis

**DOI:** 10.3390/biom15040538

**Published:** 2025-04-07

**Authors:** Tingji Yang, Bo Fang, Yan Chen, Dan Bao, Jiang Zhang, Peiwen Liu, Zhiwei Duan, Yuxuan He, Xingxu Zhao, Quan-Wei Zhang, Wei-Tao Dong, Yong Zhang

**Affiliations:** 1 College of Veterinary Medicine, Gansu Agricultural University, 1#Ying Men-Cun Road, Lanzhou 730070, China; 107332202109@st.gsau.edu.cn (T.Y.); 107332202103@st.gsau.edu.cn (B.F.); 107332101041@st.gsau.edu.cn (Y.C.); 1073323020133@st.gsau.edu.cn (D.B.); 1073323120273@st.gsau.edu.cn (J.Z.); 1073324140048@st.gsau.edu.cn (P.L.); heyx@gsau.edu.cn (Y.H.); zhaoxx@gsau.edu.cn (X.Z.); zhangyong@gsau.edu.cn (Y.Z.); 2 Gansu Key Laboratory of Animal Generational Physiology and Reproductive Regulation, Lanzhou 730070, China; zhangqw@gsau.edu.cn; 3 College of Life Sciences and Biotechnology, Gansu Agricultural University, Lanzhou 730030, China

**Keywords:** mastitis, ARP2/3 complex, ARPC3/ARPC4, HSP70, apoptosis

## Abstract

Mastitis is frequently triggered by the bacterial disruption of the epithelial cell barrier. The actin-related protein 2/3 complex (Arp2/3), a major endogenous protein involved in cytoskeletal regulation, plays a crucial role in preserving epithelial barrier integrity during inflammation; however, its specific role in mastitis progression remains unclear. This study aims to use lipopolysaccharide (LPS) to establish mammary alveolar cells-large T antigen cells (MAC-T is a bovine mammary epithelial cell line) and mouse models of mastitis, investigating the functional relationship between actin-related protein 2/3 complex subunits 3 (ARPC3) and 4 (ARPC4) and heat shock protein 70 (HSP70) during mammary epithelial cell inflammation and assessing its effects on apoptosis. Transcriptomic sequencing initially identified 48 differentially expressed genes associated with the bacterial invasion of epithelial cells and apoptosis. Further molecular biology analyses showed a significant upregulation of ARPC3/ARPC4 and HSP70 expression during inflammation, along with a marked increase in apoptosis rates. When ARPC3/ARPC4 was inhibited using CK666, HSP70 expression further increased compared to the LPS group, while inflammatory factors, apoptosis rates, and apoptosis-related protein expression were notably reduced. These findings indicate that targeting ARPC3/ARPC4 to regulate HSP70 can promote inflammation and apoptosis, highlighting its potential as a therapeutic target for mastitis.

## 1. Introduction

Bovine mastitis is primarily caused by pathogenic bacterial invasion, with *Escherichia coli* (*E.coli*) being the main environmental pathogen responsible for this condition [1]. The key virulence factor lipopolysaccharide (LPS) facilitates bacterial colonization within host cells by promoting actin assembly to enhance invasive efficiency, concomitantly inducing inflammation and apoptosis [2]. During the invasion of bovine mammary epithelial cells, LPS activates the Toll-like receptor 4(TLR4)-mediated signaling pathway, inducing cytoskeletal rearrangement and phosphorylation cascades that facilitate bacterial entry [3,4]. The actin-related protein 2/3 complex (Arp2/3 complex) plays a crucial role in bacterial invasion. Within host cells, bacterial motility is achieved by surface virulence factors binding to the Wiskott–Aldrich Syndrome protein (WASP) family, which activates the Arp2/3 complex to initiate actin nucleation, leading to re-polymerization and new microfilament formation [5]. Studies have shown that both enteropathogenic *E.coli* (EPEC) and enterohemorrhagic *E.coli* (EHEC) utilize this mechanism to invade host cells [6,7]. Tight junctions are disrupted when LPS invades the epithelial cell layer. Once inside, LPS stimulates macrophages to secrete inflammatory cytokines. The accumulation of these cytokines leads to epithelial cell apoptosis, thereby impairing the epithelial barrier function [8,9,10]. Moreover, actin cytoskeleton changes can disrupt tight junctions, further compromising the epithelial barrier [11]. The Arp2/3 complex is a critical activator of actin cytoskeleton rearrangement [12]. Therefore, abnormal Arp2/3 activity can disrupt tight junctions, leading to impaired epithelial barrier function. ARPC3/ARPC4 regulate the nucleation activity of the Arp2/3 complex and influence tight junction assembly, thereby maintaining the integrity of the epithelial barrier.

The Arp2/3 complex consists of seven subunits, among which actin-related protein 2/3 complex subunits 3 (ARPC3) and 4 (ARPC4) are especially important. ARPC3 acts as an intermediary for signal transduction between WASP and the Arp2/3 complex; without ARPC3, the nucleation activity of the Arp2/3 complex is reduced [13]. ARPC3 also plays a significant role in maintaining normal epidermal function in mice and in the intracellular motility of *Listeria monocytogenes* [14,15]. The surface of ARPC4 contains residues essential for the Arp2/3 complex’s biological activity, particularly for F-actin binding; the affinity of these residues for F-actin influences Arp2/3 complex activation [16]. Research has shown that knocking down ARPC4 in Human Bladder Transitional Cell Carcinoma (T24 cells) inhibits pseudopod formation, and downregulating ARPC4 suppresses filamentous actin cytoskeleton synthesis [17]. Additionally, during bacterial toxin penetration, the upregulation of ARPC3/ARPC4 accelerates Arp2/3-mediated actin cytoskeletal rearrangement, promoting disease progression [18,19]. The Arp2/3 complex is also involved in apoptosis pathways. The activation of the Arp2/3 complex enhances actin polymerization, increasing apoptosis rates [20]; membrane blebbing, cell contraction, and chromatin condensation during apoptosis rely on dynamic changes in microfilament assembly and disassembly mediated by ARPC3/ARPC4 [21]. However, the specific mechanism by which the Arp2/3 complex regulates apoptosis factor expression remains unknown.

A key pathway regulating host cell apoptosis involves the heat shock protein family, which alleviates endoplasmic reticulum stress by repairing misfolded proteins. The heat shock protein family is highly conserved and upregulated under stress conditions, such as high temperatures and bacterial infections. Within this family, heat shock protein 70 (HSP70) expression is closely linked to disease severity, as its upregulation mitigates protein homeostasis disruption and apoptosis induced by external stressors, thereby maintaining cell function and protecting the organism [22]. In various diseases, HSP70 modulates nuclear transcription factor-Κb (NF-κB) pathway-mediated inflammatory responses and is involved in apoptosis regulation. Extensive studies have shown that HSP70 interferes with the LPS activation of the NF-κB pathway, impacting cellular immune responses [23,24]. The HSP70-TLR4 signaling pathway has also been shown to reduce mitochondrial damage and inhibit excessive reactive oxygen species (ROS) production within cells, thereby suppressing apoptosis [25,26]. In human histiocytic lymphoma cell (U-937 cell) apoptosis, HSP70 significantly inhibits Caspase3 and Caspase7 expression, interrupting the Caspase family cascade and reducing apoptosis rates [27].

Our previous research established an lipoteichoic acid (LTA)-induced mammary alveolar cells-large T antigen (MAC-T) cell inflammation model, revealing through multi-omics analyses that ARPC3/ARPC4 and HSP70 expressions were upregulated, with both involved in bacterial invasion and endocytosis pathways in epithelial cells. These findings were further validated in cellular and animal models, where multiple apoptosis factors were also found to be upregulated [28,29]. Based on these findings, we hypothesize that the Arp2/3 complex and HSP70 both participate in apoptosis pathways induced by inflammatory responses and may interact. This study aims to explore, using molecular biology techniques, whether a potential functional relationship exists between ARPC3/ARPC4 and HSP70 in LPS-induced mastitis and to elucidate the roles of ARPC3/ARPC4 and HSP70 in MAC-T cell apoptosis, providing insights into new therapeutic targets for mastitis.

## 2. Materials and Methods

### 2.1. Tissue Sample Preparation

SPF-grade Kunming mice (7–8 weeks old; 30–35 g; 12 females and 4 males) were purchased from the Lanzhou Veterinary Research Institute of the Chinese Academy of Agricultural Sciences [28,29]. The mice were housed in a sterile environment at 22–25 °C and allowed to acclimate for one week. They were housed in cages containing three females and one male each. After pregnancy was confirmed, the female mice were randomly assigned to either the control or experimental group, with six mice per group. The fourth pair of mammary glands in each mouse was injected with the following solutions: 50 μL of saline for the control group and 50 μL of LPS at a concentration of 0.2 mg/mL for the experimental group [30]. After 24 h post-injection, the mice were euthanized. Mammary tissue samples were collected, with some stored at −80 °C and others fixed in 4% paraformaldehyde. Animal experiments were approved by the Animal Protection Committee of Gansu Agricultural University (Lanzhou, China, approval No.: GSAC-Eth-VMC-2024-031).

### 2.2. Cell Culture and Treatment

The bovine mammary epithelial cell line (MAC-T) was kindly provided by Professor Jianxi Li from the Lanzhou Institute of Animal Husbandry and Veterinary Medicine. The cells were cultured in Gibco RPMI-1640 medium (Gibco, New York, NY, USA) supplemented with 10% fetal bovine serum (Invitrogen, Irvine, CA, USA) at 37 °C in a 5% CO_2_ incubator. The cells were grown until they reached 80% confluence. For treatment, LPS (Sigma, St. Louis, MO, USA) was used at concentrations of 1, 10, 20, 50, and 100 μg/mL, diluted in Gibco RPMI 1640 medium, and the ARPC3/ARPC4 inhibitor CK666 (Abcam, Cambridge, UK) was used at concentrations of 25, 50, and 100 μM, diluted in DMSO. After 24 h of treatment, cell viability was assessed using a hemocytometer. The MAC-T cells were then seeded into 96-well plates. Following 24 h treatments with LPS, CK666, or their combination, 10 μL of CCK-8 (Selleck Chemicals, Houston, TX, USA) was added to each well. After incubating in the dark for 1–3 h, absorbance was measured at 450 nm using a microplate reader. The optimal concentrations of LPS and CK666 were selected for subsequent experiments.

### 2.3. Transcriptome Analysis

RNA purification was conducted with TriQuick isolation reagent (Solarbio, Beijing, China) following standard phenol–chloroform protocols. Nucleic acid quality verification was subsequently completed on an Agilent 4200 TapeStation platform. For functional annotation, we employed the KEGG ontology database to map metabolic pathways, coupled with genome-wide enrichment profiling using the GSEA algorithm (v4.3.2) for detecting coordinated gene expression patterns.

### 2.4. qRT-PCR

Total RNA isolation was performed on harvested cellular and tissue specimens utilizing a commercial RNA extraction solution (TriQuick Reagent, Solarbio, Beijing, China). Complementary DNA synthesis was subsequently carried out through reverse transcription employing the Evo M-MLV reverse transcription kit (Agbio, Changsha, Hunan, China) according to manufacturer specifications. Quantitative PCR amplification was implemented using a SYBR Green-based detection system (2x Master Mix, Selleck, Los Angeles, CA, USA) with thermal cycling performed on a LightCycler 96 instrument (Roche, Basel, Switzerland). Gene expression quantification was achieved through comparative threshold cycle analysis (2−ΔΔCT method) with normalization to β-actin.

### 2.5. Immunostaining

Samples were fixed with 4% paraformaldehyde for 30 min, permeabilized with 0.3% Triton X-100 for 10 min, and blocked with 5% BSA (Solarbio, Beijing, China) for 1 h. Primary antibodies were incubated overnight at 4 °C. Goat anti-rabbit IgG conjugated with Alexa Fluor 594 was incubated at 37 °C for 1 h. The 2-(4-Amidinophenyl)-6-indolecarbamidine dihydrochloride (DAPI) was used for nuclear staining. After sealing with anti-fade PVP (Boster, Wuhan, China), images were acquired using an inverted fluorescence microscope (Revolve Omega, ApexBio, Suzhou, China). For hematoxylin and eosin (H&E) staining, tissues were fixed in 4% paraformaldehyde, and tissue specimens were paraffin-encased using standard embedding protocols and precisely sectioned into 5 μm slices. Sequential deparaffinization was achieved through xylene immersion (2 × 10 min), followed by ethanol gradient rehydration (100–70%). Hematoxylin–eosin dual staining was executed using commercial reagents (Servicebio, Wuhan, China), with subsequent coverslip mounting via neutral gum medium (Sigma-Aldrich, St. Louis, MO, USA). Microscopic visualization was conducted on an Axiocam 208 color imaging system (Zeiss, Oberkochen, Germany) under standardized bright-field conditions. For immunohistochemical analysis, the avidin–biotin complex technique was implemented according to manufacturer specifications (Boster Biotechnology, Wuhan, China). Tissue antigen retrieval was followed by overnight incubation with primary antibodies at 4 °C, the subsequent application of biotinylated secondary antibodies (37 °C, 60 min), chromogenic development using a 3,3′-diaminobenzidine substrate, and sealing. A Zeiss microscope was used to obtain images.

### 2.6. Flow Cytometry

Following dual rinses with ice-cold phosphate-buffered saline, cellular specimens underwent enzymatic detachment and subsequent centrifugation at 25 °C. Apoptosis detection kits (BioLegend, San Diego, CA, USA) were used. The pelleted cells were reconstituted in 100 μL of diluted binding buffer and dual-stained with 5 μL FITC-conjugated Annexin V along with 5 μL propidium iodide working solution (PI). After 15 min of dark incubation at ambient temperature (25 ± 2 °C), the cellular suspensions received 400 μL supplemental staining buffer prior to quantitative apoptotic evaluation. Flow cytometric analysis (Beckman Coulter CytoFlex platform) was performed within 60 min post-staining to ensure signal fidelity.

### 2.7. Western Blot Analysis

Cellular and tissue lysates were prepared using ice-cold RIPA lysis buffer (Solarbio) containing protease/phosphatase inhibitors. Equal amounts of protein were separated by 12% SDS-PAGE and transferred to PVDF membranes. The membranes were blocked with rapid Sealing Solution (P0222, Beyotime, Shanghai, China) for 10–20 min at room temperature and incubated 10–12 h at 4 °C with primary antibodies against ARPC3, ARPC4, Bax, Bcl-2, HSPA1A, HSPA1L, Caspase7, IL-1β, TNF-α, IL-6, β-actin (Proteintech, Wuhan, China), IL-8 (Affinity Biosciences, Cincinnati, OH, USA), Caspase3, and Caspase8 (Bioss, Beijing, China). After incubation with secondary antibodies at 37 °C for 1 h, the membranes were washed three times with PBST (PBS containing 0.1% Tween 20). The relative expression of the target proteins was calculated from the gray values of the control β-actin using the β-actin internal reference antibody as a control, and the gray values of the bands were accurately analyzed by Image-J (Version 7.0) software. Western blot original images can be found in Supplementary Materials.

### 2.8. Detection of Mitochondrial Membrane Potential

JC-1 working fluid was prepared according to the instructions (E-CK-A301, Elabscience, China). When the mitochondrial membrane potential is high, JC-1 aggregates in the mitochondrial matrix in the form of polymers and emits red fluorescence, and the cells do not enter the apoptotic stage. In the early stage of apoptosis, the mitochondrial membrane potential decreased, JC-1 could not aggregate but dispersed in the cytoplasm in the form of a monomer and emitted green fluorescence. The cells were seeded into 6-well plates. After treating with DMSO, LPS, and CK666 for 24 h, the medium was discarded, and the plates were washed with PBS 3 times. In total, 1 mL basic medium and 1 mL JC-1 working solution were added into each well and incubated at 37 °C for 30 min in the dark. The supernatant was rinsed with 1 × JC-1 Assay Buffer three times, and 2 mL basal medium was added to each well. The images were acquired using an inverted fluorescence microscope (Revolve Omega 2.0, ApexBio, Suzhou, China).

### 2.9. Data Analysis

Data are presented as mean ± standard error of the mean. Statistical comparisons were performed using GraphPad Prism software (version 9.5; GraphPad, San Diego, CA, USA). One-way ANOVA was used for multiple group comparisons, and Student’s *t*-test was used for pairwise comparisons. A *p* value of <0.05 was considered statistically significant, and a *p* value of <0.01 was considered highly significant.

## 3. Results

### 3.1. Establishment of the LPS-Induced Mastitis Inflammation Model

**Figure 1:**
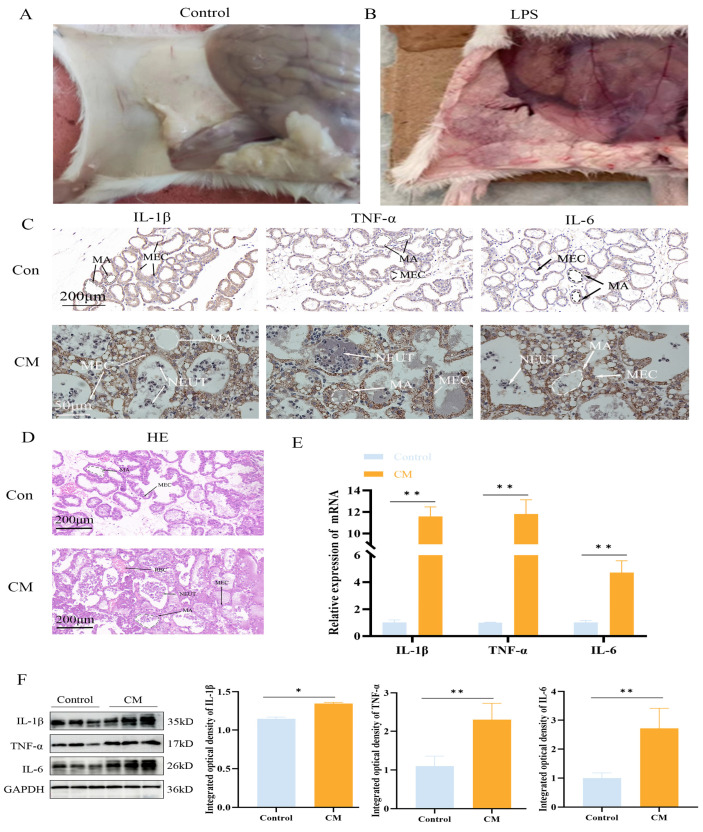
LPS induces an inflammatory response in mouse mammary glands. (**A**,**B**) Anatomical images of LPS-induced mammary gland tissue damage. (**C**,**D**) H&E staining and IHC staining for IL-1β, TNF-α, and IL-6. MA: mammary alveolus; MEC: mammary epithelial cell; NEUT: neutrophils; RBC: red blood cell. (**E**,**F**) Relative protein and mRNA expression levels of IL-1β, TNF-α, and IL-6 in mouse mammary tissue (n = 3); the IHC micrographs share identical magnification parameters. A representative scale bar is depicted on the left of panel C. Statistical significance was determined by comparison with the control group (* *p* < 0.05; ** *p* < 0.01). Note: CM, clinical mastitis.

**Figure 2:**
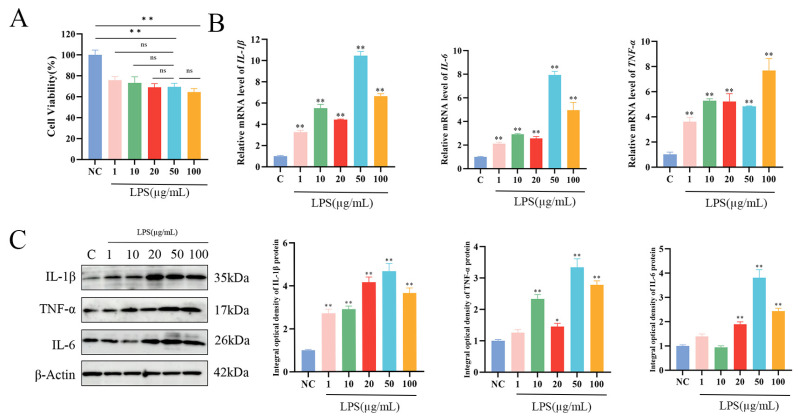
LPS-induced inflammation model in MAC-T cells. (**A**) Cell viability of MAC-T cells following 24 h of stimulation with different concentrations of LPS. (**B**,**C**) Relative protein and mRNA expression levels of IL-1β, TNF-α, and IL-6 in MAC-T cells after 24 h of stimulation with varying LPS concentrations (n = 3). Statistical significance was determined by comparison with the control group (* *p* < 0.05; ** *p* < 0.01; ns, not significant).

Post-infection, the mice were sacrificed, and their mammary glands were examined visually. In the control group, the mammary glands appeared smooth and flat, with no signs of congestion or swelling, while in the LPS group, the mammary glands exhibited significant congestion and swelling (Figure 1A,B). The H&E and IHC staining of mouse mammary tissue revealed that, in the control group, the alveolar wall structure was intact, and the epithelial cells were orderly arranged with no inflammatory cell infiltration. In contrast, LPS treatment resulted in extensive neutrophil infiltration, alveolar atrophy, deformation, and partial epithelial cell shedding (Figure 1C,D). IHC analysis further indicated a significant upregulation in the expression of IL-1β, TNF-α, and IL-6 in the LPS-treated group (Figure 1C). qPCR and Western blotting results from both mouse mammary tissue and MAC-T cells showed a significant increase in IL-1β, TNF-α, and IL-6 expression (*p* < 0.01) (Figure 1E), confirming the successful establishment of the model. This model of LPS-induced mastitis in a cell line is the source of the data in Figure 2 and was used as the basis for subsequent experiments.

Cells were divided into six groups based on LPS concentrations of 0, 1, 10, 20, 50, and 100 μg/mL. The toxicity of LPS to MAC-T cells was assessed using the CCK-8 assay. The results demonstrated that LPS concentrations of 1, 10, 20, 50, and 100 μg/mL significantly reduced MAC-T cell viability in a dose-dependent manner (*p* < 0.01). No statistically significant differences in cell viability were observed when comparing 50 μg/mL LPS with 1, 10, 20, or 100 μg/mL LPS concentrations. (Figure 2A). To further investigate the optimal LPS treatment concentration, the expression levels of inflammatory cytokines were analyzed by WB and qPCR. The mRNA and protein expression levels of IL-1β, TNF-α, and IL-6 in MAC-T cells were measured after treatment with different LPS concentrations, with the highest expression of IL-1β and IL-6 observed at 50 μg/mL (*p* < 0.01) (Figure 2B,C). Based on these findings, 50 μg/mL was selected as the optimal concentration for LPS treatment in subsequent experiments.

### 3.2. Transcriptome Analysis of LPS-Induced Gene Expression and Validation

**Figure 3:**
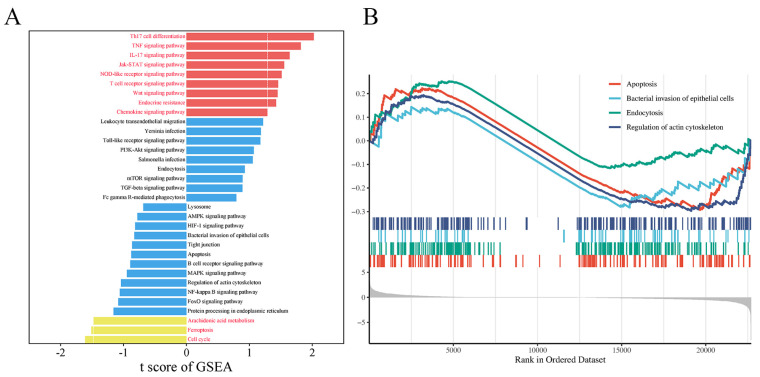
Visualization of transcriptomic data from LPS-treated MAC-T cells. (**A**) Selected pathways enriched by GSEA. (**B**) GSEA enrichment analysis of apoptosis, regulation of the actin cytoskeleton, bacterial invasion of epithelial cells, and endocytosis.

**Figure 4:**
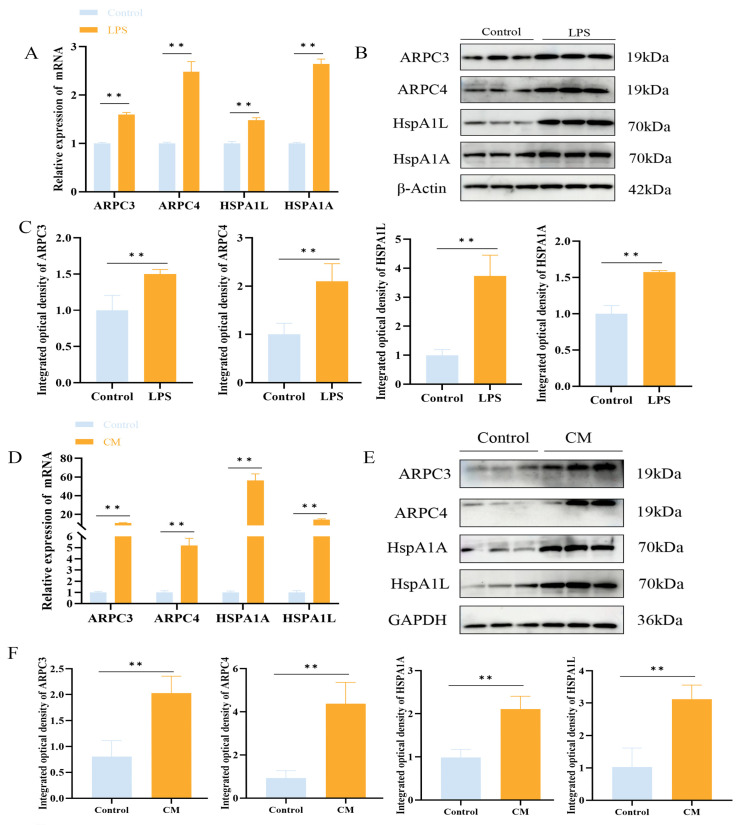
LPS upregulates ARPC3/ARPC4 and HSP70 to induce inflammation. (**A**–**C**) The relative expression levels of ARPC3, ARPC4 and HSP70 family in MAC-T cells were analyzed by Western blot and qRT-PCR. (**D**–**F**) Western blot and qRT-PCR were used to analyze the relative expression of *ARPC3*, *ARPC4* and *HSP70* family in mouse breast tissue (n = 3). Statistical significance was determined by comparison with the control group ( ** *p* < 0.01). Note: CM, clinical mastitis.

Transcriptomic analysis revealed 296 enriched pathways, containing a total of 22,699 genes. GSEA identified 32 genes enriched in the bacterial invasion of epithelial cells signaling pathway, with ARPC3 and ARPC4 showing significant upregulation. Additionally, 16 genes were enriched in the apoptosis pathway, with Caspase9 significantly upregulated and Bcl-2 significantly downregulated (Figure 3A,B). Previous proteomic data from our team suggested that ARPC3 and ARPC4 regulate the expression of Caspase family members (Caspase3 and Caspase7) through the modulation of the HSP70 family (HSPA1L and HSPA1A). In LTA-induced inflammation in MAC-T cells, ARPC3, ARPC4, HSPA1A, and HSPA1L were enriched in the endocytosis pathway. To explore the expression changes in ARPC3, ARPC4, and the HSP70 family in LPS-induced inflammation, qPCR and Western blotting were performed on MAC-T cells and mouse mammary tissue. The results showed that LPS treatment significantly upregulated the mRNA and protein expression of ARPC3, ARPC4, HSPA1A, and HSPA1L (*p* < 0.01) (Figure 4A–F).

### 3.3. LPS Promotes Apoptosis in MAC-T Cells

**Figure 5:**
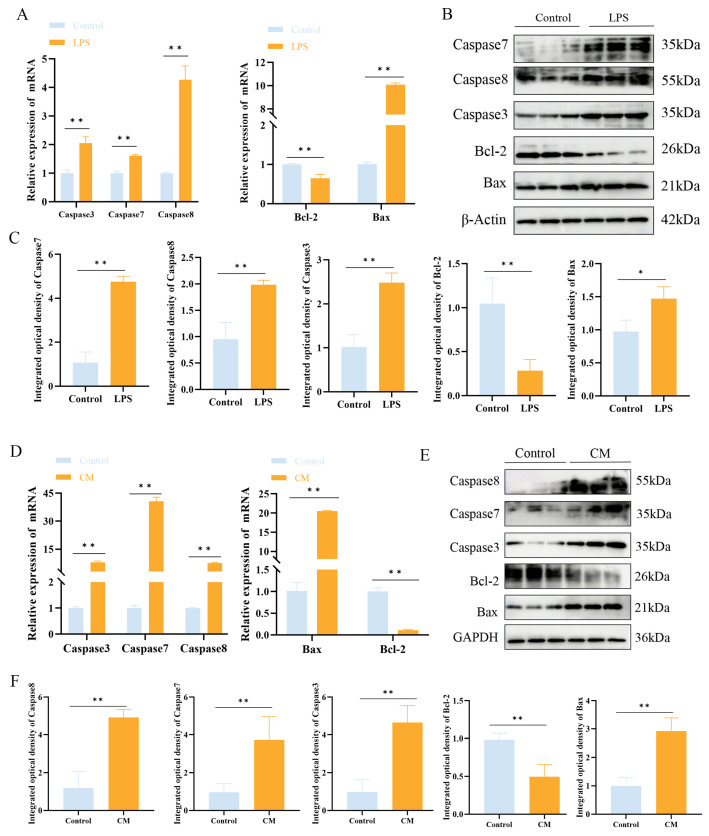
The effect of LPS treatment on the expression of apoptosis-related factors in MAC-T cells. (**A**–C) Relative mRNA (**A**) and protein (**B**,**C**) expression levels of Caspase3, Caspase7, Caspase8, Bax, and Bcl-2 in MAC-T cells following LPS treatment. (**D**–**F**) Relative mRNA (**D**) and protein (**E**,**F**) expression levels of Caspase3, Caspase7, Caspase8, Bax, and Bcl-2 in mouse mammary tissue following LPS treatment (n = 3). Statistical significance was determined by comparison with the control group (* *p* < 0.05; ** *p* < 0.01). Note: CM, clinical mastitis.

Western blotting and qPCR were used to examine the impact of LPS on apoptosis in MAC-T cells and mouse mammary tissue. The Western blotting and qPCR results showed that LPS treatment upregulated the expression of apoptotic markers Bax, Caspase3, Caspase7, and Caspase8, while downregulating Bcl-2 expression (*p* < 0.01) (Figure 5A–D).

### 3.4. The Effect of Inhibiting ARPC3/ARPC4 on Apoptosis and Inflammation of MAC-T Cells

**Figure 6:**
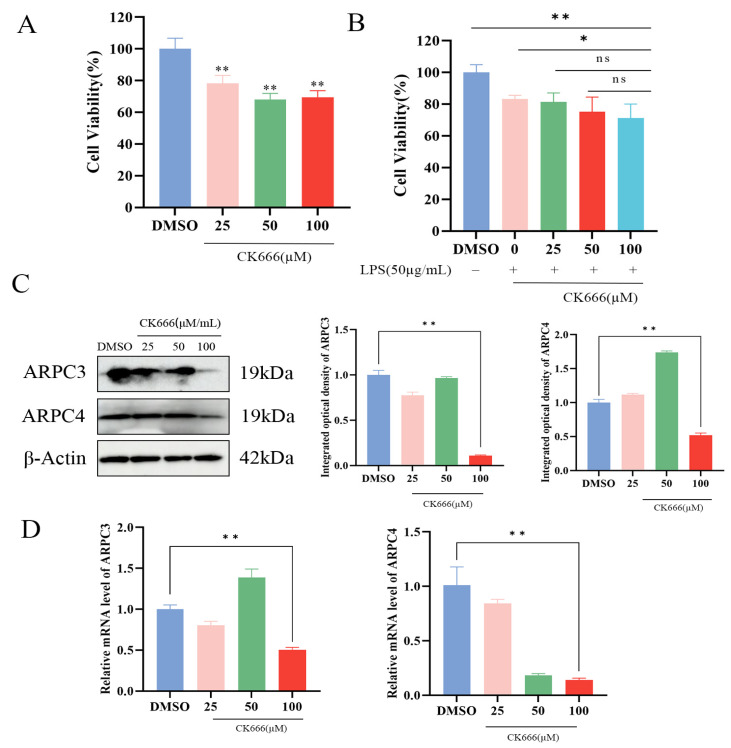
CK666 concentration screening. (**A**,**B**) Cell viability of MAC-T cells treated with different concentrations of CK666 and CK666 + LPS, as assessed by the CCK-8 assay. (**C**) Western blot analysis of the relative protein expression levels of ARPC3/ARPC4 in MAC-T cells treated with varying concentrations of CK666. (**D**) qRT-PCR analysis of the relative mRNA expression levels of *ARPC3*/*ARPC4* in MAC-T cells treated with different concentrations of CK666 (n = 3). Statistical significance was determined by comparison with the DMSO group (* *p* < 0.05; ** *p* < 0.01; ns, not significant).

In order to explore the effect of inhibiting the expression of ARPC3/ARPC4 on the apoptosis and inflammatory factors of MAC-T cells, CK666 was used to treat cells to inhibit the expression of ARPC3/ARPC4. Cells were divided into two groups: the control group (treated with LPS + DMSO) and the experimental group (treated with LPS + CK666). The effects of CK666 (0, 25, 50, and 100 μM) on MAC-T cell viability were evaluated using the CCK-8 assay. The results indicated that CK666 showed some toxicity to MAC-T cells, but cell viability remained above 60% (Figure 6A,B). qPCR and Western blotting were used to assess the inhibitory effects of CK666 on ARPC3 and ARPC4 expression. Compared to the DMSO-treated group, treatment with 100 μM CK666 significantly reduced the expression of ARPC3 and ARPC4 in MAC-T cells (Figure 6C,D). The CCK-8 assay also demonstrated that the combination of 100 μM CK666 with 50 μg/mL LPS maintained relatively high cell viability (Figure 6B), which served as the foundation for subsequent experiments (Figure 6C,D).

**Figure 7:**
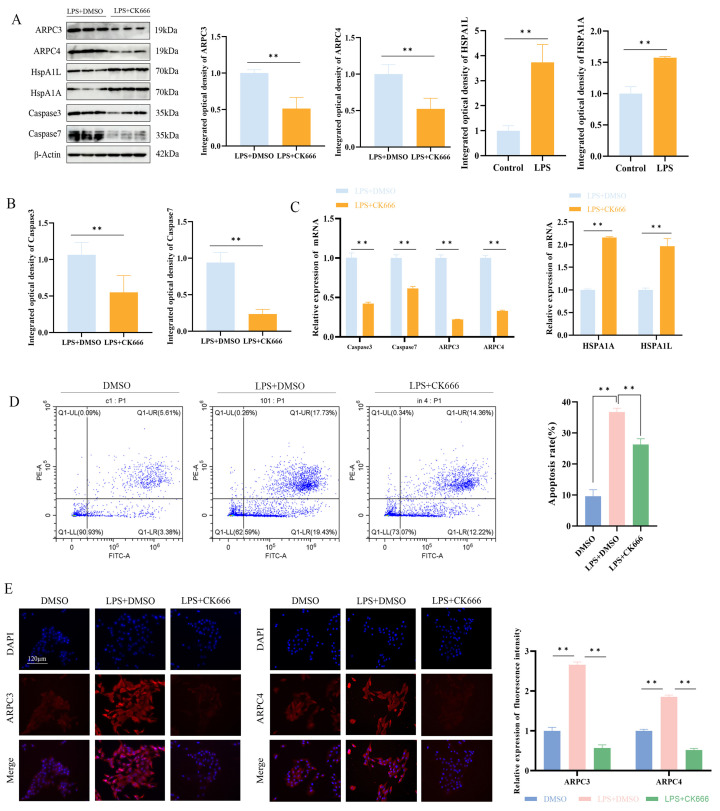
Effect of ARPC3/ARPC4 inhibition on HSP70 expression and cell apoptosis. (**A**–**C**) Relative protein and mRNA expression levels of ARPC3/ARPC4, HSP70, Caspase3, and Caspase7 in LPS-induced MAC-T cells following CK666 treatment. (**D**) Flow cytometry analysis of apoptosis rates in cells treated with CK666 and LPS. (**E**) Localization of ARPC3/ARPC4 expression in MAC-T cells (scale: 120 μm) (n = 3). All micrographs share identical magnification parameters. A representative scale bar (120 μm) is depicted in the higher left corner of panel E. Statistical significance was determined by comparison with the LPS + DMSO group ( ** *p* < 0.01).

The Western blotting and qPCR results showed that, compared to the LPS + DMSO group, the LPS + CK666 treatment significantly inhibited the expression of ARPC3 and ARPC4 in MAC-T cells and markedly reduced the cell apoptosis rate (*p* < 0.01) (Figure 7A–C). Additionally, qPCR and Western blotting analysis revealed that the expression of apoptosis-related genes Caspase3 and Caspase7 was significantly decreased in the LPS + CK666 group (*p* < 0.01) (Figure 7A,B). The mRNA and protein levels of HSPA1A and HSPA1L in the LPS + CK666 treatment group were significantly higher than in the LPS + DMSO group (*p* < 0.01) (Figure 7A,B). Immunofluorescence localization analysis showed that ARPC3 and ARPC4 were predominantly localized in the cytoplasm. Compared with the LPS + DMSO group, the cytoplasmic fluorescence intensity of ARPC3/4 was significantly decreased after the LPS + CK666 intervention (Figure 7D).

**Figure 8:**
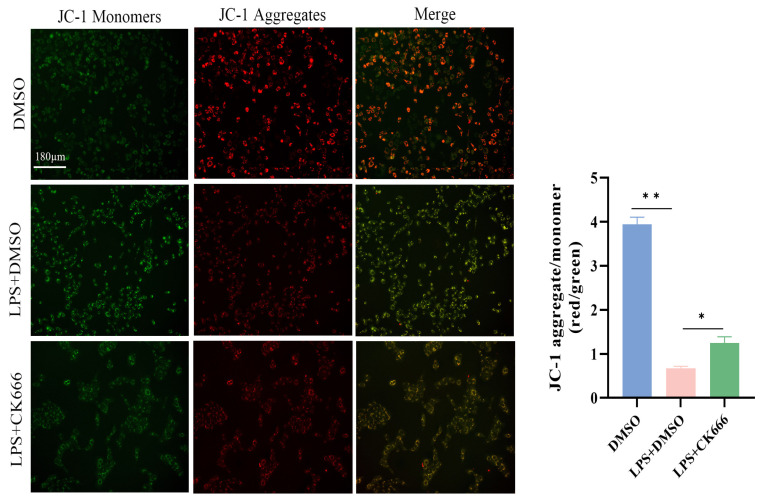
The JC-1 fluorescent probe was used to detect the effect of inhibiting ARPC3/ARPC4 on the mitochondrial membrane potential of MAC-T cells (scale: 180 μm). All micrographs share identical magnification parameters. A representative scale bar (180 μm) is depicted in the higher left corner of the panel. Statistical significance was determined by comparison with the DMSO group (* *p* < 0.05; ** *p* < 0.01).

To further explore the effect of ARPC3/ARPC4 on mitochondrial membrane potential in MAC-T cells, JC-1 probe detection showed that compared with the DMSO group, the J-aggregate intensity of the LPS + DMSO group was reduced and the monomer was significantly enhanced, suggesting that the mitochondrial membrane potential was depolarized. Notably, CK666 treatment significantly reduced the green fluorescence intensity after LPS stimulation, while the red fluorescence did not change significantly, indicating that the inhibitor could partially reverse the LPS-induced decrease in membrane potential (Figure 8).

**Figure 9:**
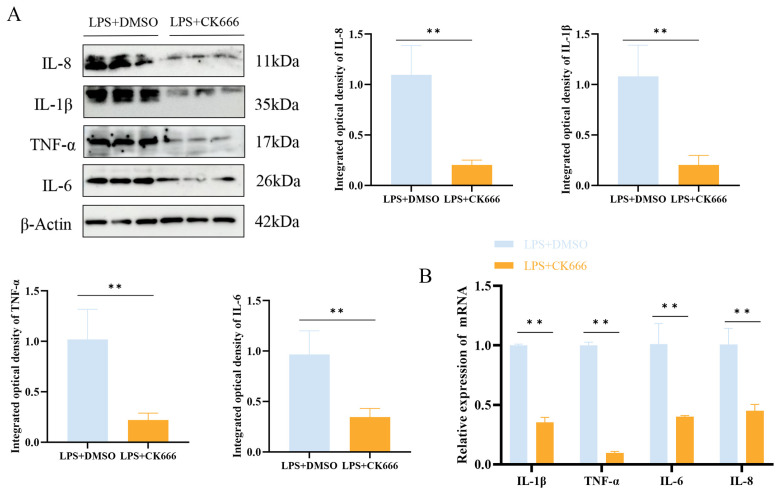
The inhibition of ARPC3/ARPC4 regulates inflammatory factor expression. (**A**) Western blot analysis of the relative protein expression levels of IL-1β, TNF-α, IL-6, and IL-8 following CK666 treatment. (**B**) qRT-PCR analysis of the relative mRNA expression levels of *IL-1β*, *TNF-α*, *IL-6*, and *IL-8* after CK666 treatment (n = 3). Statistical significance was determined by comparison with the LPS + DMSO group (** *p* < 0.01).

LPS treatment promoted the secretion of IL-1β, TNF-α, IL-6, and IL-8 in MAC-T cells, thereby inducing an inflammatory response. To investigate the impact of ARPC3/ARPC4 on inflammatory cytokines, the levels of IL-1β, TNF-α, IL-6, and IL-8 were measured after CK666 treatment in the LPS-treated group. The results revealed a significant reduction in the expression of these cytokines (*p* < 0.05) (Figure 9).

## 4. Discussion

The epithelial cell barrier, structurally based on tight junctions, is vital for blocking the entry of toxins and bacteria, serving as a crucial defense for mammary health [31]. When host cells are invaded by bacteria, virulence factors activate Arp2/3-mediated actin cytoskeleton rearrangement, disrupting tight junctions and facilitating the entry of external toxins [32]. The knockdown of ARPC3 leads to tight junction dysfunction in cells [13,15], while the knockdown of ARPC4 causes skin complications similar to psoriasis [33]. Gene Set Enrichment Analysis (GSEA) revealed that 132 pathways were activated and 163 pathways inhibited, primarily related to inflammation and immune responses. Given that this study focused on the interplay between inflammatory reactions and apoptosis, we prioritized the analysis of IL-1β, TNF-α, and IL-8, which are closely linked to these processes. The apoptosis-related pathways involving Bcl-2 and Bax were experimentally validated. Thirty-two differentially expressed genes were enriched in the bacterial invasion of epithelial cells signaling pathway. Notably, ARPC3/ARPC4, key regulators of the cytoskeleton, were significantly enriched in this pathway, suggesting that LPS-induced inflammation in MAC-T cells may upregulate ARPC3/ARPC4, activating the bacterial invasion signaling pathway, which influences endocytosis and disrupts the epithelial barrier, promoting inflammation and apoptosis. Furthermore, in bovine mammary tissues, we demonstrated the regulatory roles of ARPC3/ARPC4, HSP70, and NLRP3 in mediating inflammatory and apoptotic responses [34]. Our prior research confirmed the expression patterns and functional roles of the enriched endocytosis-associated genes: ARPC3/ARPC4 were enriched in the endocytosis pathway, and the inhibition of Arp2/3 complex activity impaired endocytosis in the body [35]. The dysfunction in endocytosis and epithelial barrier impairment caused by ARPC3/ARPC4 further promotes bacterial toxin entry, accelerating apoptosis. Our previous research showed that LTA treatment upregulated both ARPC3/ARPC4 and HSP70 expression, indicating that HSP70 has a protective role in host cells during inflammation. However, the potential interaction between ARPC3/ARPC4 and HSP70 has not been previously reported [28,29].

After the LPS treatment of MAC-T cells, the expression of both ARPC3/ARPC4 and HSP70 was significantly upregulated, consistent with our previous findings [28,29]. This suggests a similar expression pattern occurs in both Gram-positive and Gram-negative bacterial infections, making this pathway a key mediator of bacterial invasion. To investigate whether ARPC3/ARPC4 interacts with HSP70, we added CK666 to the LPS-induced group to inhibit ARPC3/ARPC4. The results showed that inhibiting ARPC3/ARPC4 led to a significant upregulation of HSP70 expression. The actin cytoskeleton rearranged by the Arp2/3 complex plays an essential role in maintaining cell migration, phagocytosis, and apoptosis. Changes in Arp2/3 activity affect the formation of branched actin filaments, leading to the rearrangement of the cytoskeleton, reduced cell proliferation, and increased apoptosis [20]. As core subunits of the Arp2/3 complex, ARPC3/ARPC4 are crucial for its nucleating activity. Interestingly, HSP70 protects cells from apoptosis caused by toxins by inhibiting inflammation. Studies have shown that elevated HSP70 levels can counteract the increased cell permeability induced by bacterial toxins by modulating actin cytoskeleton activity, thus protecting host cells from bacterial toxin-induced apoptosis and inflammation [36,37]. Therefore, when bacterial toxins invade host cells, they upregulate ARPC3/ARPC4, altering Arp2/3 activity and disrupting the epithelial cell barrier. At the same time, the ARPC3/ARPC4 inhibition of HSP70 expression weakens its protective effect, promoting toxin entry and triggering cell apoptosis and inflammation [38].

Inflammation induces cell apoptosis, and the accumulation of apoptotic cells further exacerbates inflammation. In this study, LPS treatment upregulated apoptosis rates and the expression of apoptosis-related genes. We found that inhibiting ARPC3/ARPC4 reversed the high expression of Caspase3/Caspase7 and the increased apoptosis rate induced by LPS. This may be due to the weakened inhibitory effect of CK666 on ARPC3/ARPC4 in the presence of LPS, resulting in higher HSP70 expression. Elevated HSP70 directly inhibits the activation of Caspase3 and Caspase7, interrupting the Caspase cascade and binding to Bax to prevent its translocation to the mitochondria, thus reducing apoptosis [39,40]. Previous studies have suggested that the high bioactivity of the Arp2/3 complex in the kidneys may serve as a marker of kidney inflammation [41]. Thus, the Arp2/3 complex is linked to inflammation development, but its role in mastitis has not been documented. Our experimental results show that inhibiting ARPC3/ARPC4 significantly decreased the expression of inflammatory cytokines. On one hand, this indicates that inhibiting ARPC3/ARPC4 activity can reduce abnormal actin cytoskeleton activity, protect the epithelial barrier, reduce LPS uptake, and suppress apoptosis and inflammation [42]. On the other hand, the lack of ARPC3/ARPC4 inhibition of HSP70 expression, coupled with higher HSP70 levels, directly inhibits the LPS activation of IL-6 and TNF-α, and HSP70 also inhibits NF-κB activation, reducing the release of IL-1β, IL-8, and TNF-α. The reduction in inflammatory cytokines further reduces apoptosis [43]. In conclusion, LPS promotes inflammation and apoptosis by upregulating ARPC3/ARPC4 and inhibiting HSP70. While our study primarily focused on HSP70 as a key mediator, the complexity of inflammatory responses likely involves crosstalk with other stress response pathways. HSP70 is known to modulate endoplasmic reticulum (ER) stress through PERK-eIF2α signaling, and its upregulation in our model may concurrently alleviate ER stress-induced apoptosis [44]. Furthermore, the observed recovery of mitochondrial membrane potential following CK666 treatment suggests potential coordination between HSP70 and reactive oxygen species (ROS)-scavenging systems. Future studies should explore these multidimensional interactions to further elucidate the molecular mechanisms by which ARPC3/ARPC4 and HSP70 regulate bovine mastitis progression. Although CK666 is widely utilized to inhibit ARPC3/ARPC4, its off-target effects in mammary epithelial cells remain insufficiently explored. We will delve deeper into solving this problem in subsequent experiments.

Therefore, the targeted inhibition of ARPC3/ARPC4 may have dual therapeutic benefits in mastitis management: it inhibits bacterial invasion by blocking actin-driven internalization and reduces LPS-induced apoptosis and inflammatory cascades in bovine mammary epithelial cells. It is worth noting that the upregulation of HSPA1A/HSPA1L after CK666 treatment emphasizes a compensatory cytoprotective response that may be an adjuvant treatment strategy for mastitis. Future research should explore the synergistic effect of ARPC3/ARPC4 inhibitors and HSP70 inducers in the clinical treatment of mastitis. However, CK666 has a certain toxicity, and its potential impact on normal breast epithelial function deserves careful evaluation.

**Figure 10:**
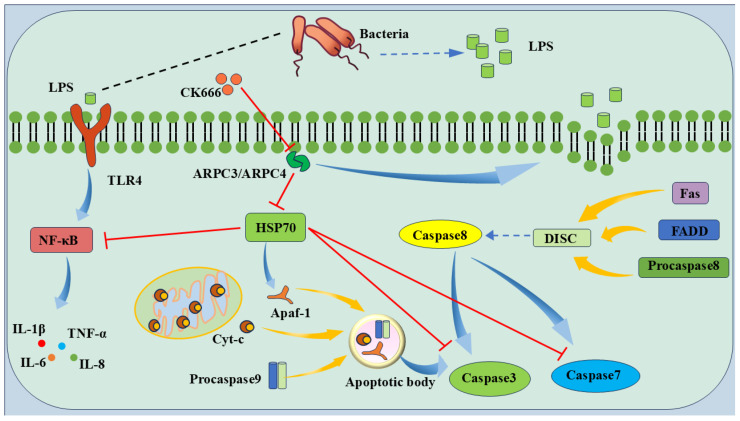
Model pattern of LPS-induced apoptosis via ARPC3/ARPC4/HSP70. The ARPC3/ARPC4 complex promotes host cell apoptosis by targeting HSP70. LPS destroys the epithelial cell barrier and inhibits the expression of HSP70 to reduce the protective effect of HSP70 on host cells, thereby promoting the development of cell inflammation and apoptosis.

The experimental results show that the ARPC3/ARPC4-targeted inhibition of HSP70 expression promotes inflammation and apoptosis. A detailed mechanistic model based on these findings is presented in Figure 10. When Gram-negative bacteria infect host cells, LPS release from the bacterial cell wall upregulates ARPC3/ARPC4, leading to actin polymerization and promoting LPS uptake. LPS binds to TLR4, activating the NF-κB pathway and releasing IL-1β, TNF-α, IL-6, and IL-8 [45]. At the same time, LPS induces mitochondrial damage, releasing Cyt-C, which forms an apoptosome with Apaf-1 and Procaspase9 to activate Caspase3 [46]. The DISC complex, composed of Fas, FADD, and Procaspase-8, activates Caspase8, which in turn activates Caspase3 and Caspase7, leading to apoptosis [47]. The upregulation of ARPC3/ARPC4 inhibits HSP70 expression, preventing it from inhibiting Caspase3 and Caspase7 activation, and obstructs HSP70 binding to Apaf-1, disrupting apoptosome formation and promoting apoptosis [48].

## 5. Conclusions

In the LPS-induced mastitis model, the expression of ARPC3/ARPC4 and HSP70 is positively correlated with the progression of inflammation and cell apoptosis. Our previous research suggested a possible direct interaction between ARPC3/ARPC4 and HSP70. In this study, inhibiting ARPC3/ARPC4 upregulated HSP70 expression, reduced apoptosis rates, and decreased inflammatory cytokine secretion, providing preliminary evidence that LPS induces inflammation and apoptosis by upregulating ARPC3/ARPC4 and inhibiting HSP70. However, the exact nature of the interaction between ARPC3/ARPC4 and HSP70, whether direct or indirect, remains unclear and warrants further investigation.

## Data Availability

The datasets analyzed or generated during the study are available from the authors (dongwt@gsau.edu.cn).

## Supplementary Materials

The following supporting information can be downloaded at: https://www.mdpi.com/article/10.3390/biom15040538/s1, Western blot original images.
