# Supplementary material for: Targeted Regulation of HSP70 by the ARP2/3 Complex in Mammary Epithelial Cells and Its Impact on Host Cell Apoptosis
Source: Biomolecules. 2025 Apr 7;15(4):538. doi: 10.3390/biom15040538 (PMC12025207; doi:10.3390/biom15040538)
Supplement: Supplementary file 1 [file biomolecules-15-00538-s001.zip › original image/original image1.pptx]

## Slide 1
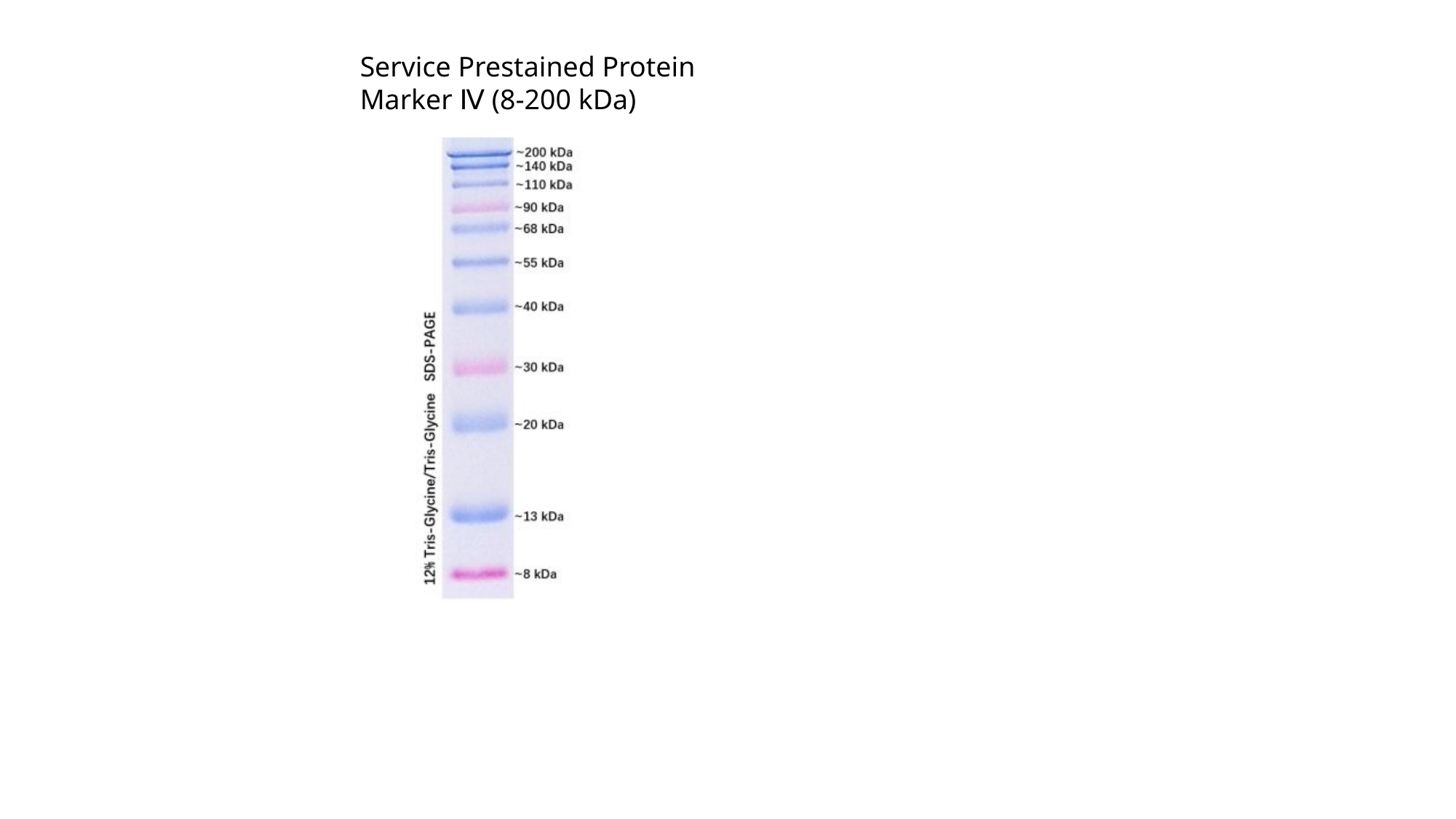

Service Prestained Protein Marker Ⅳ (8-200 kDa)

## Slide 2
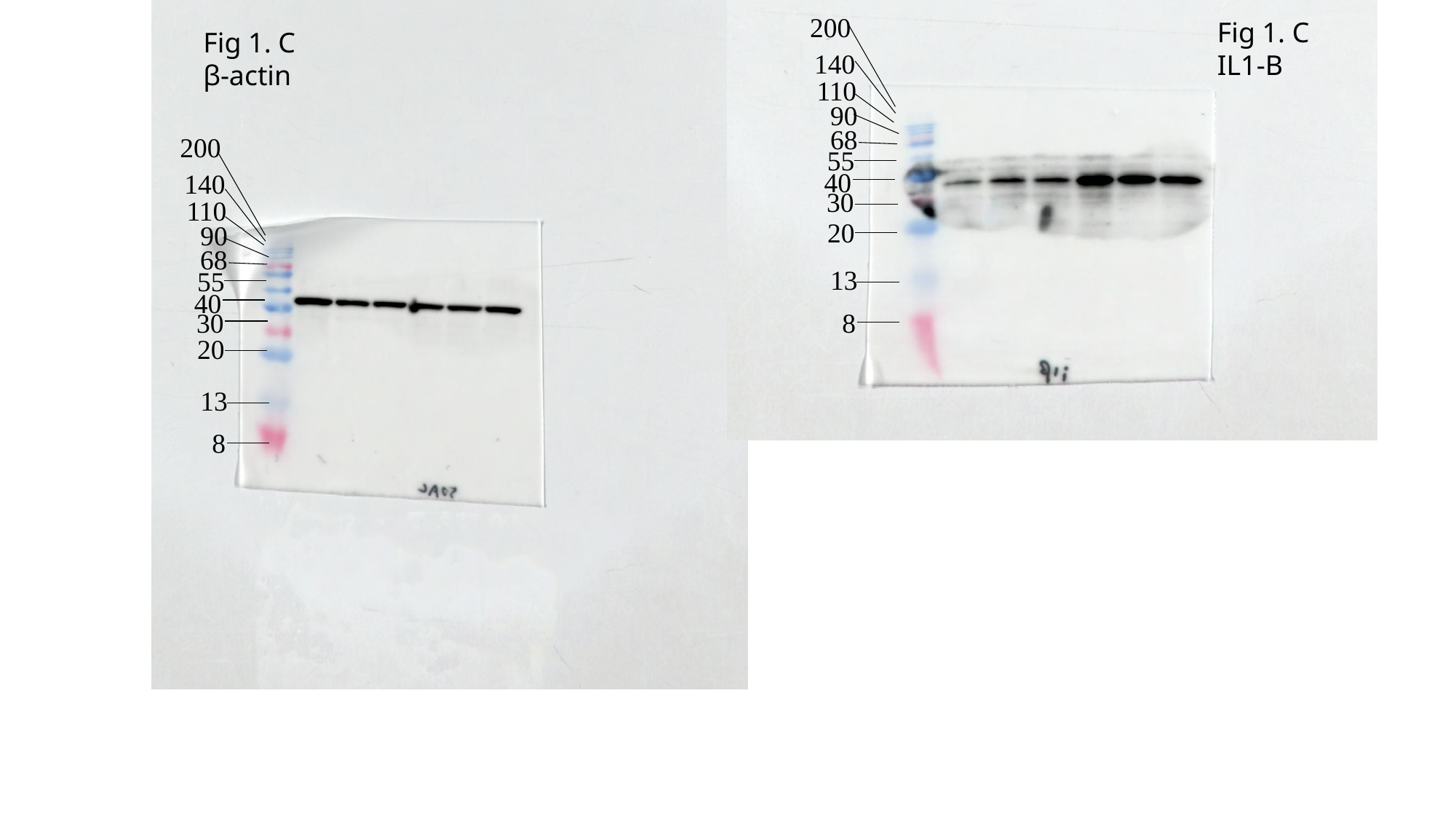

200
Fig 1. C
IL1-Β
Fig 1. C
β-actin
140
110
90
68
200
55
40
140
30
110
20
90
68
13
55
40
30
8
20
13
8

## Slide 3
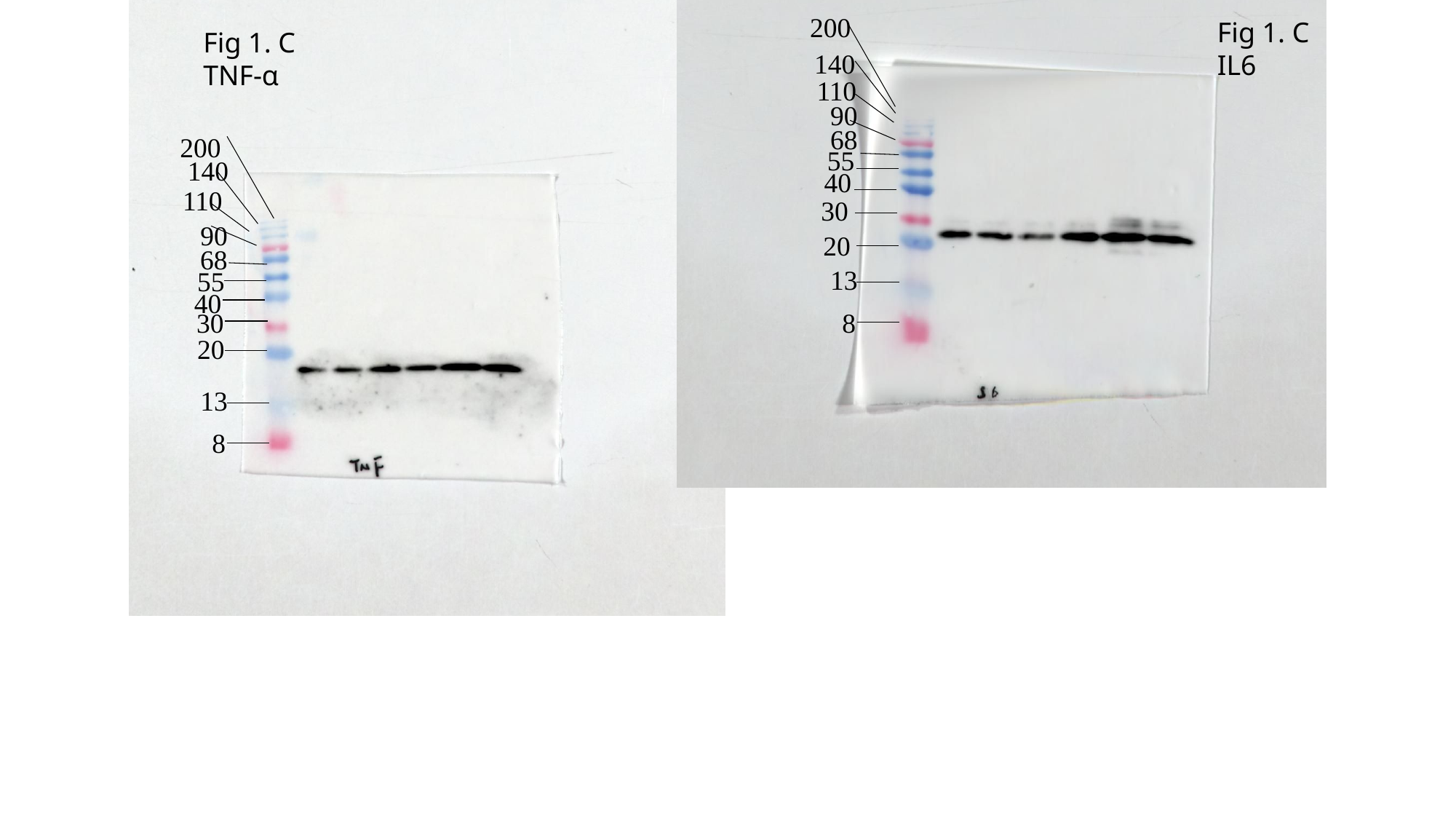

200
Fig 1. C
IL6
Fig 1. C
TNF-α
140
110
90
68
200
55
140
40
110
30
90
20
68
13
55
40
30
8
20
13
8

## Slide 4
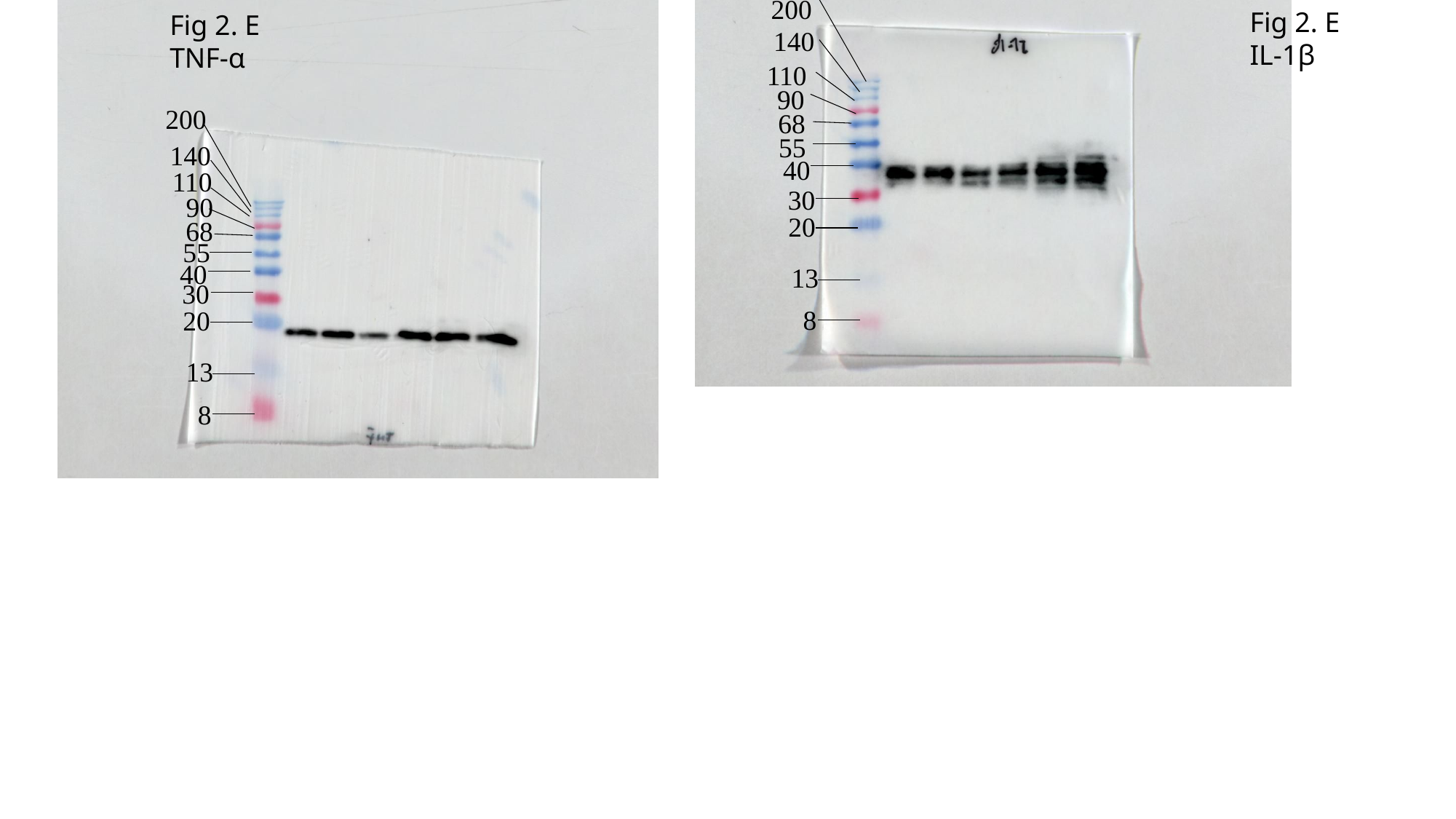

Fig 2. E
IL-1β
200
Fig 2. E
TNF-α
140
110
90
200
68
55
140
40
110
30
90
20
68
55
40
13
30
8
20
13
8

## Slide 5
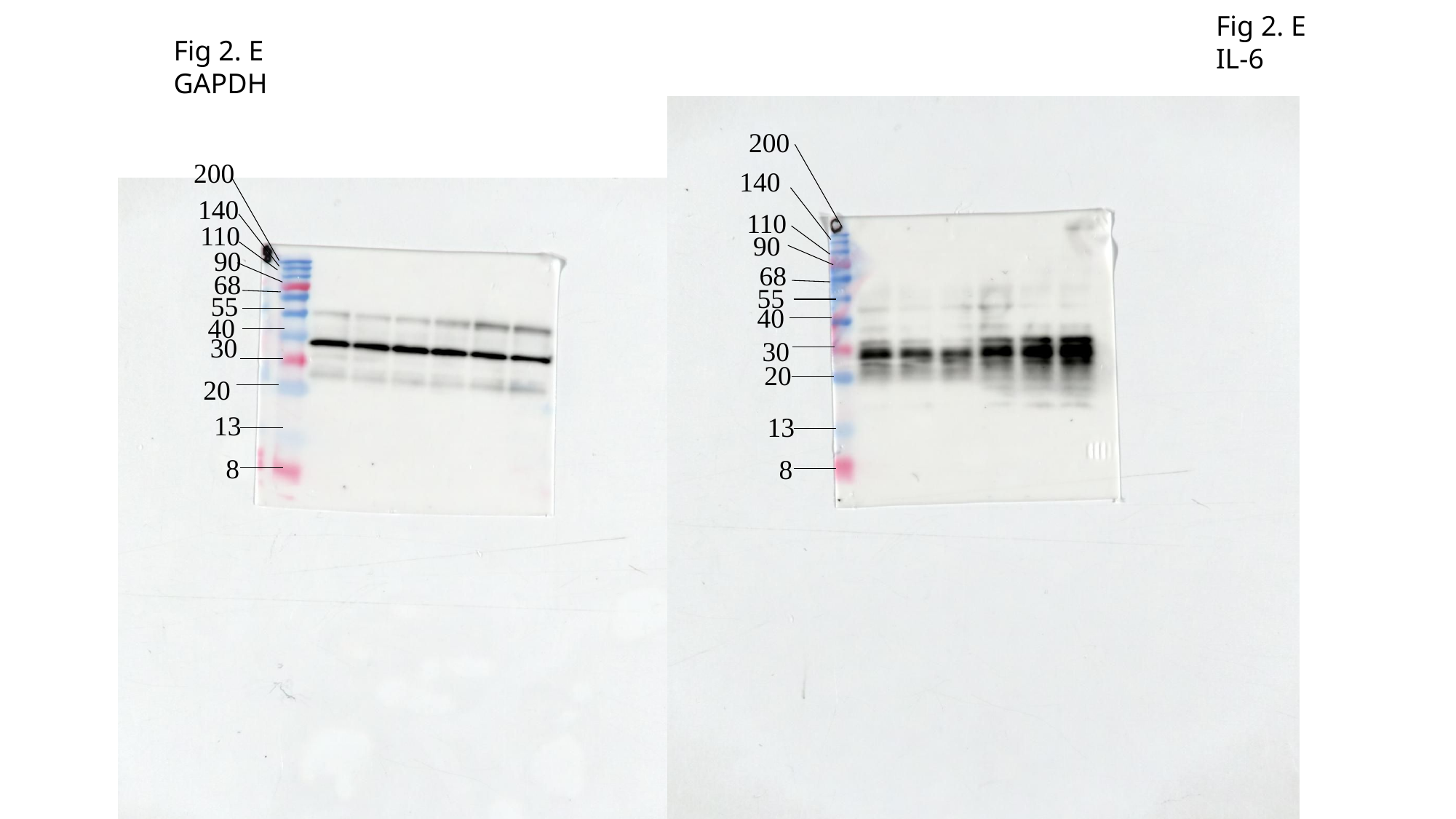

Fig 2. E
IL-6
Fig 2. E
GAPDH
200
200
140
140
110
110
90
90
68
68
55
55
40
40
30
30
20
20
13
13
8
8

## Slide 6
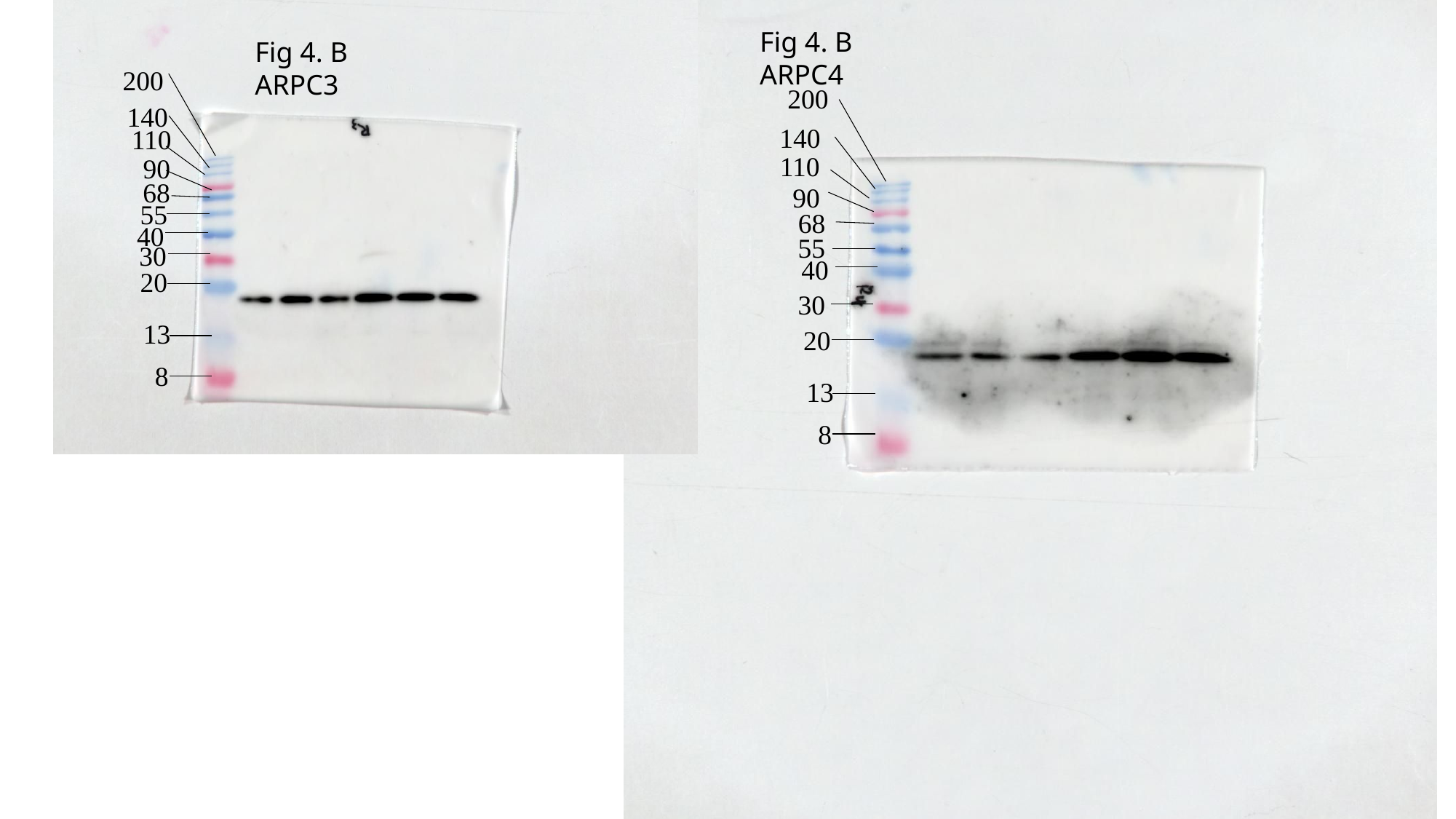

Fig 4. B
ARPC4
Fig 4. B
ARPC3
200
200
140
140
110
110
90
68
90
55
68
40
55
30
40
20
30
13
20
8
13
8

## Slide 7
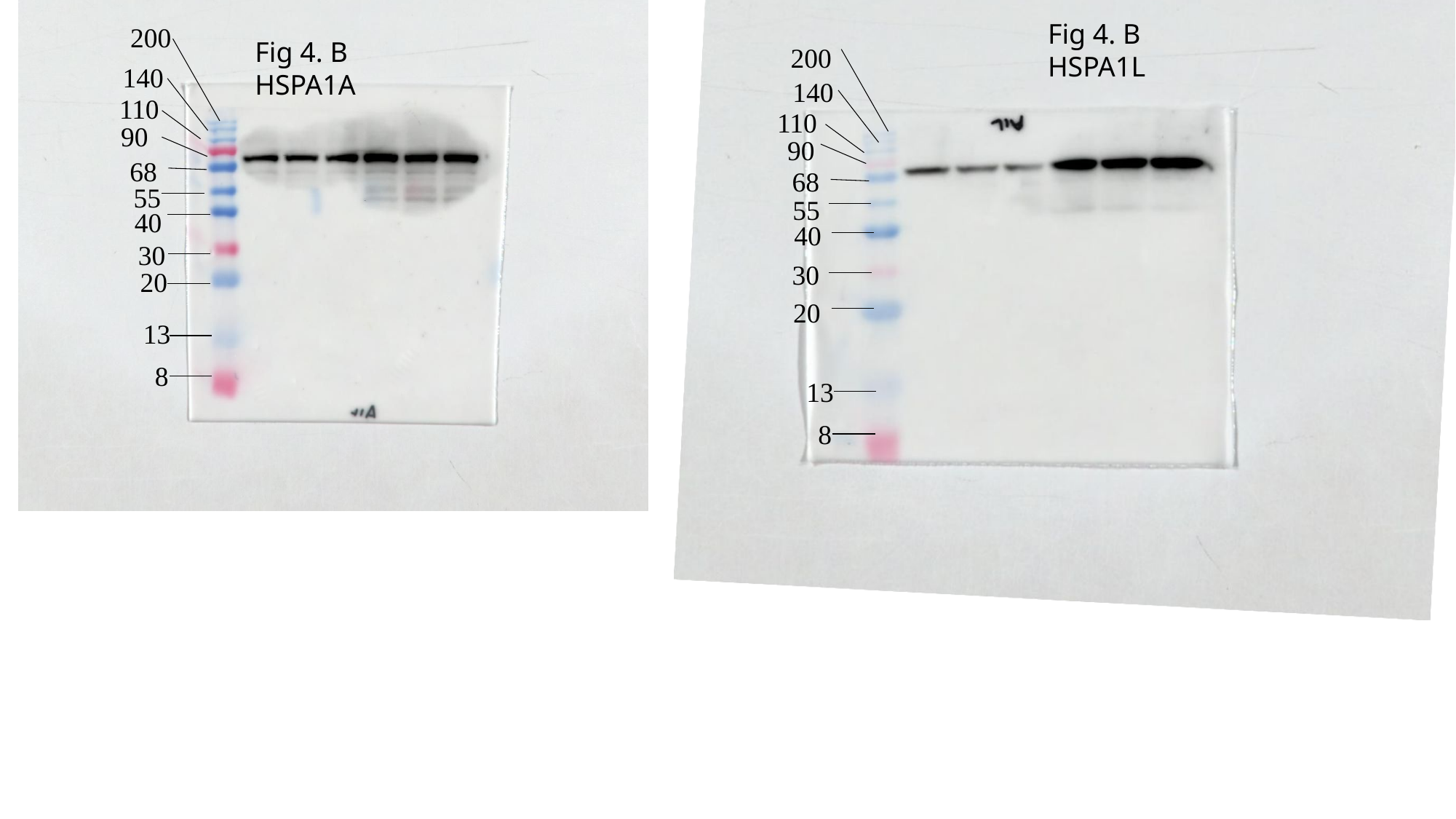

Fig 4. B
HSPA1L
200
Fig 4. B
HSPA1A
200
140
140
110
110
90
90
68
68
55
55
40
40
30
30
20
20
13
8
13
8

## Slide 8
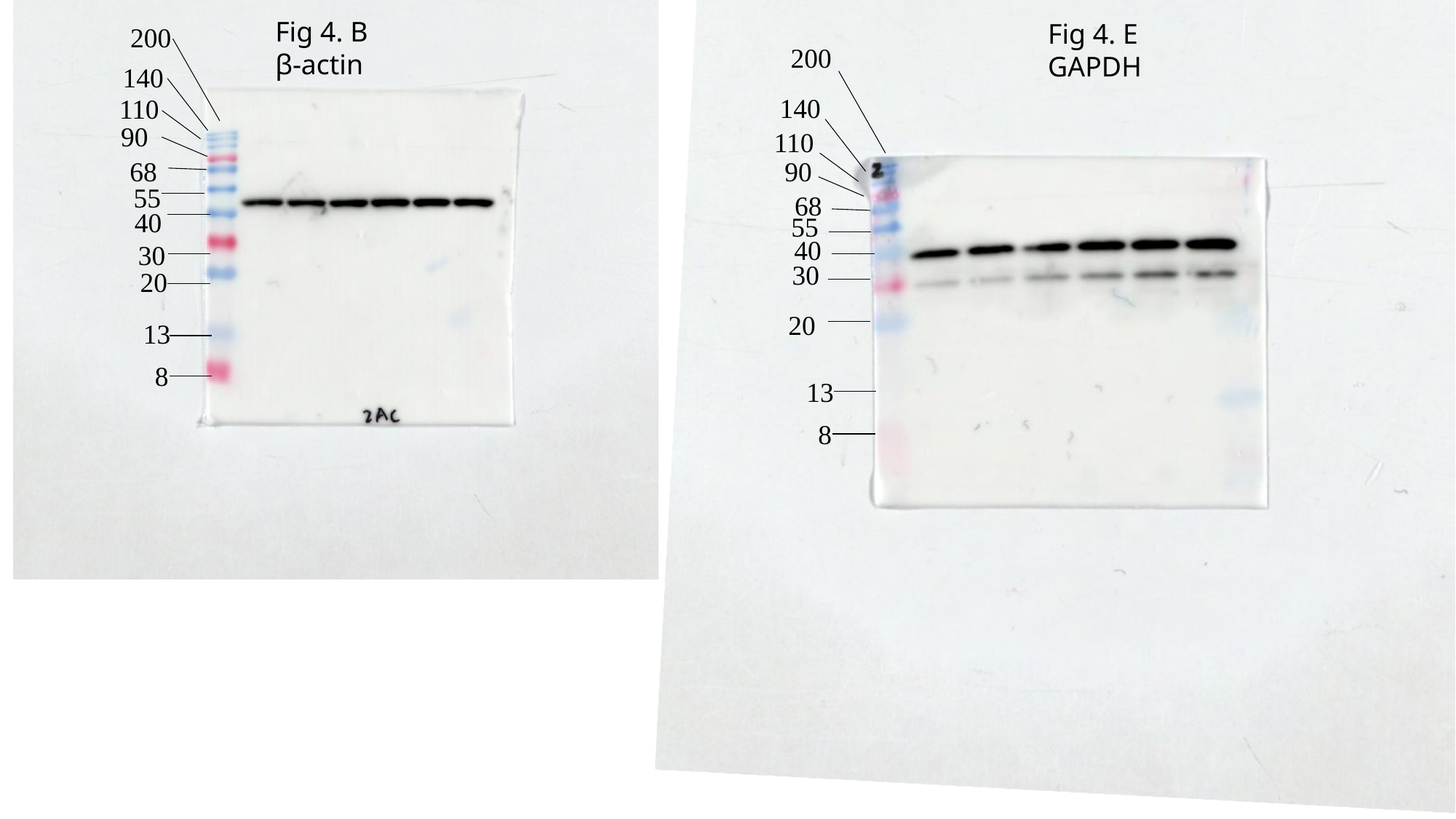

Fig 4. B
β-actin
Fig 4. E
GAPDH
200
200
140
140
110
90
110
68
90
55
68
40
55
40
30
30
20
20
13
8
13
8

## Slide 9
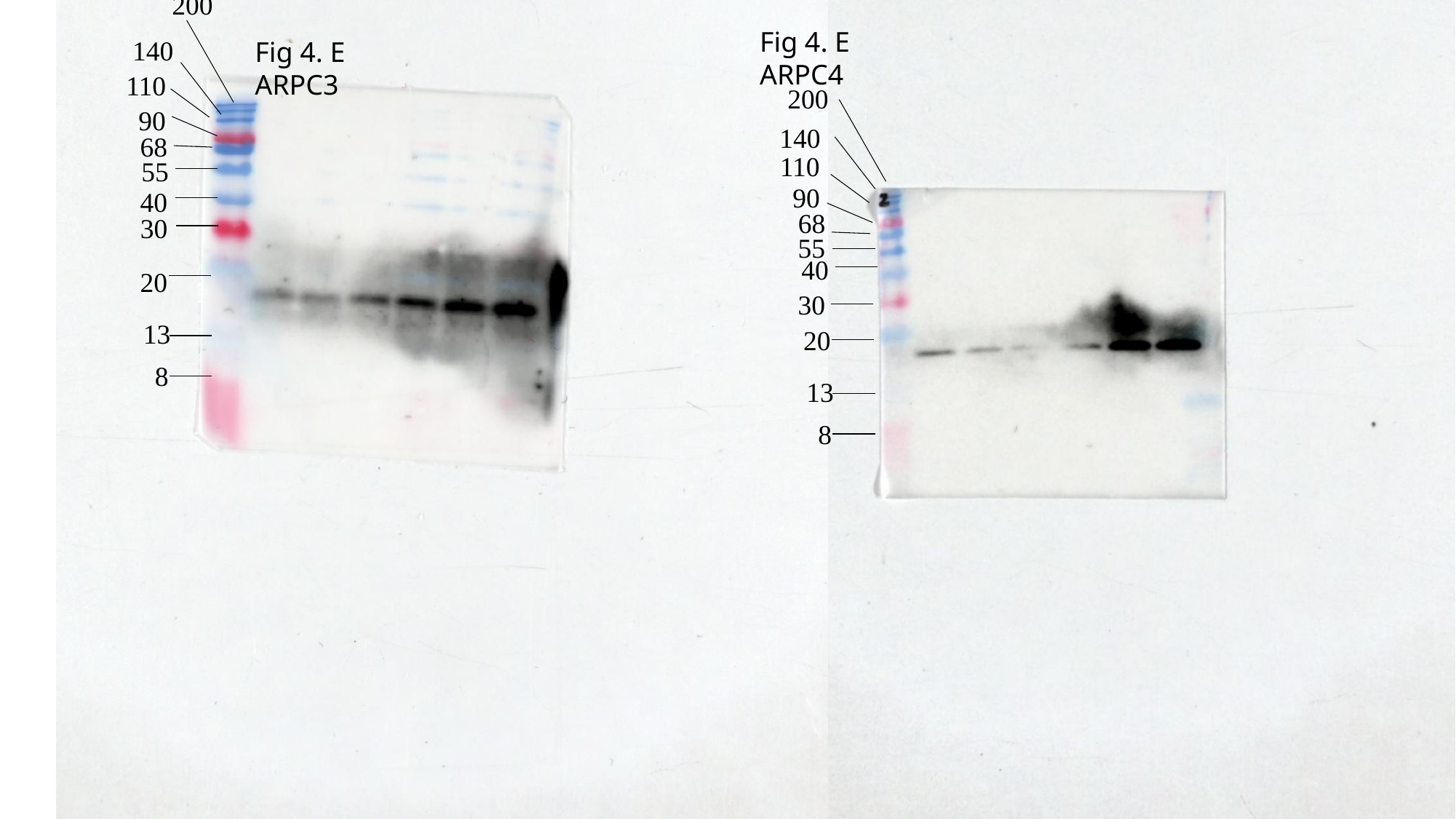

200
Fig 4. E
ARPC4
140
Fig 4. E
ARPC3
110
200
90
140
68
110
55
90
40
68
30
55
40
20
30
13
20
8
13
8

## Slide 10
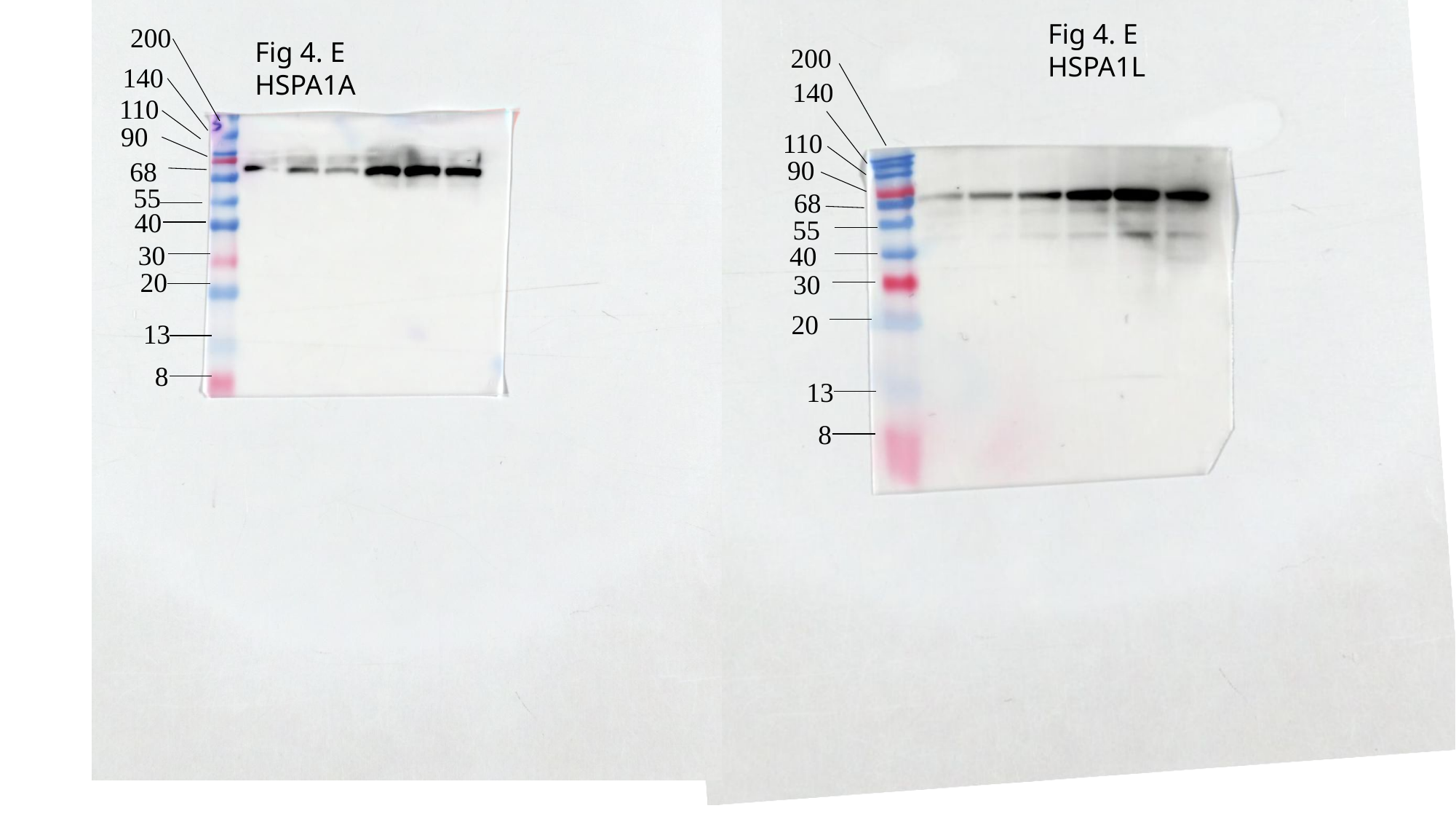

Fig 4. E
HSPA1L
200
Fig 4. E
HSPA1A
200
140
140
110
90
110
90
68
55
68
40
55
30
40
20
30
20
13
8
13
8

## Slide 11
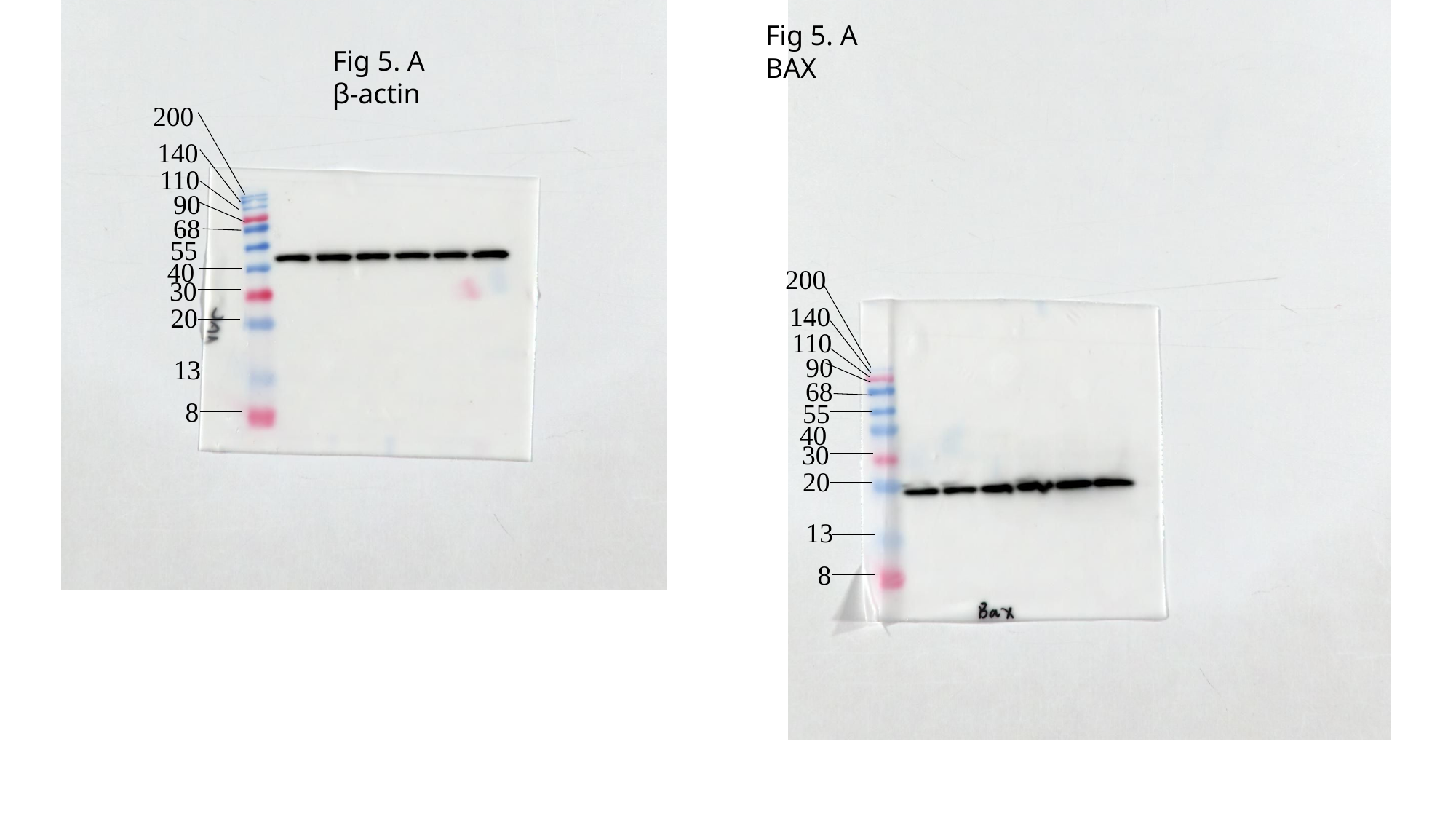

Fig 5. A
ΒAX
Fig 5. A
β-actin
200
140
110
90
68
55
40
200
30
140
20
110
90
13
68
8
55
40
30
20
13
8

## Slide 12
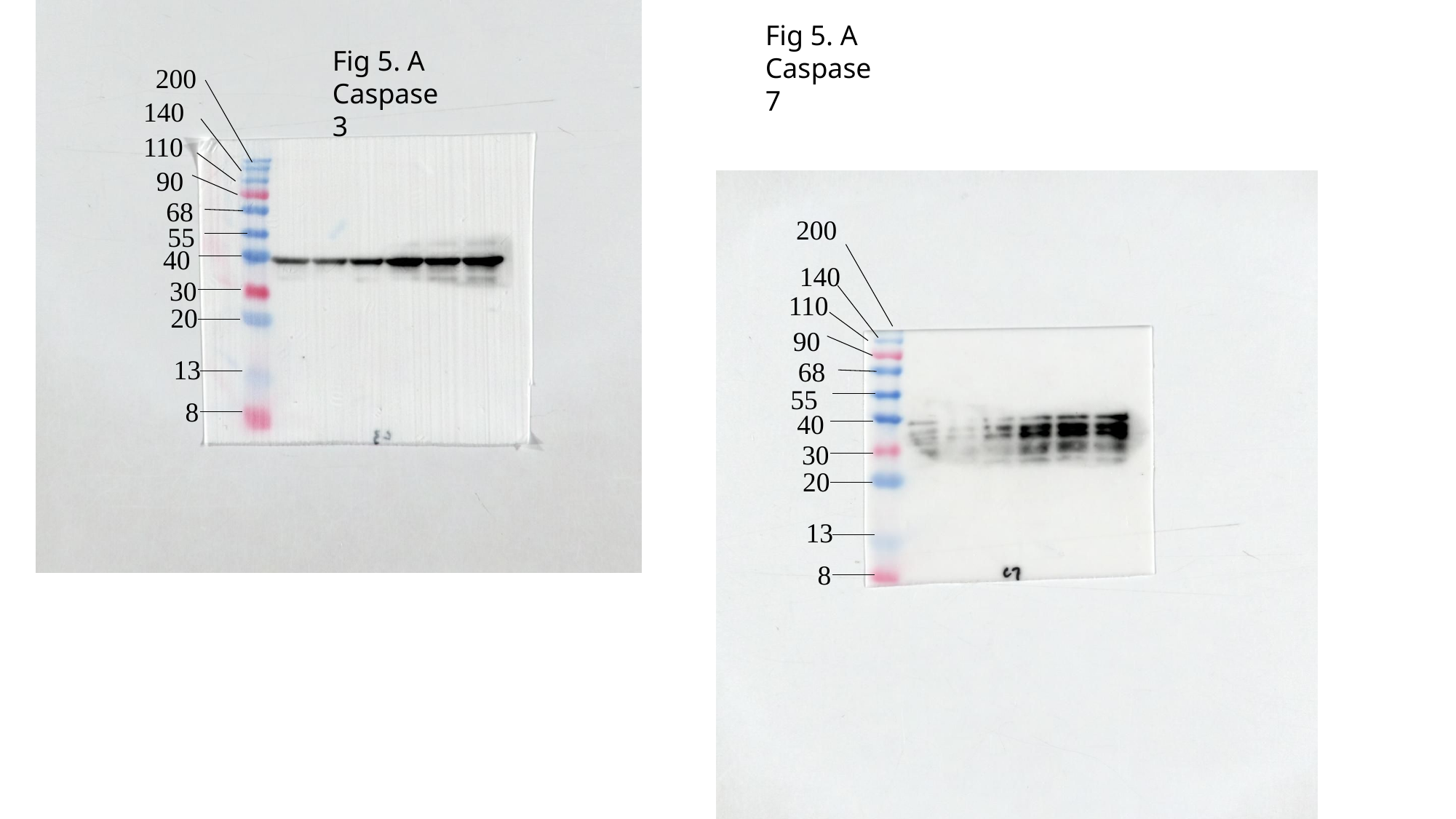

Fig 5. A
Caspase7
Fig 5. A
Caspase3
200
140
110
90
68
200
55
40
140
30
110
20
90
13
68
55
8
40
30
20
13
8

## Slide 13
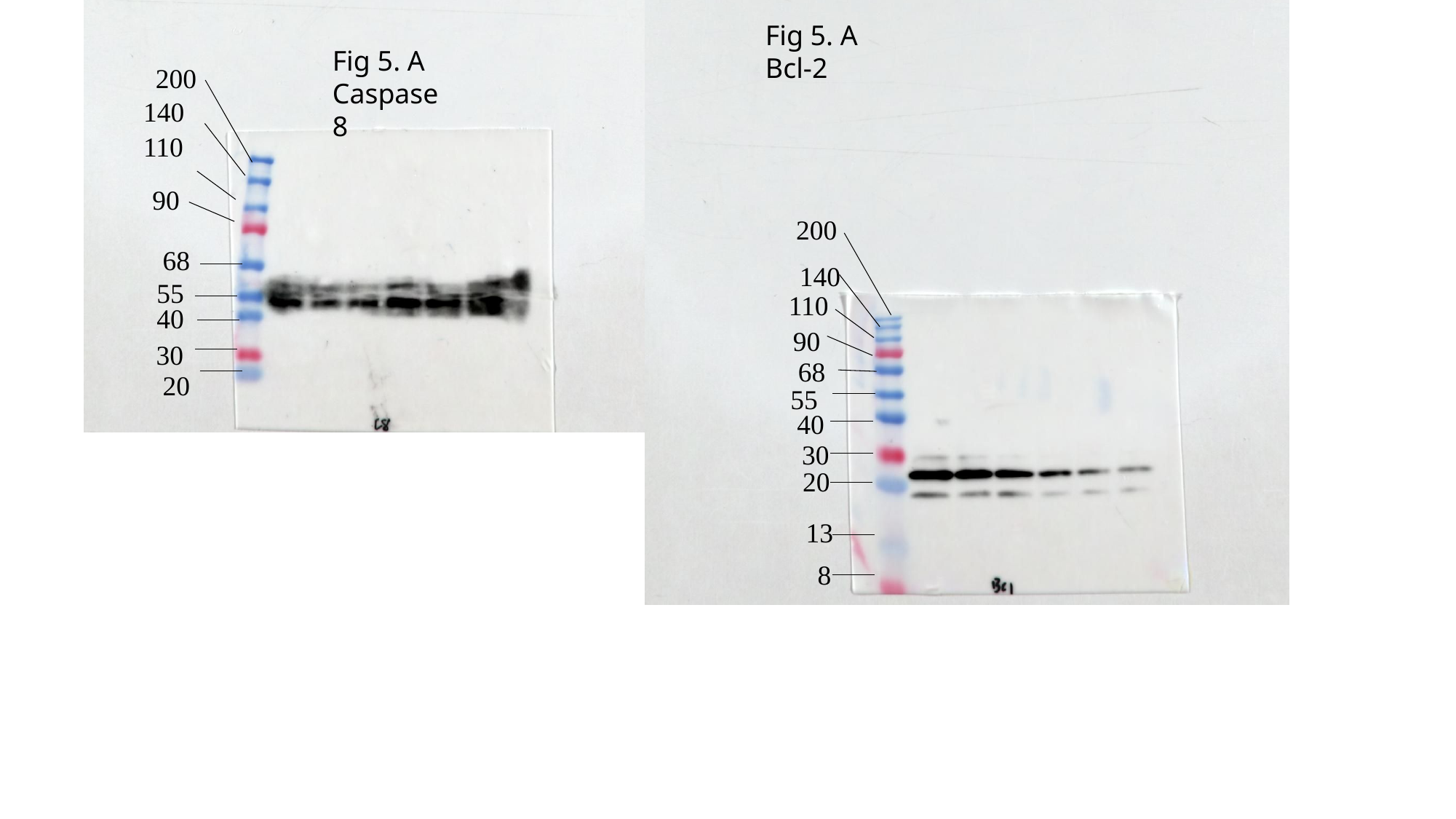

Fig 5. A
Bcl-2
Fig 5. A
Caspase8
200
140
110
90
200
68
140
55
110
40
90
30
68
20
55
40
30
20
13
8

## Slide 14
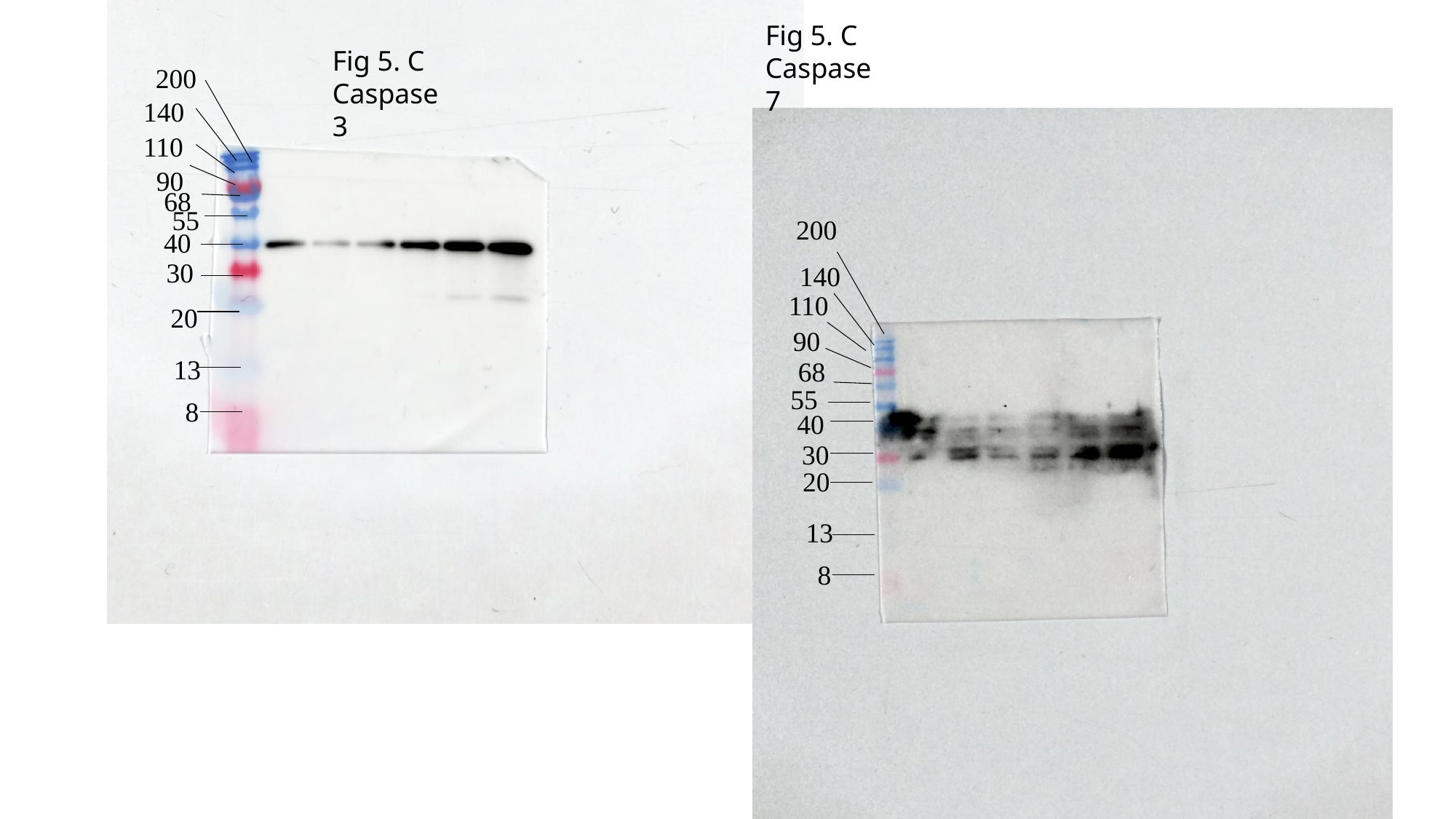

Fig 5. C
Caspase7
Fig 5. C
Caspase3
200
140
110
90
68
55
200
40
30
140
110
20
90
13
68
55
8
40
30
20
13
8

## Slide 15
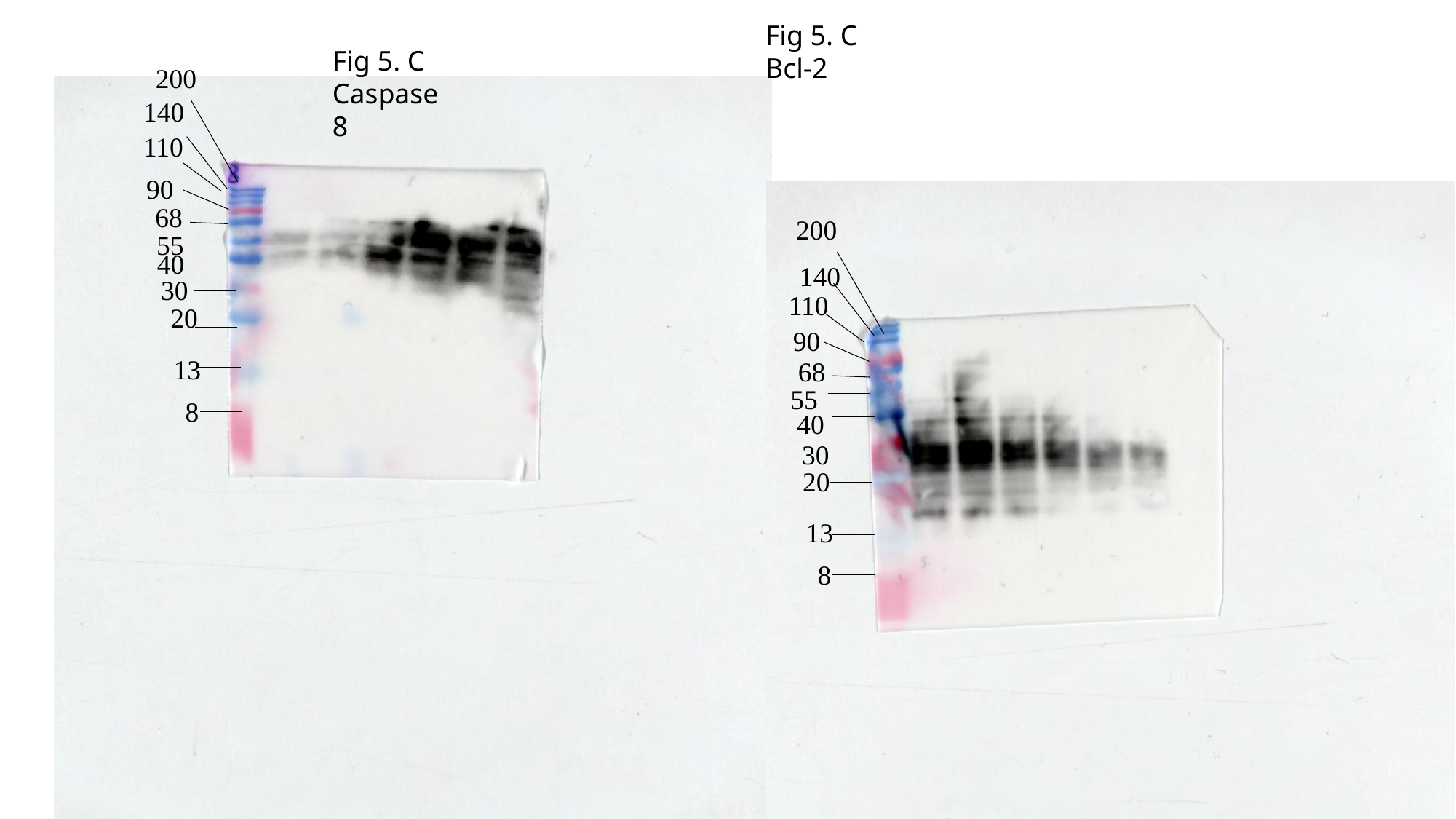

Fig 5. C
Bcl-2
Fig 5. C
Caspase8
200
140
110
90
68
200
55
40
140
30
110
20
90
13
68
55
8
40
30
20
13
8

## Slide 16
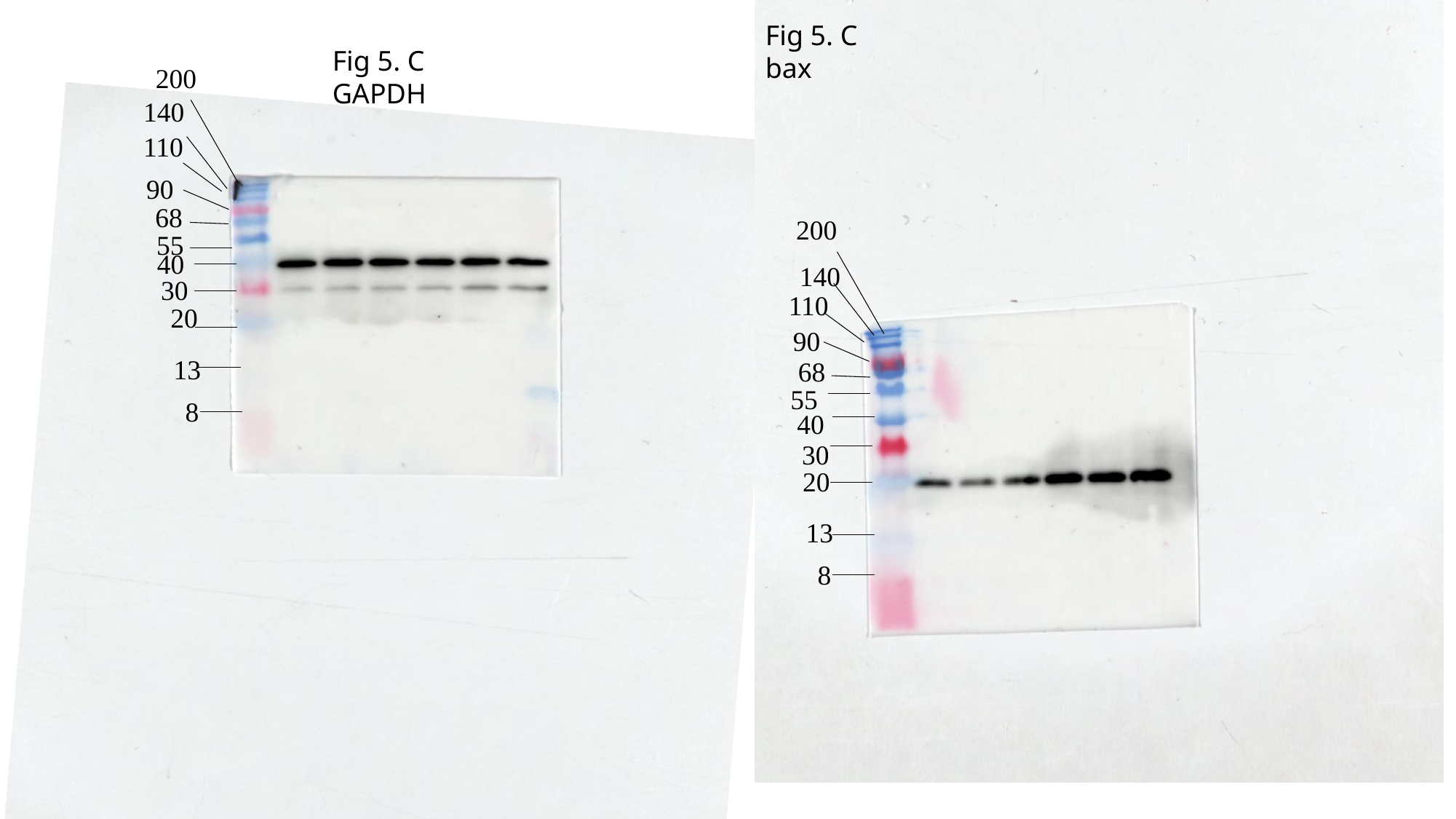

Fig 5. C
bax
Fig 5. C
GAPDH
200
140
110
90
68
200
55
40
140
30
110
20
90
13
68
55
8
40
30
20
13
8

## Slide 17
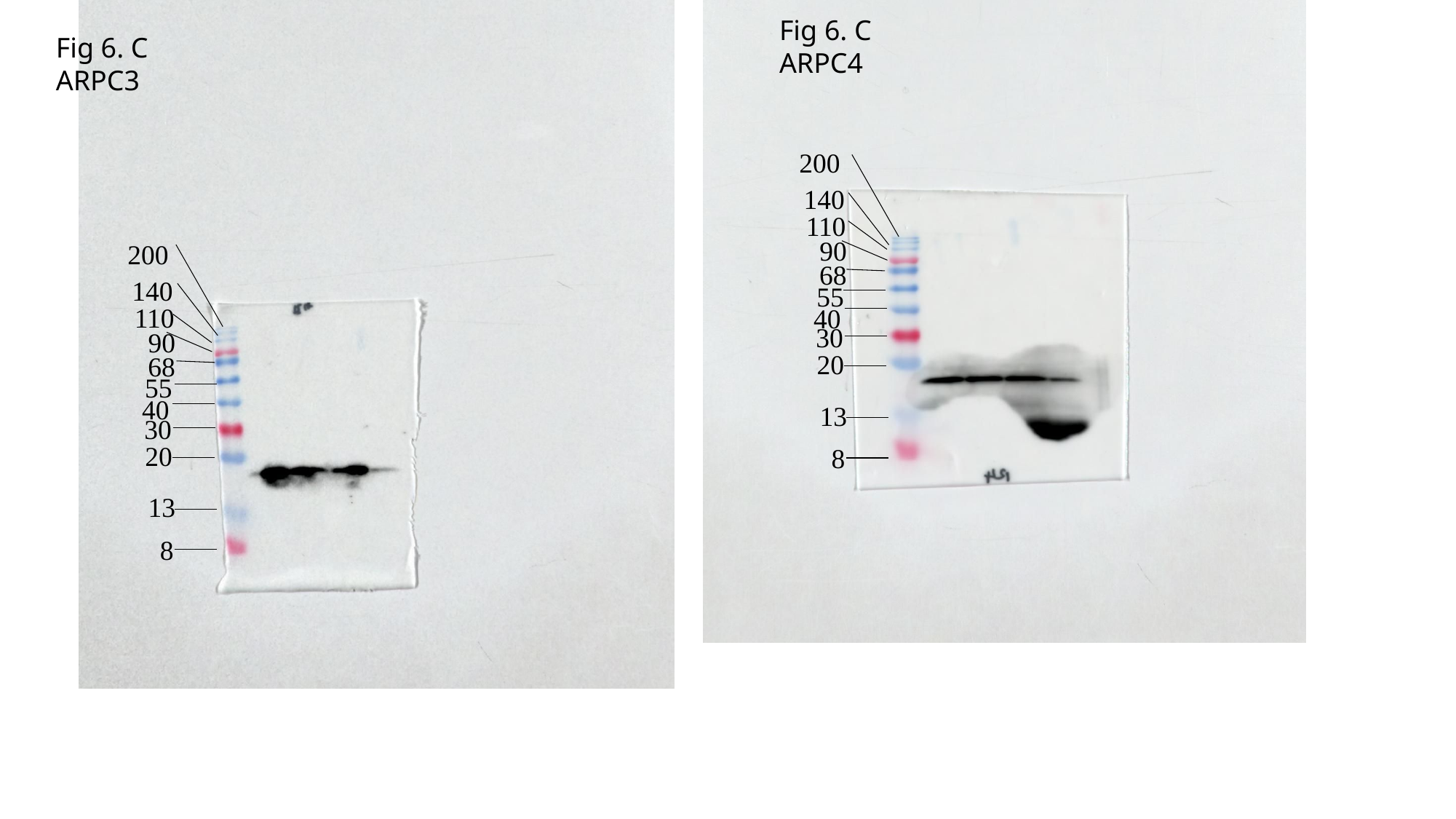

Fig 6. C
ARPC4
Fig 6. C
ARPC3
200
140
110
90
200
68
140
55
110
40
30
90
20
68
55
40
13
30
20
8
13
8

## Slide 18
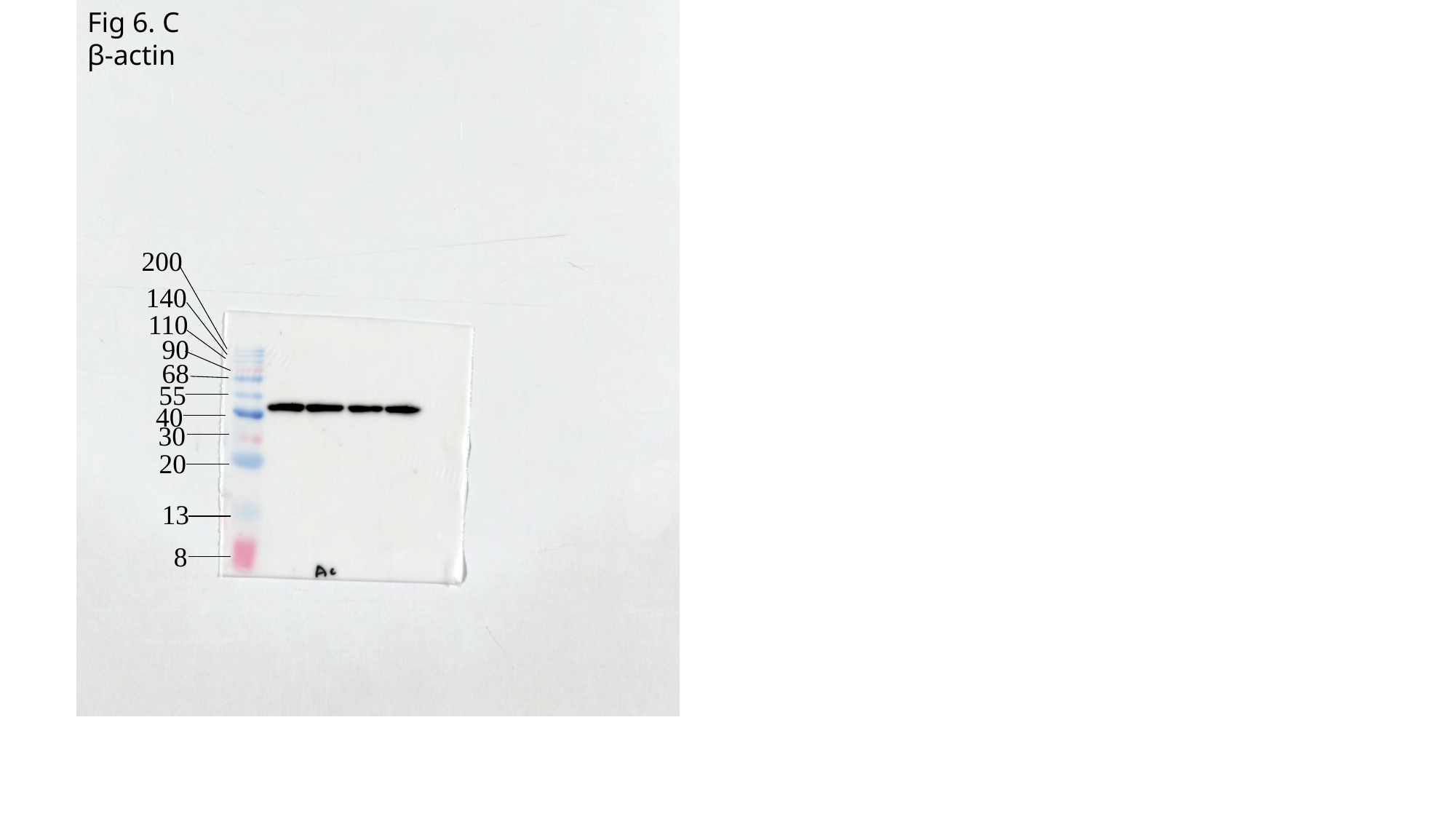

Fig 6. C
β-actin
200
140
110
90
68
55
40
30
20
13
8

## Slide 19
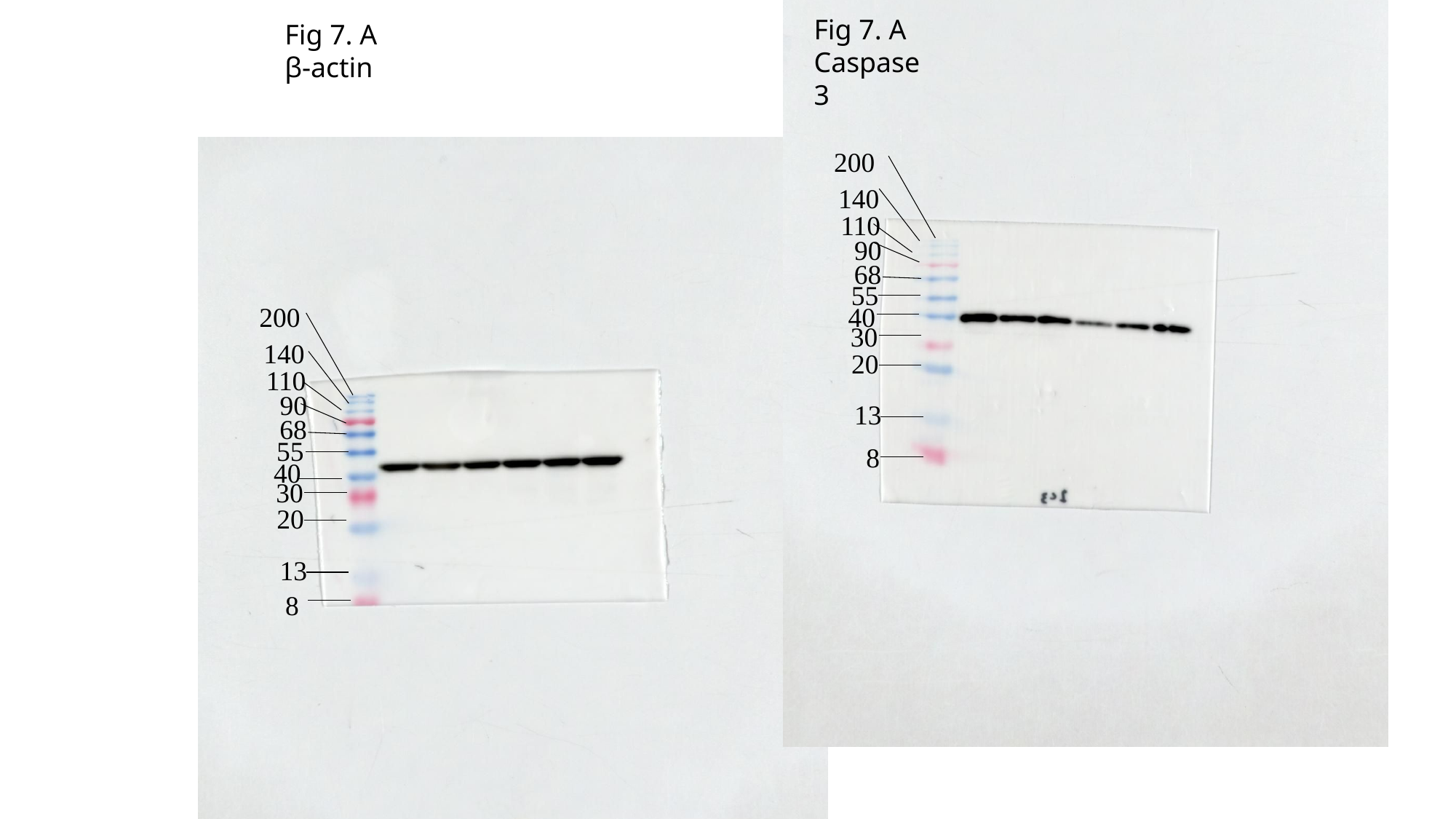

Fig 7. A
Caspase3
Fig 7. A
β-actin
200
140
110
90
68
55
200
40
30
140
20
110
90
13
68
55
8
40
30
20
13
8

## Slide 20
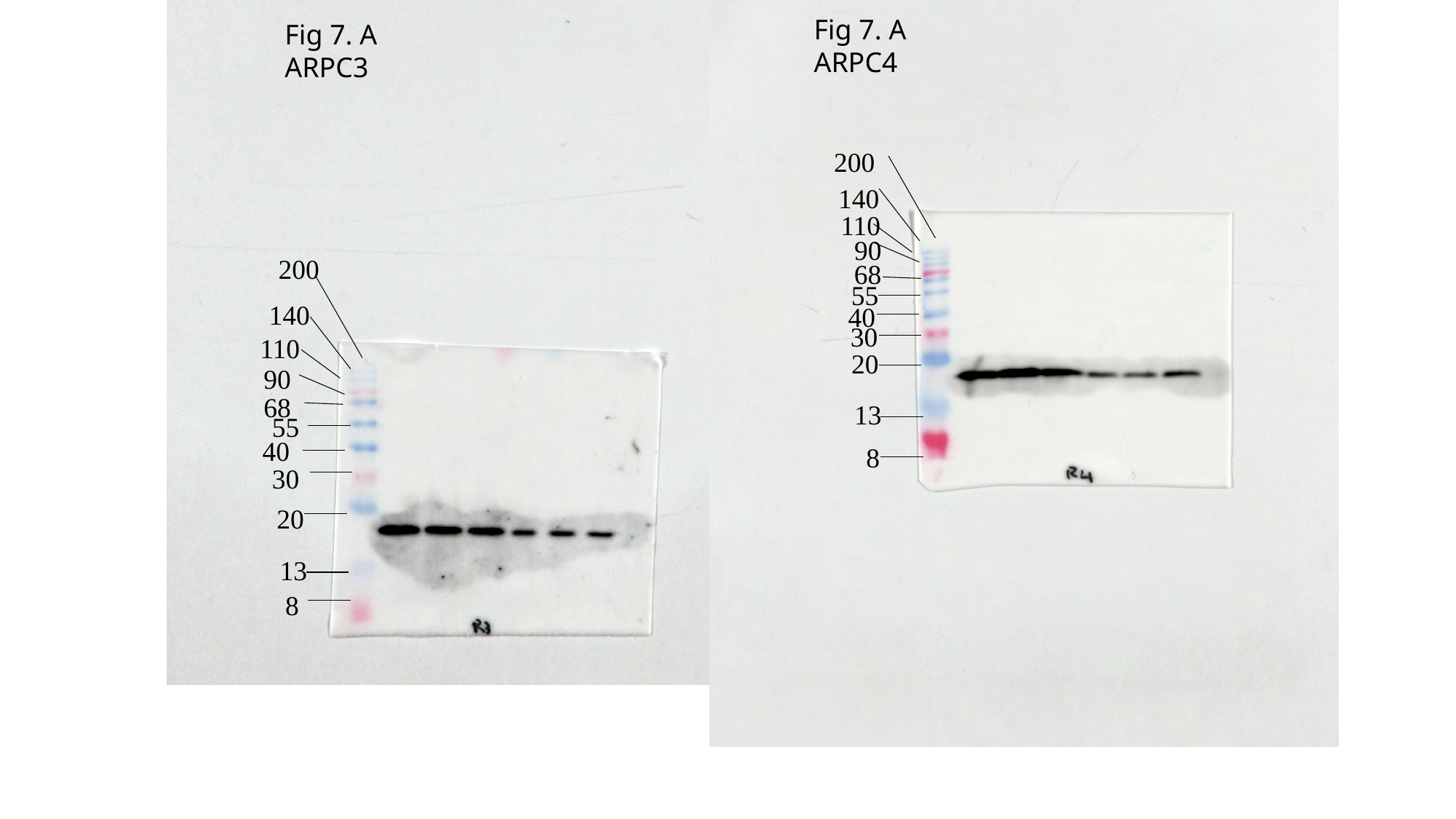

Fig 7. A
ARPC4
Fig 7. A
ARPC3
200
140
110
90
200
68
55
140
40
30
110
20
90
68
13
55
40
8
30
20
13
8

## Slide 21
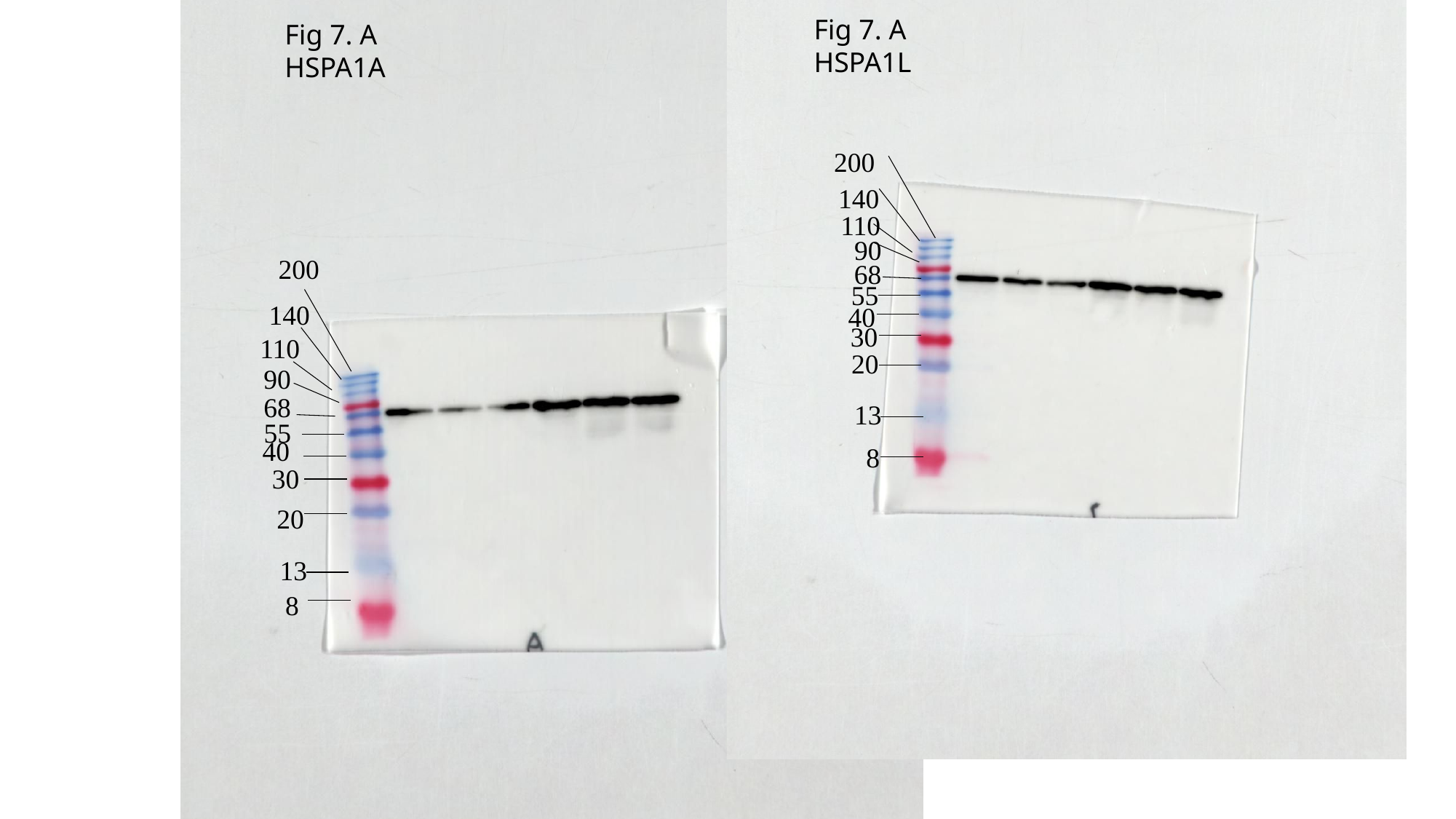

Fig 7. A
HSPA1L
Fig 7. A
HSPA1A
200
140
110
90
200
68
55
140
40
30
110
20
90
68
13
55
40
8
30
20
13
8

## Slide 22
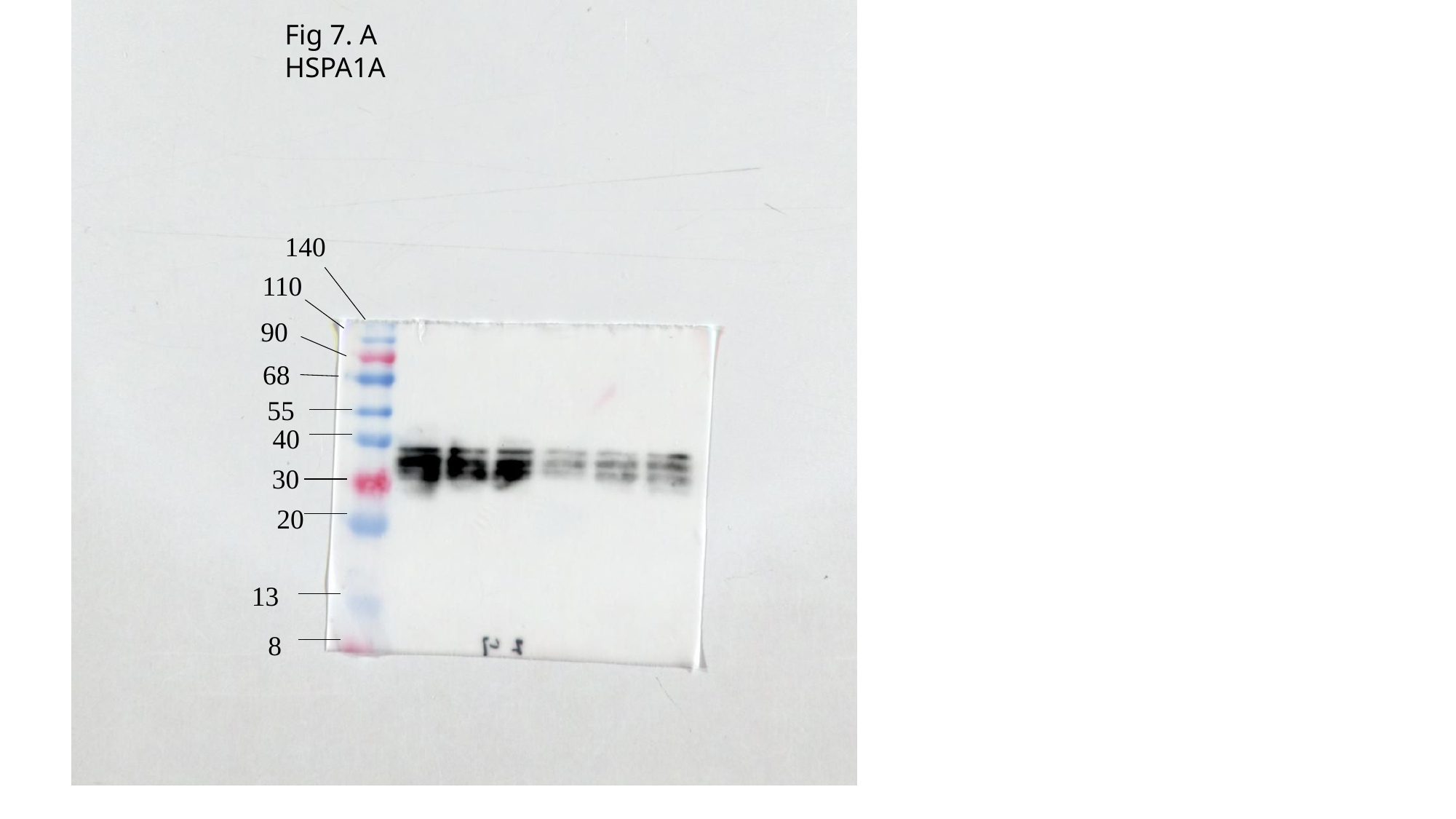

Fig 7. A
HSPA1A
140
110
90
68
55
40
30
20
13
8

## Slide 23
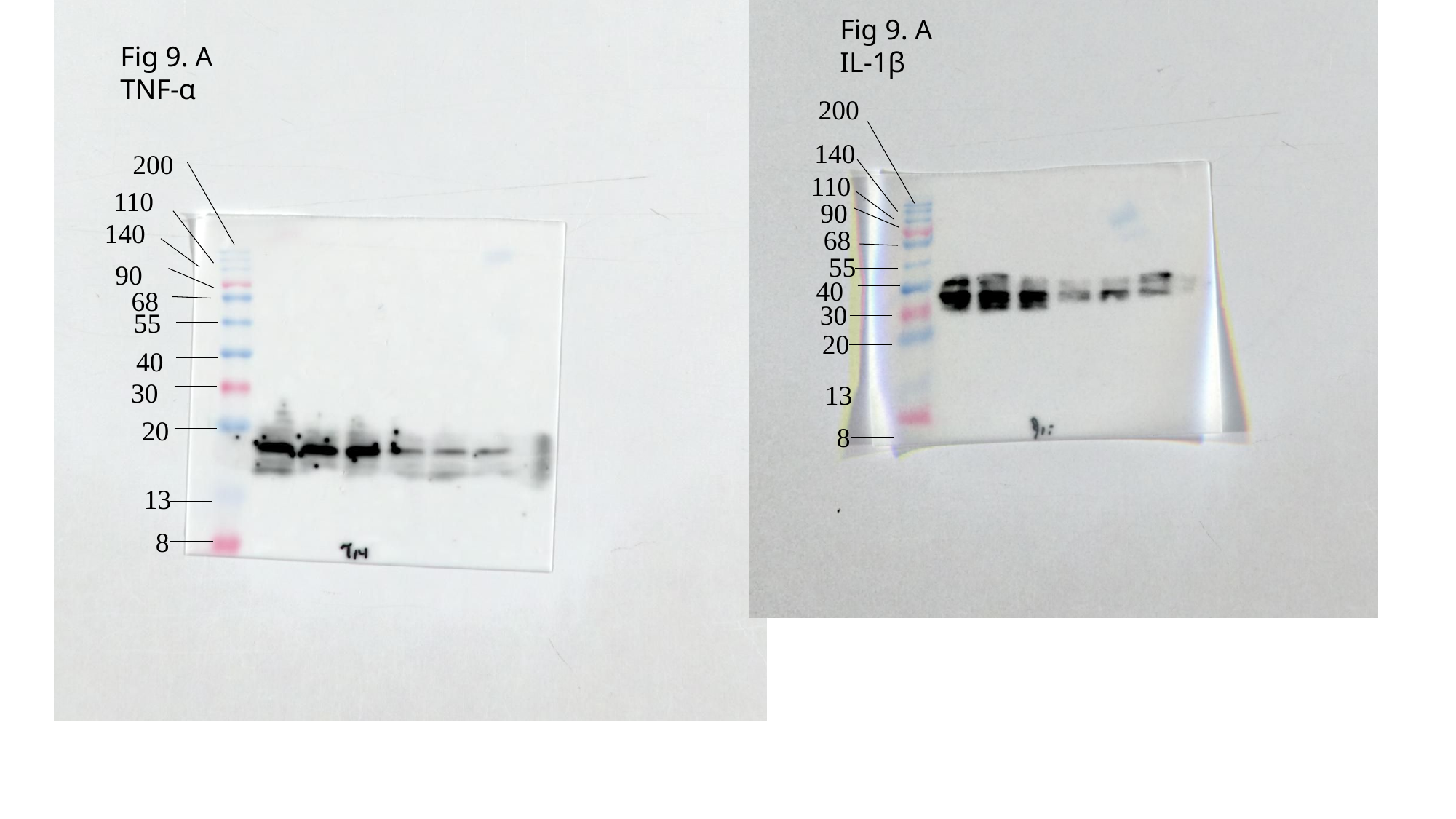

Fig 9. A
IL-1β
Fig 9. A
TNF-α
200
140
200
110
110
90
140
68
55
90
40
68
30
55
20
40
30
13
20
8
13
8

## Slide 24
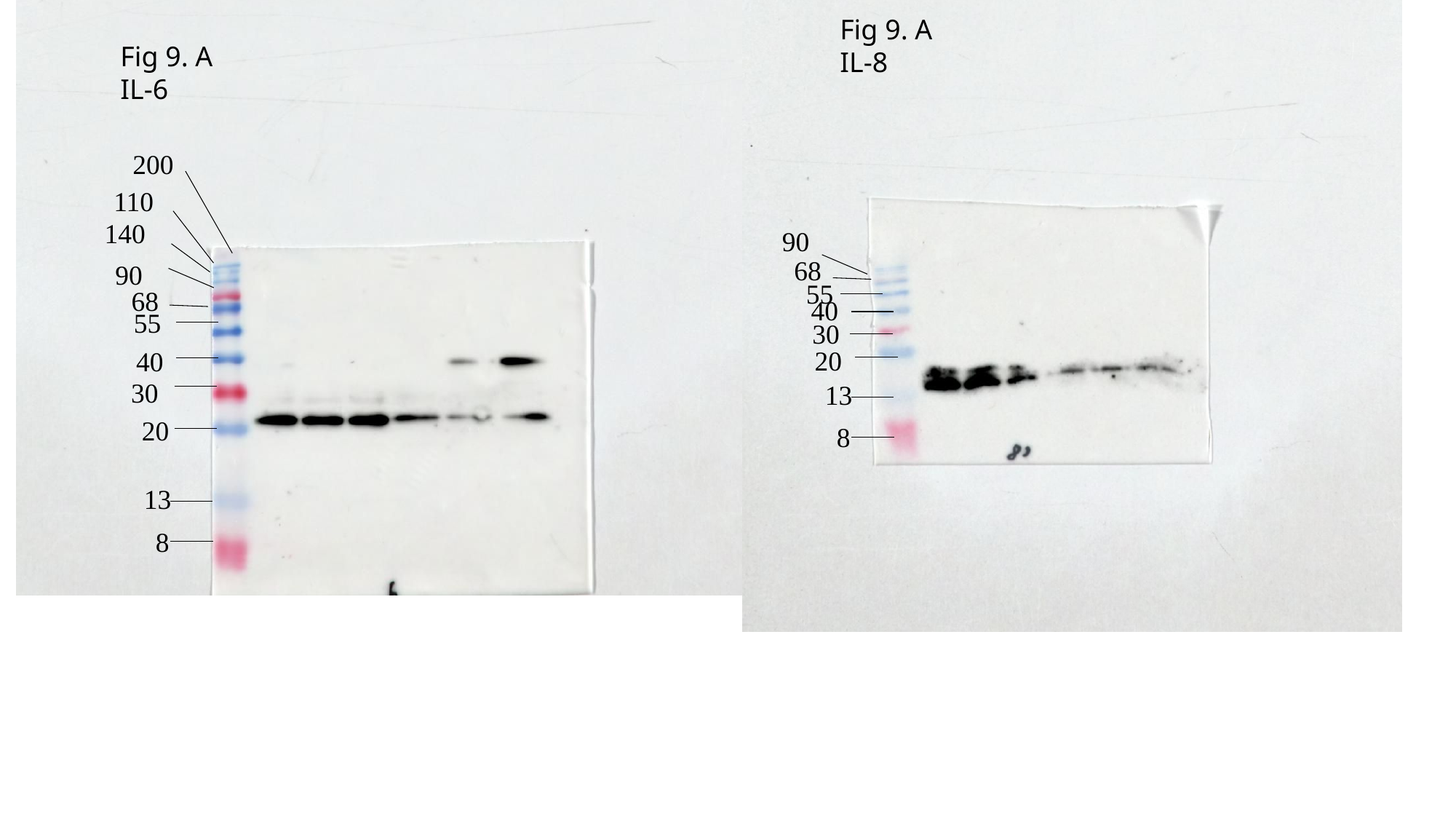

Fig 9. A
IL-8
Fig 9. A
IL-6
200
110
140
90
68
90
55
68
40
55
30
20
40
30
13
20
8
13
8

## Slide 25
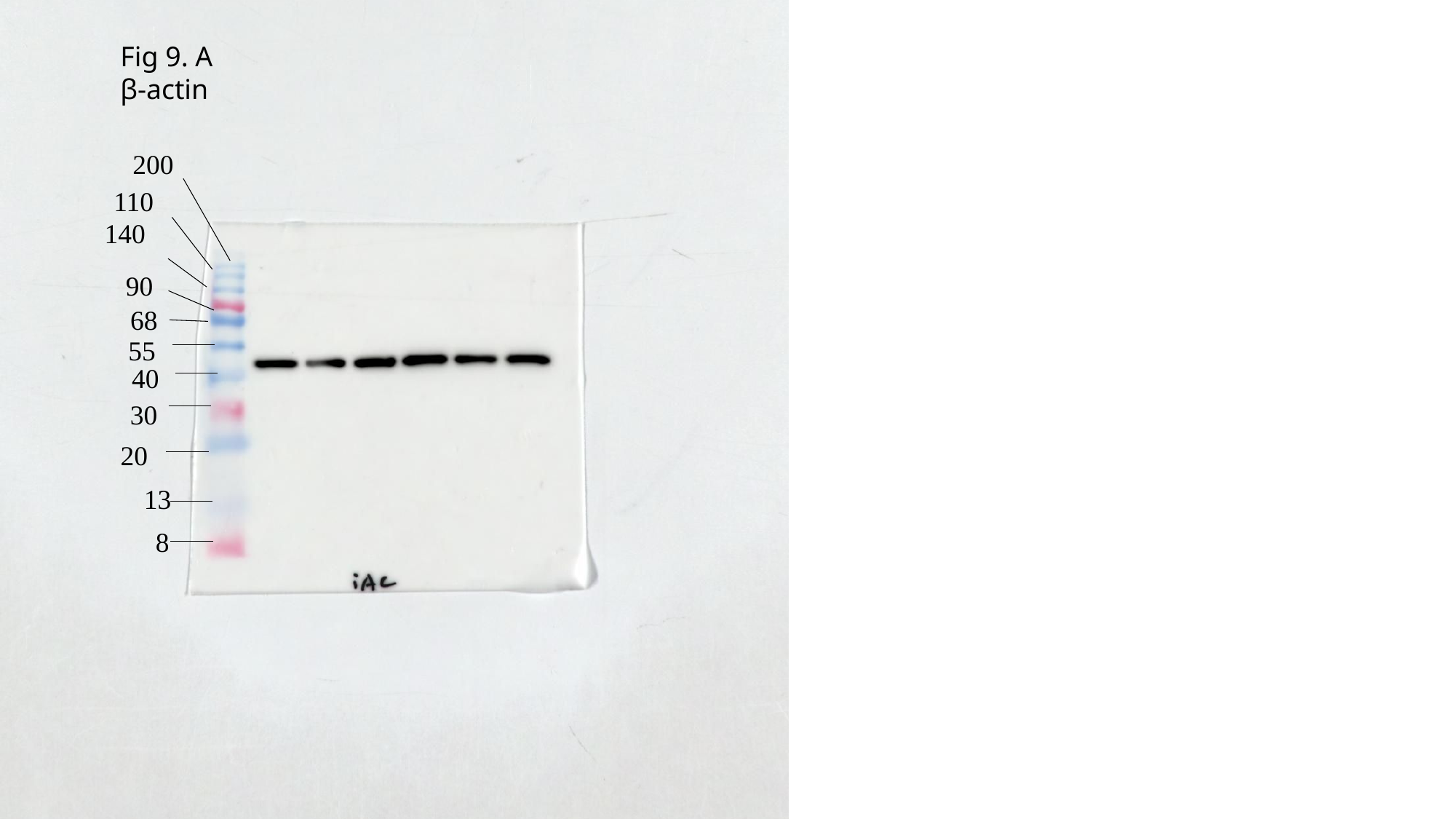

Fig 9. A
β-actin
200
110
140
90
68
55
40
30
20
13
8
