# Supplementary figures and images for: Targeted Regulation of HSP70 by the ARP2/3 Complex in Mammary Epithelial Cells and Its Impact on Host Cell Apoptosis
Source: Biomolecules. 2025 Apr 7;15(4):538. doi: 10.3390/biom15040538 (PMC12025207; doi:10.3390/biom15040538)

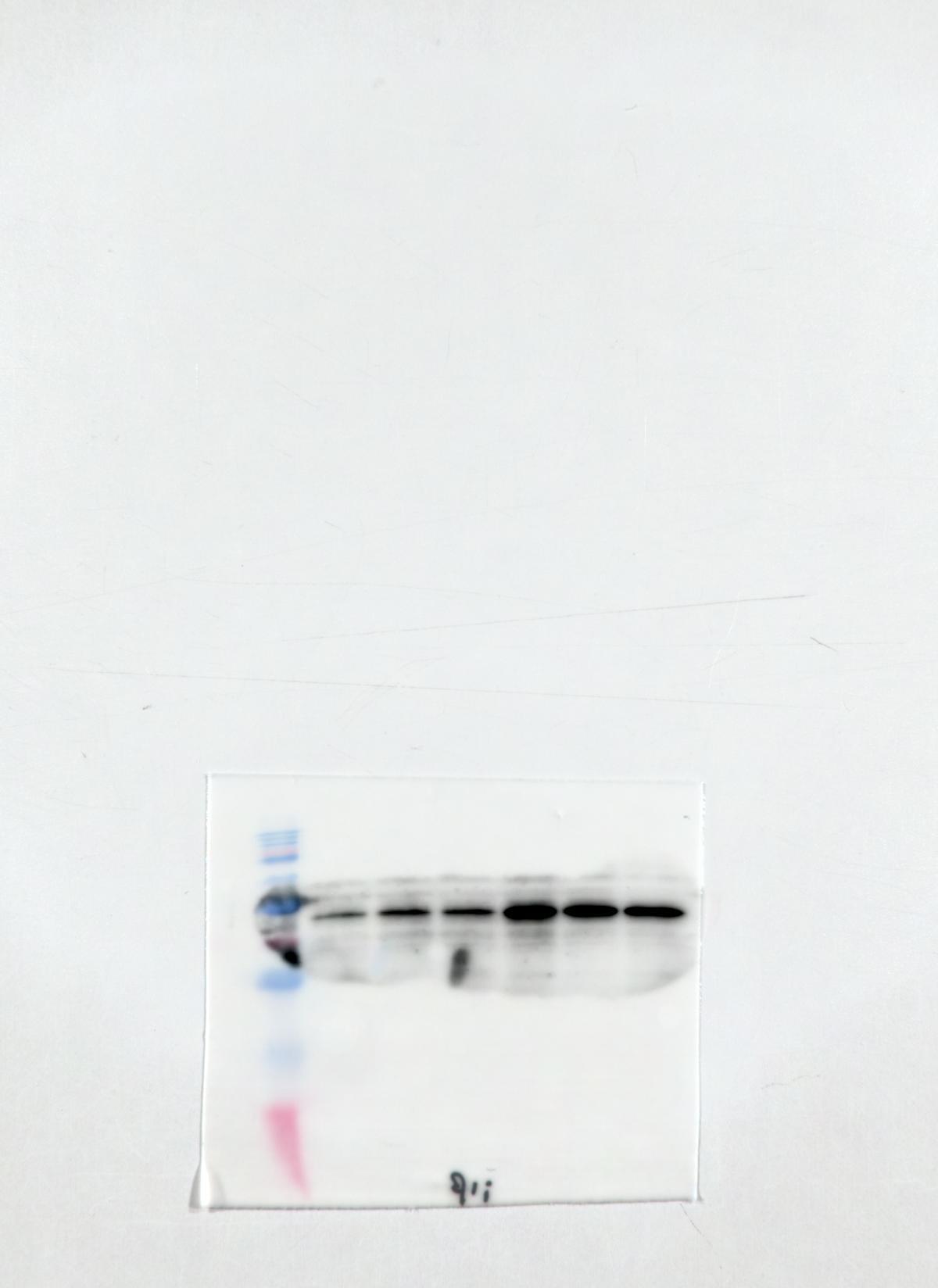

Supplement: Supplementary file 1 [file biomolecules-15-00538-s001.zip › original image/Fig1C. IL-1a┬.jpg]

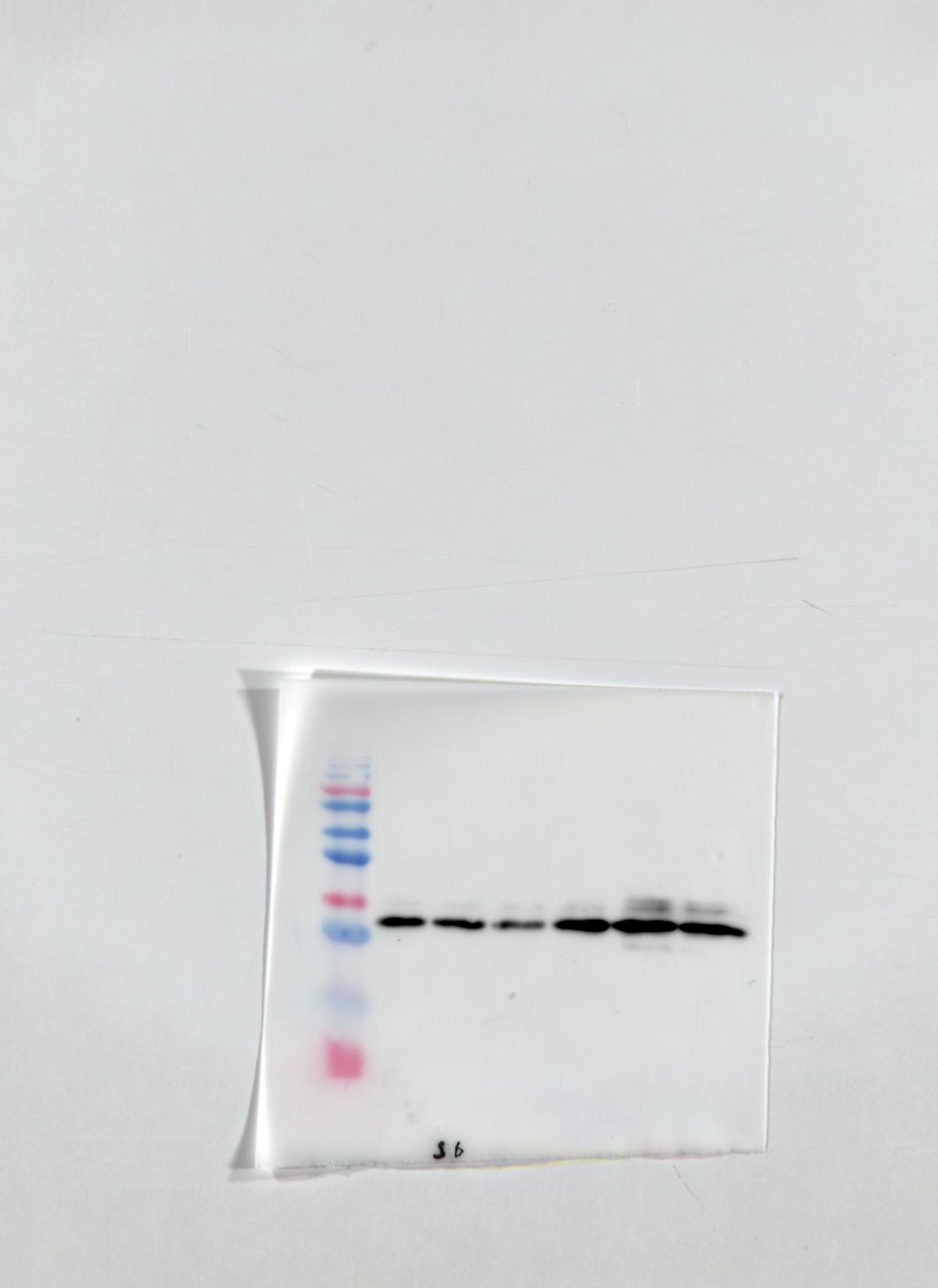

Supplement: Supplementary file 1 [file biomolecules-15-00538-s001.zip › original image/Fig1C. IL-6.jpg]

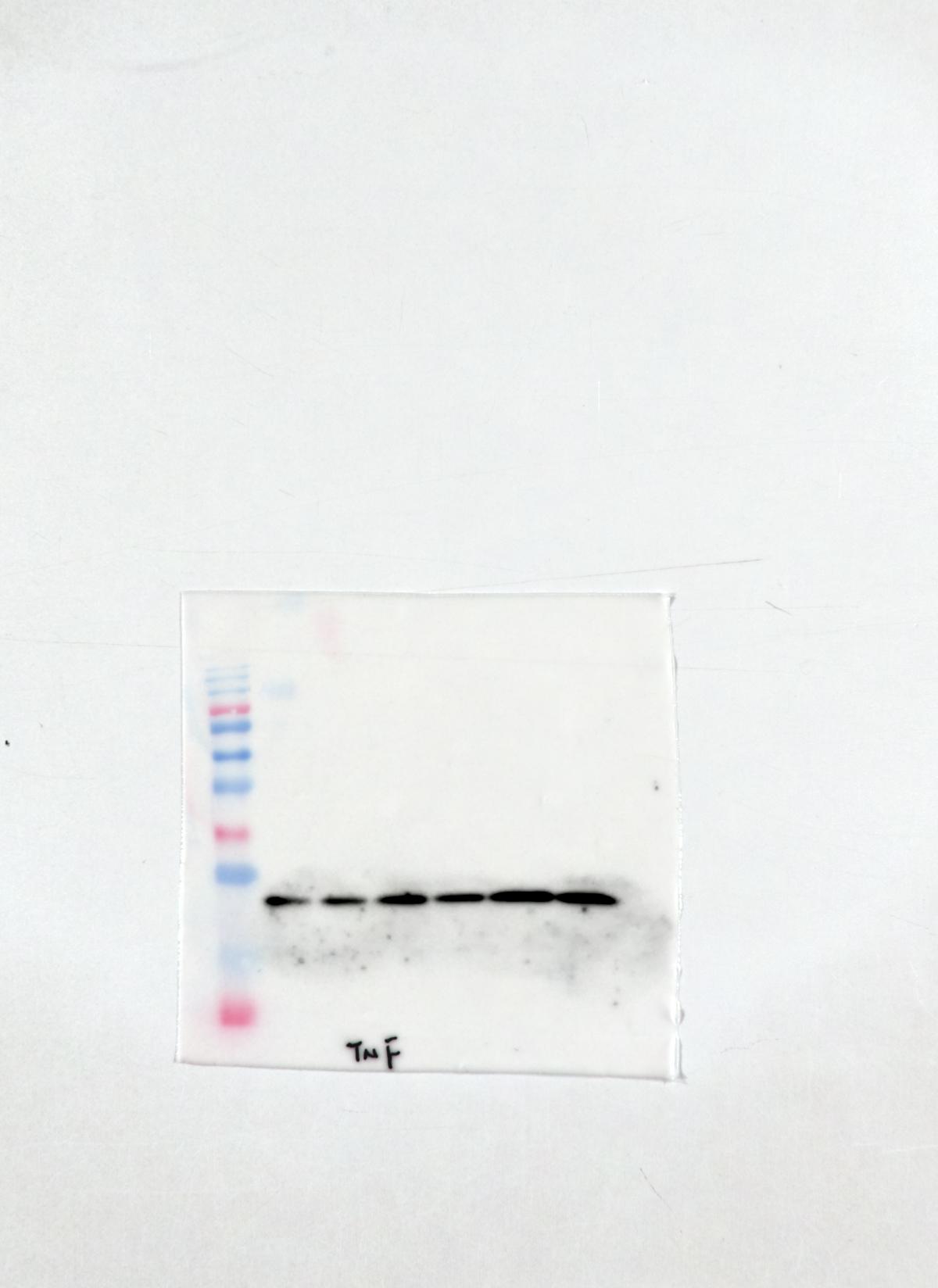

Supplement: Supplementary file 1 [file biomolecules-15-00538-s001.zip › original image/Fig1C. TNF-a┴.jpg]

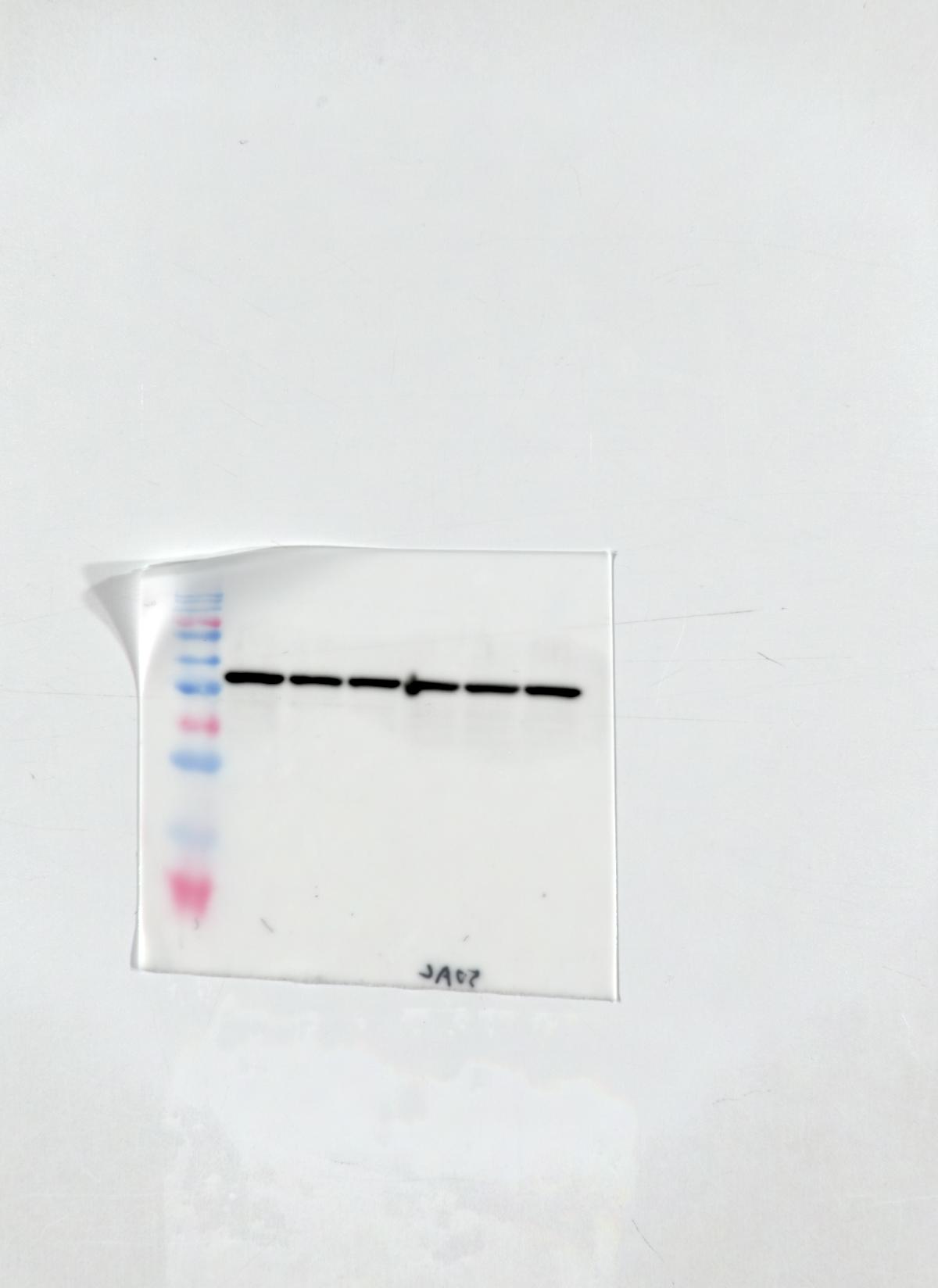

Supplement: Supplementary file 1 [file biomolecules-15-00538-s001.zip › original image/Fig1C. a┬-actin.jpg]

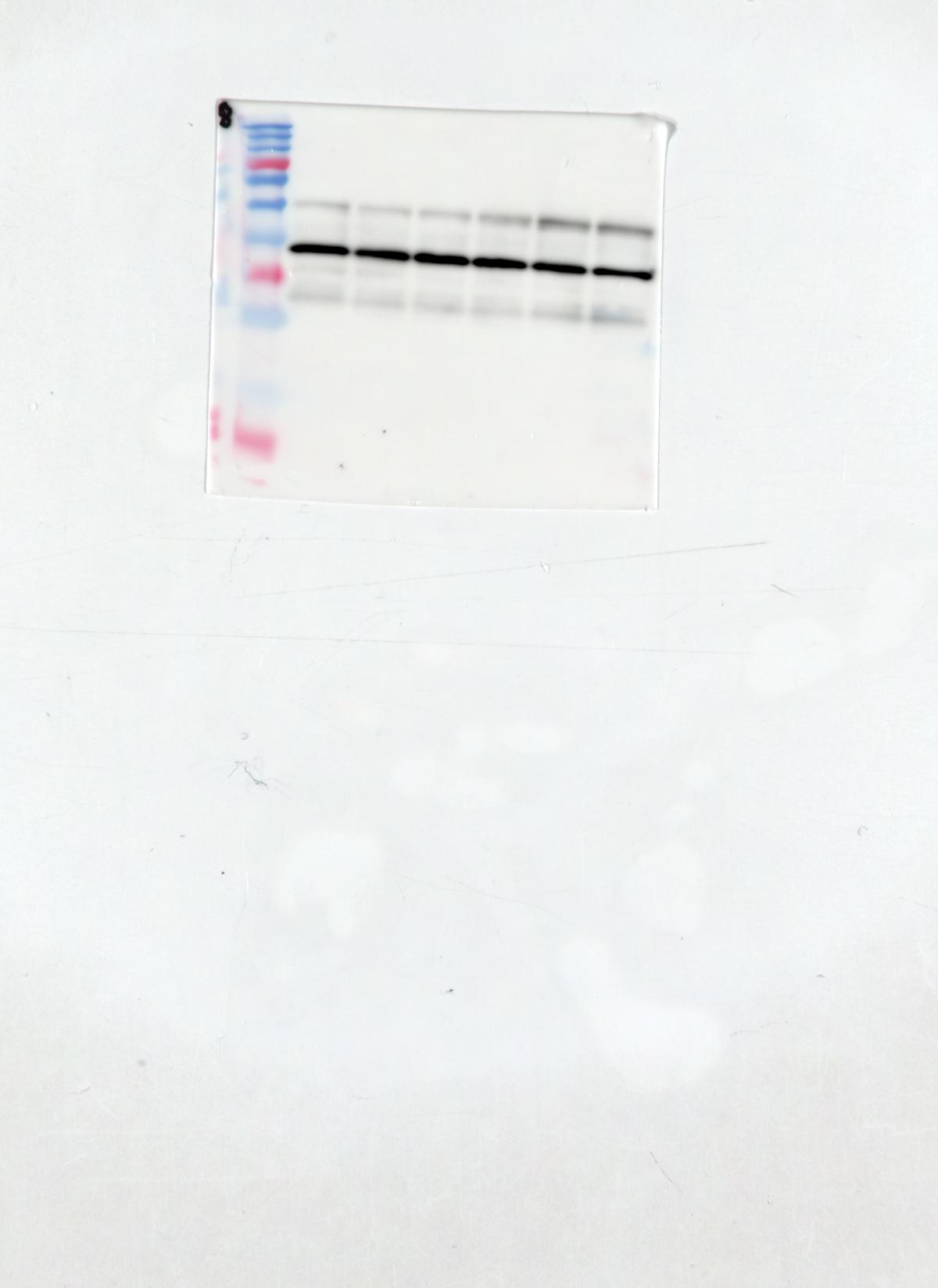

Supplement: Supplementary file 1 [file biomolecules-15-00538-s001.zip › original image/Fig2E.GAPDH.jpg]

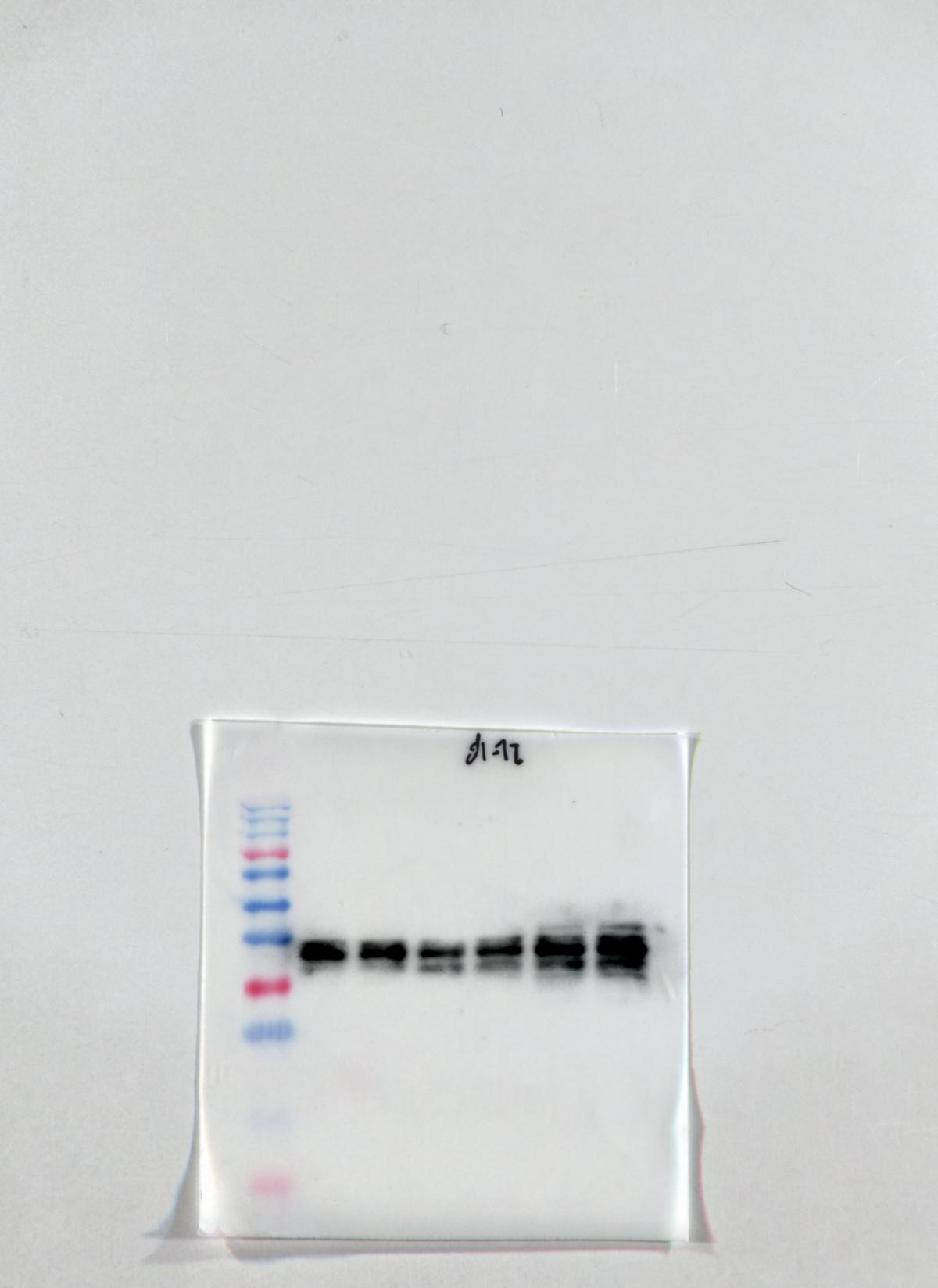

Supplement: Supplementary file 1 [file biomolecules-15-00538-s001.zip › original image/Fig2E.IL-1a┬.jpg]

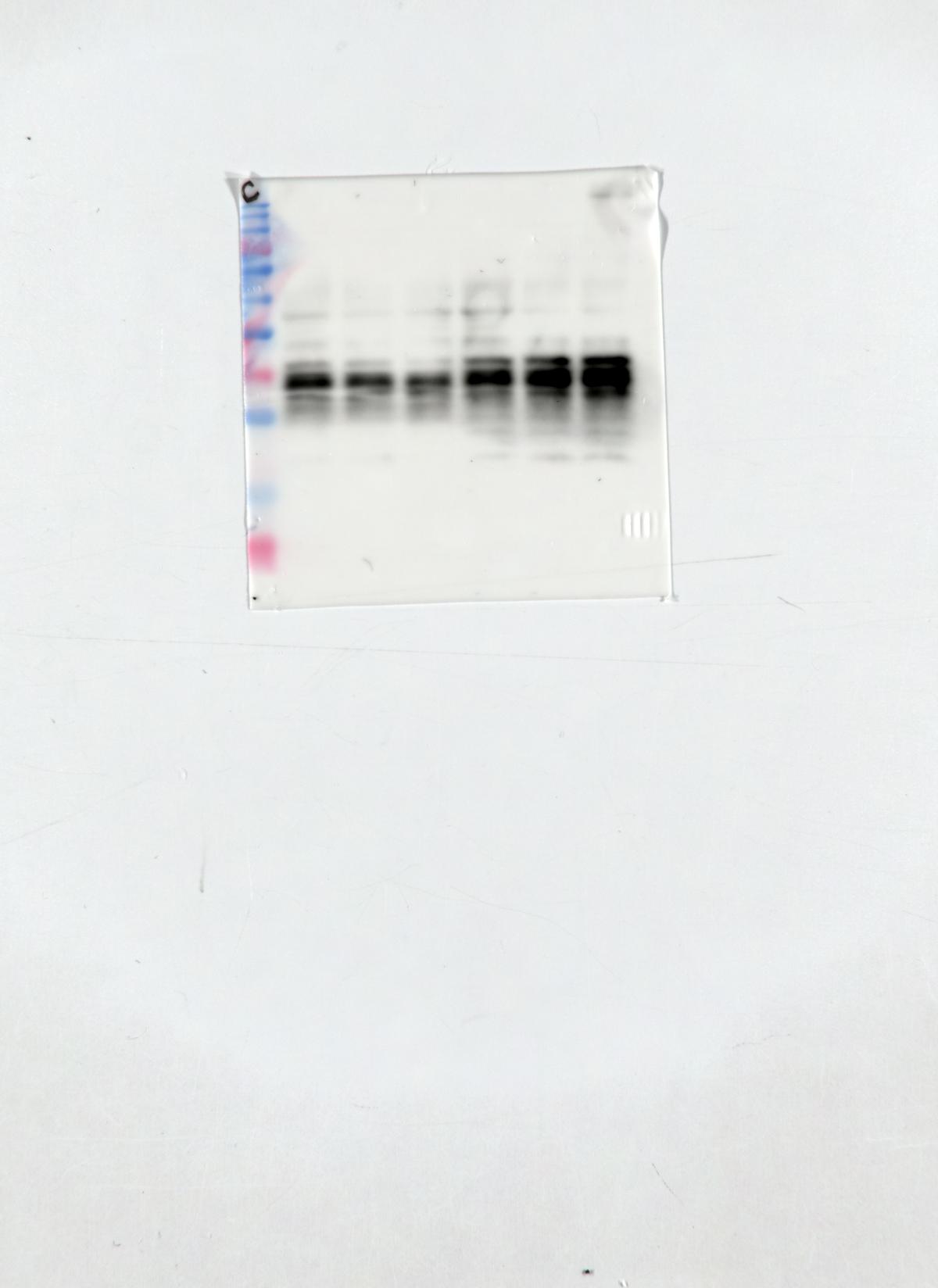

Supplement: Supplementary file 1 [file biomolecules-15-00538-s001.zip › original image/Fig2E.IL-6.jpg]

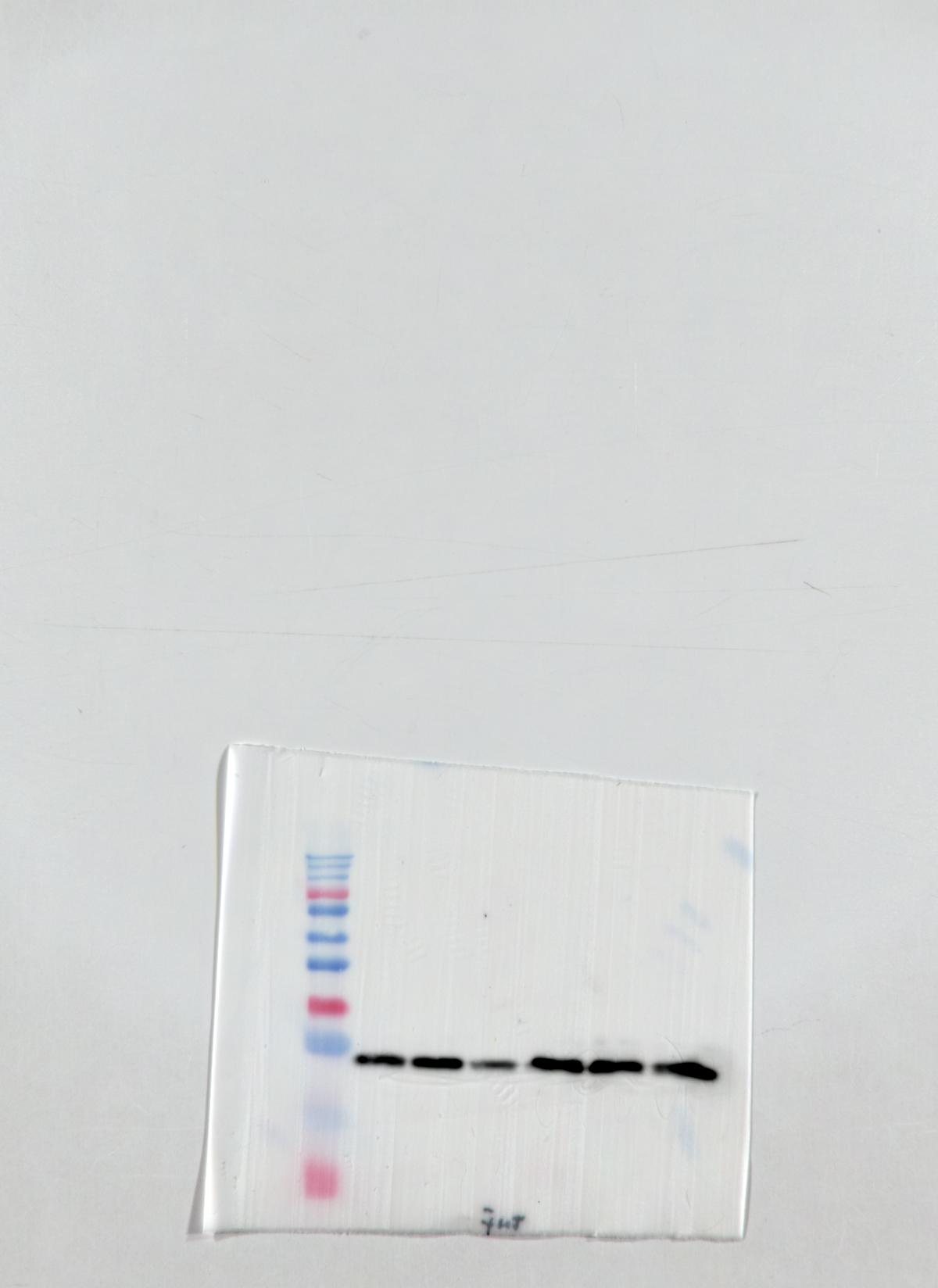

Supplement: Supplementary file 1 [file biomolecules-15-00538-s001.zip › original image/Fig2E.TNF-a┴.jpg]

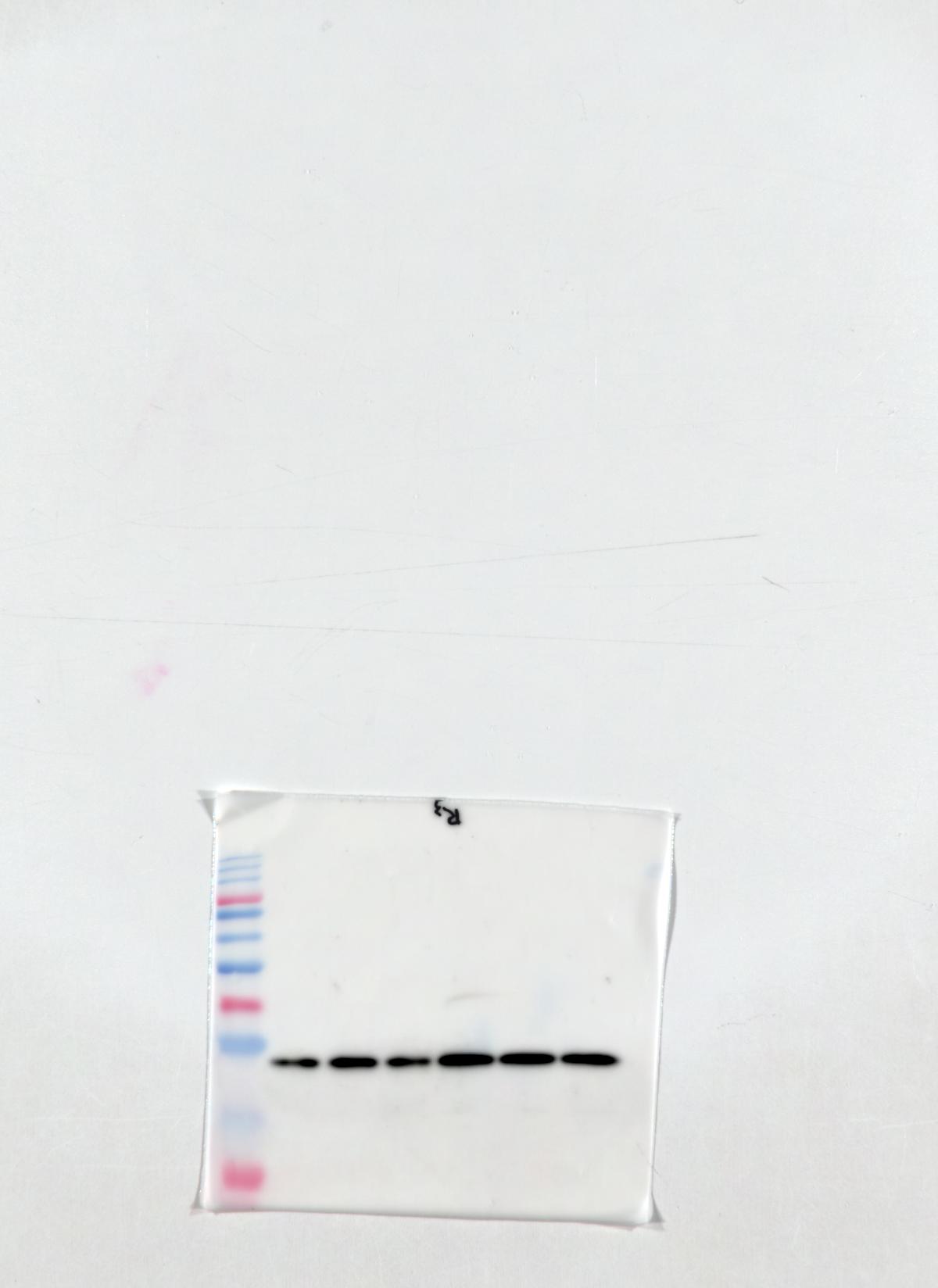

Supplement: Supplementary file 1 [file biomolecules-15-00538-s001.zip › original image/Fig4B.ARPC3.jpg]

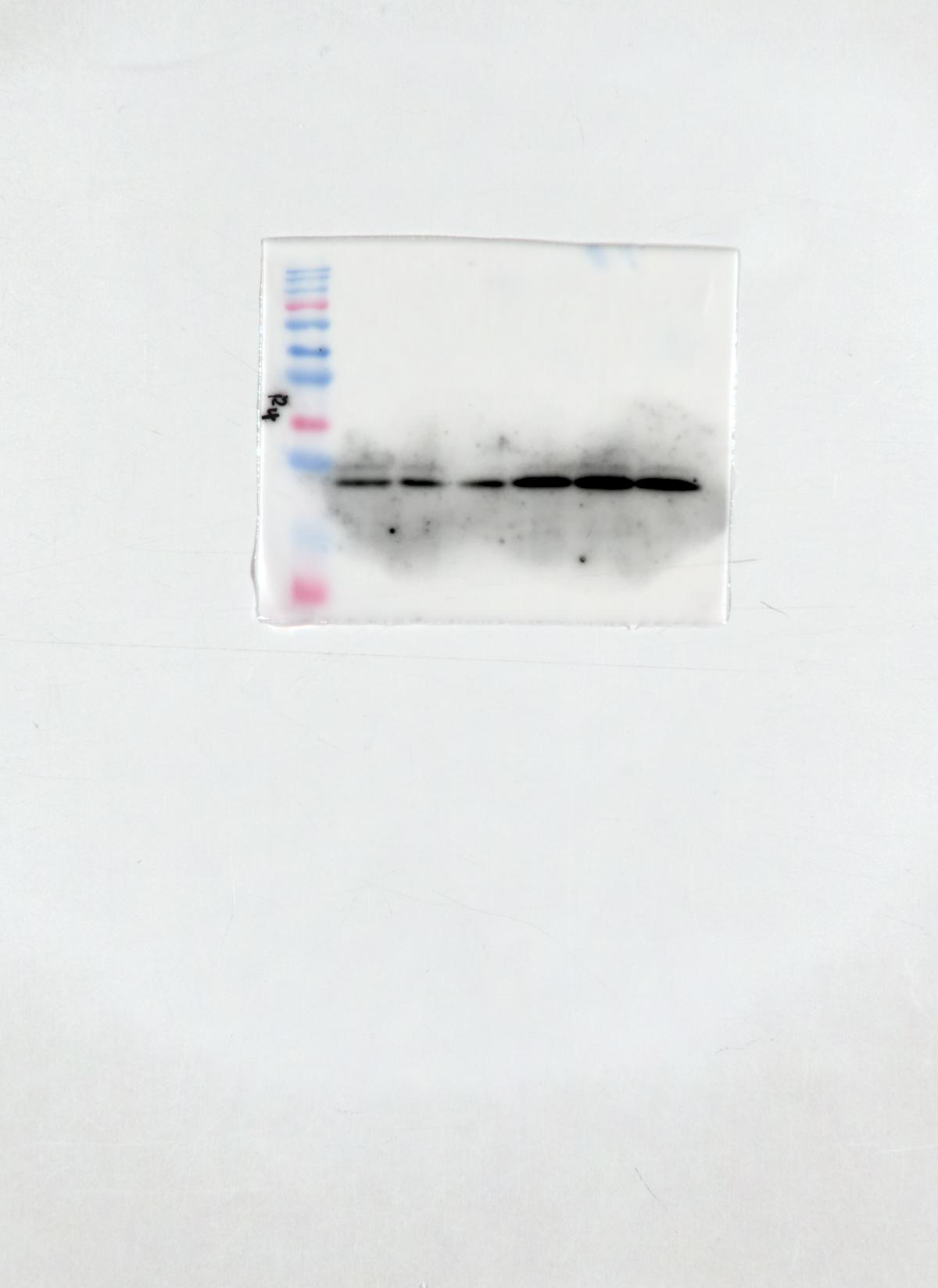

Supplement: Supplementary file 1 [file biomolecules-15-00538-s001.zip › original image/Fig4B.ARPC4.jpg]

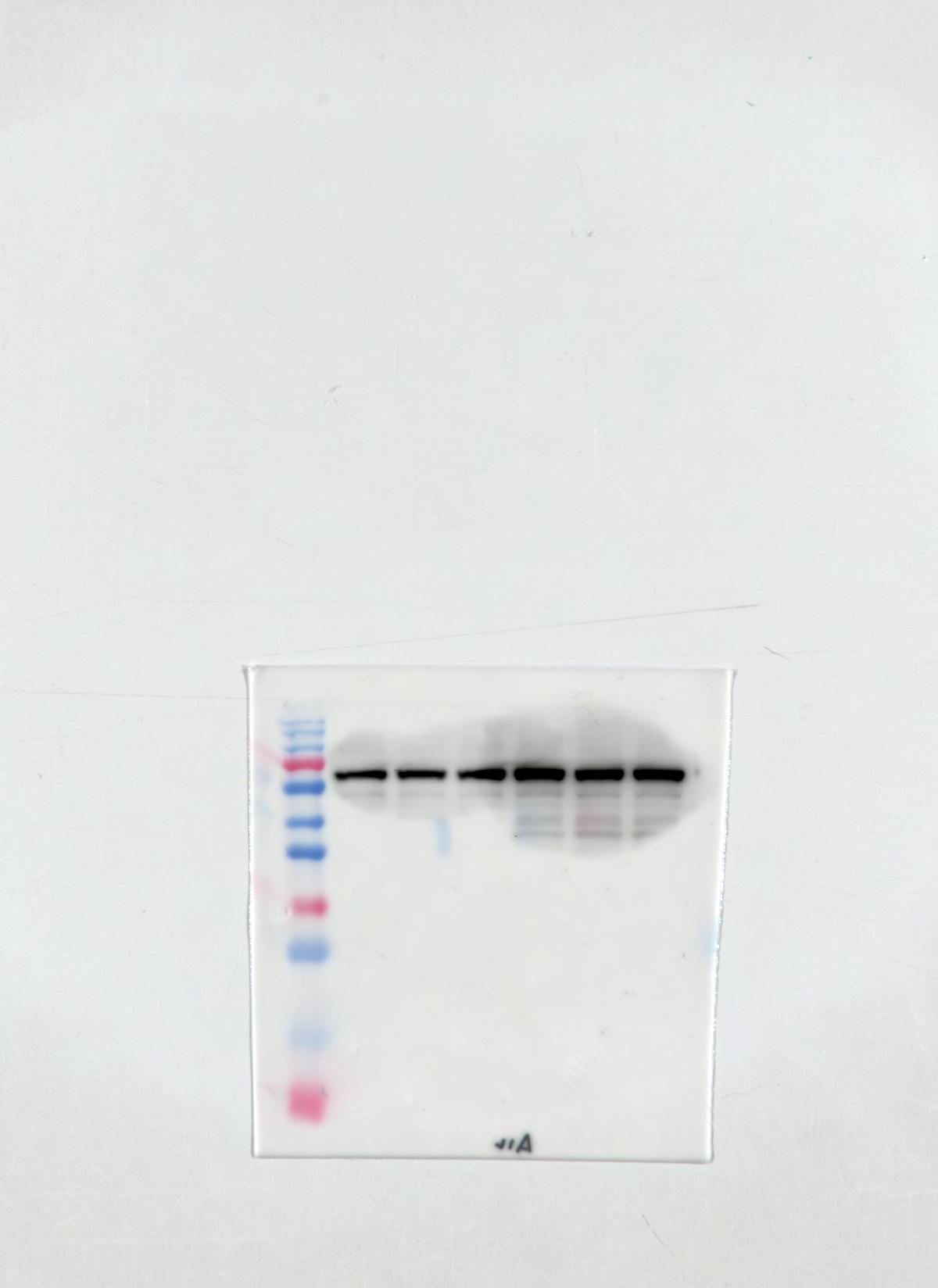

Supplement: Supplementary file 1 [file biomolecules-15-00538-s001.zip › original image/Fig4B.HSPA1A.jpg]

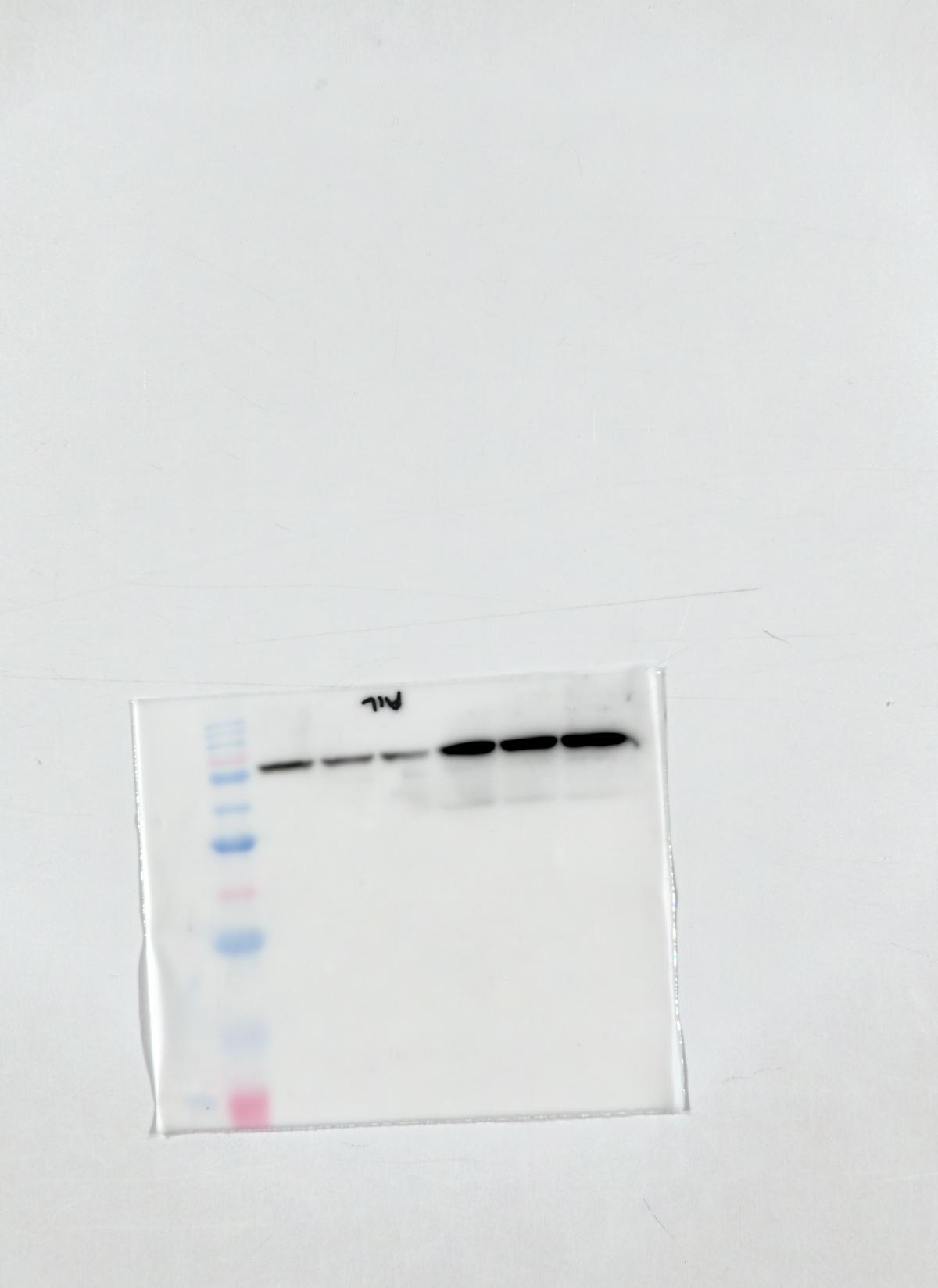

Supplement: Supplementary file 1 [file biomolecules-15-00538-s001.zip › original image/Fig4B.HSPA1L.jpg]

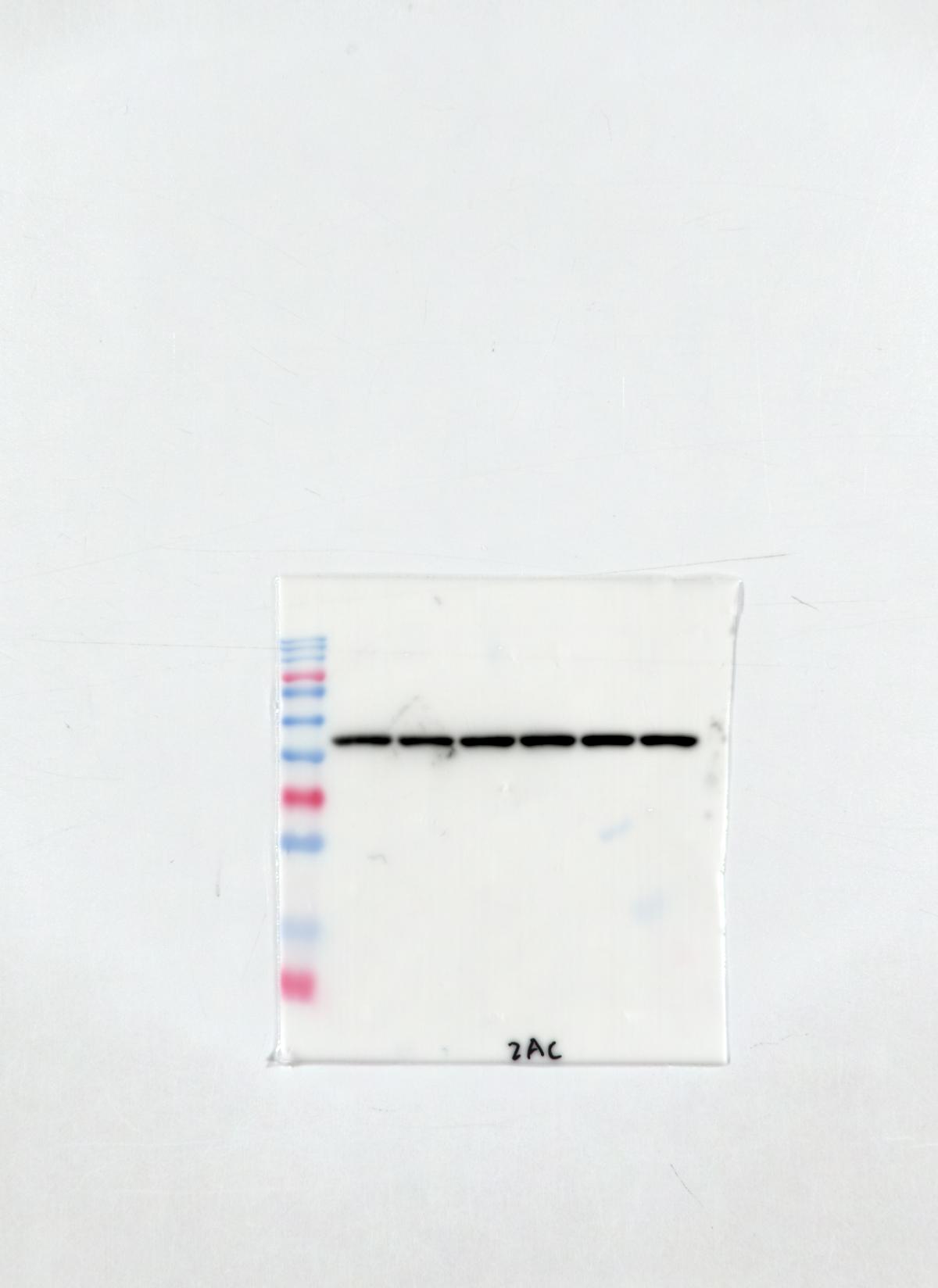

Supplement: Supplementary file 1 [file biomolecules-15-00538-s001.zip › original image/Fig4B.a┬-actin.jpg]

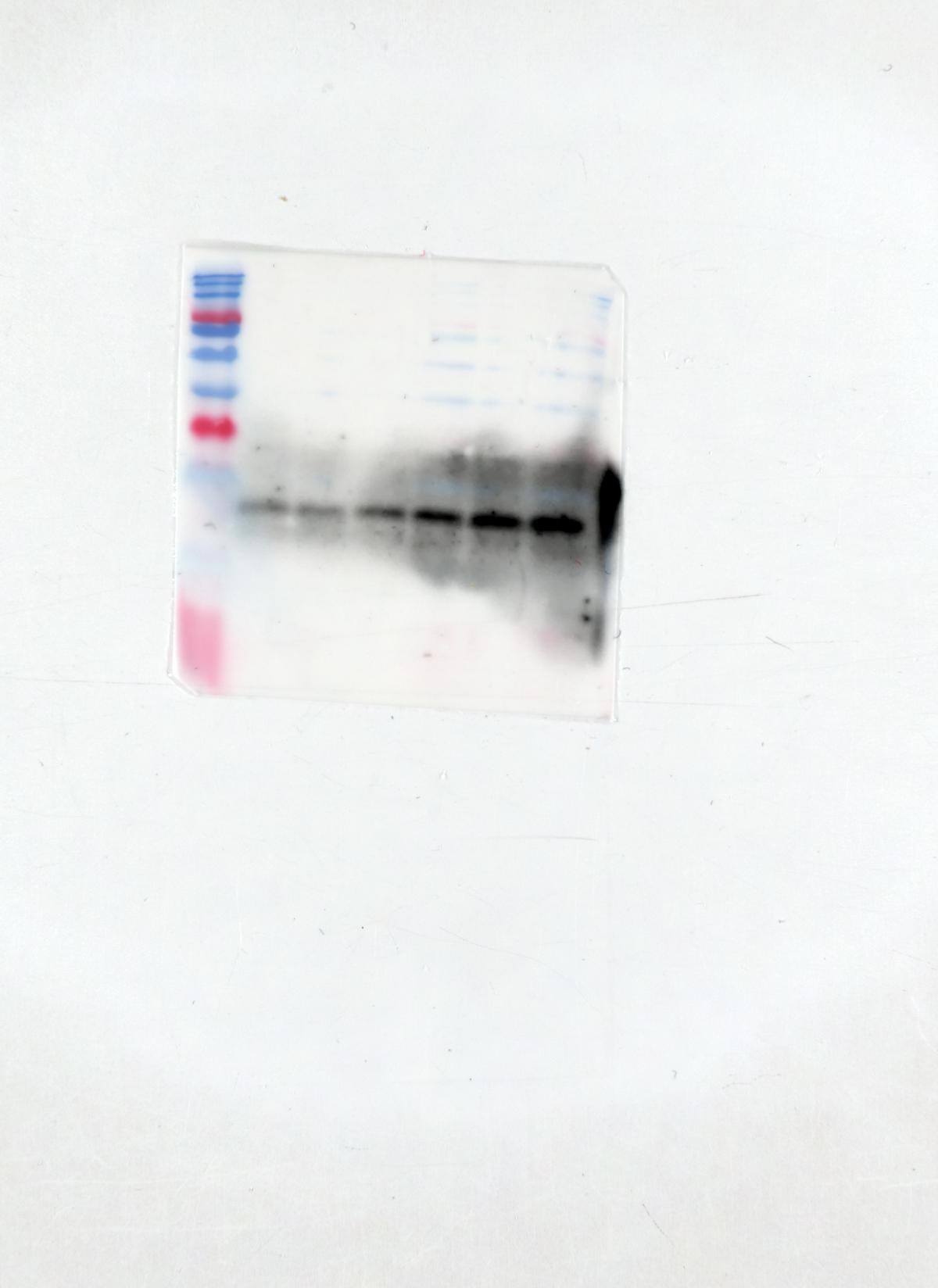

Supplement: Supplementary file 1 [file biomolecules-15-00538-s001.zip › original image/Fig4E.ARPC3.jpg]

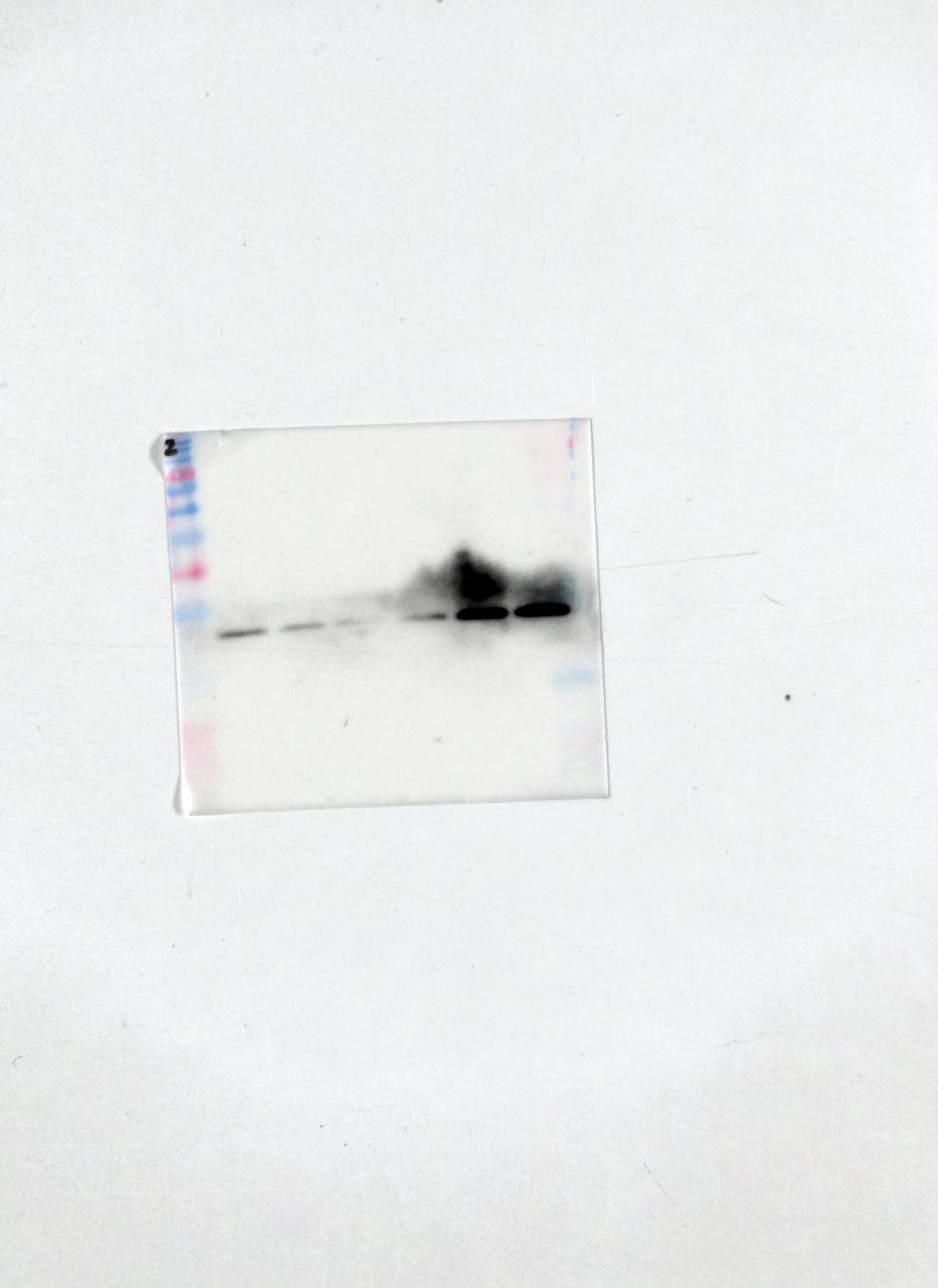

Supplement: Supplementary file 1 [file biomolecules-15-00538-s001.zip › original image/Fig4E.ARPC4.jpg]

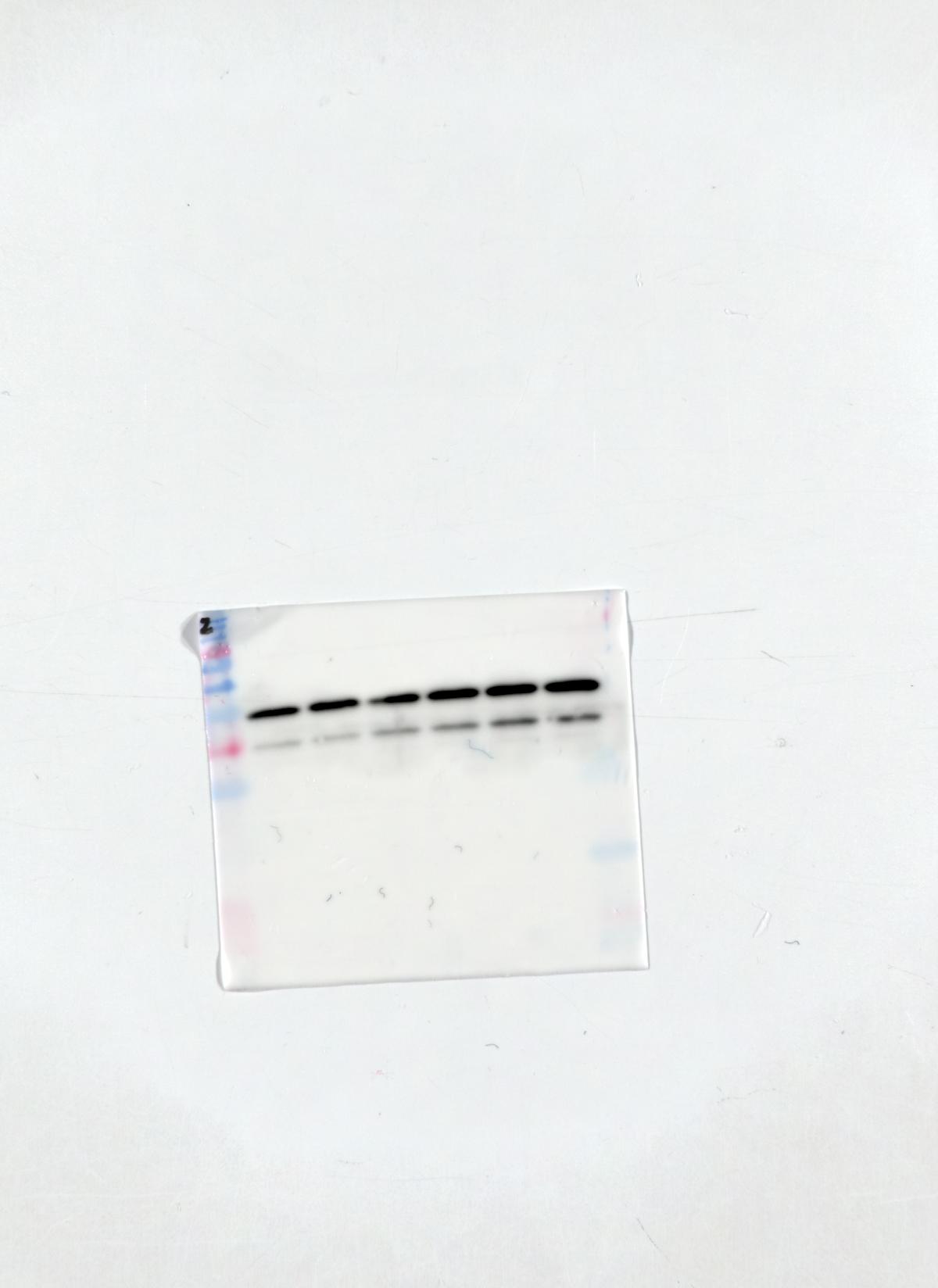

Supplement: Supplementary file 1 [file biomolecules-15-00538-s001.zip › original image/Fig4E.GAPDH.jpg]

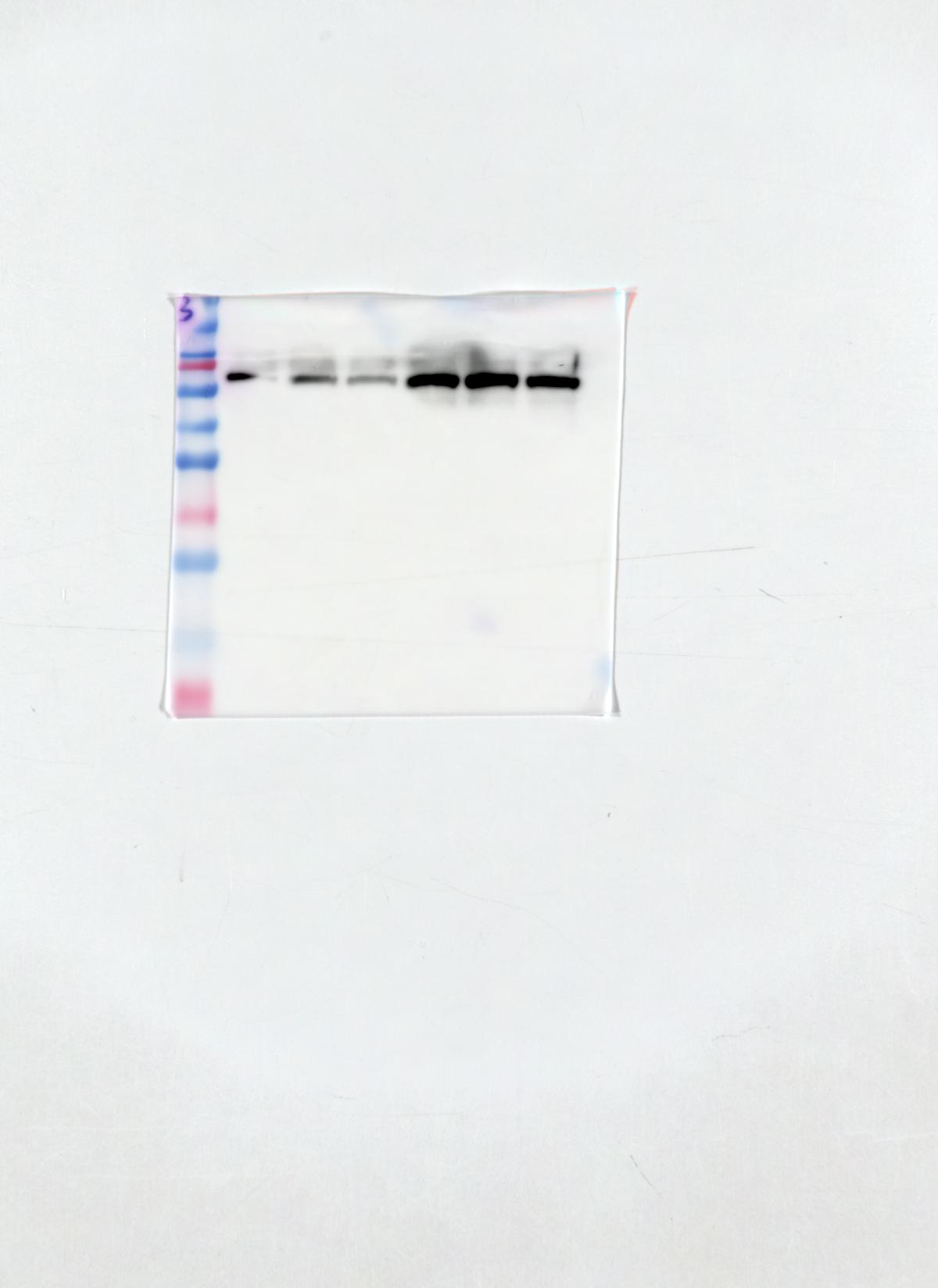

Supplement: Supplementary file 1 [file biomolecules-15-00538-s001.zip › original image/Fig4E.HSPA1A.jpg]

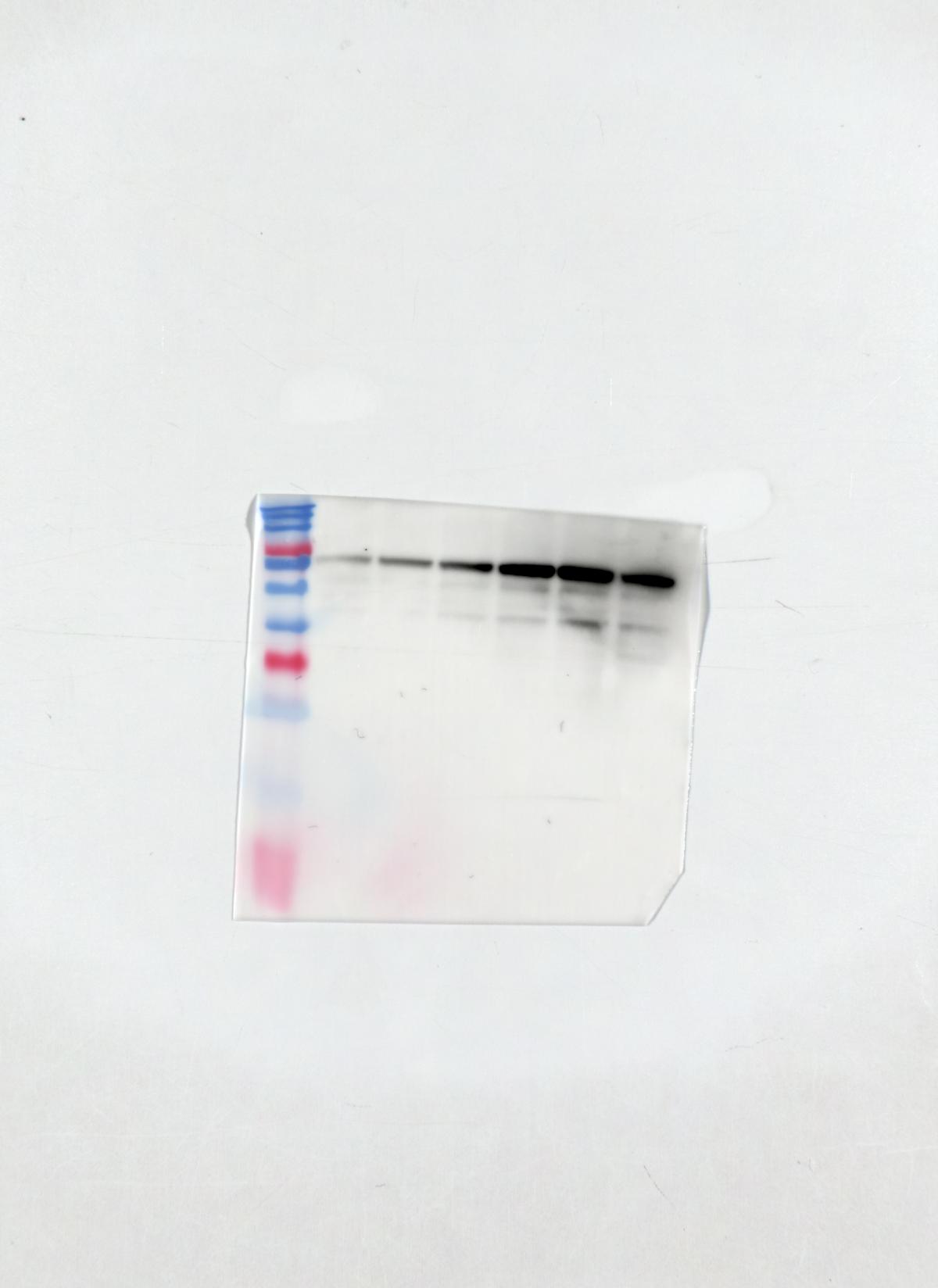

Supplement: Supplementary file 1 [file biomolecules-15-00538-s001.zip › original image/Fig4E.HSPA1L.jpg]

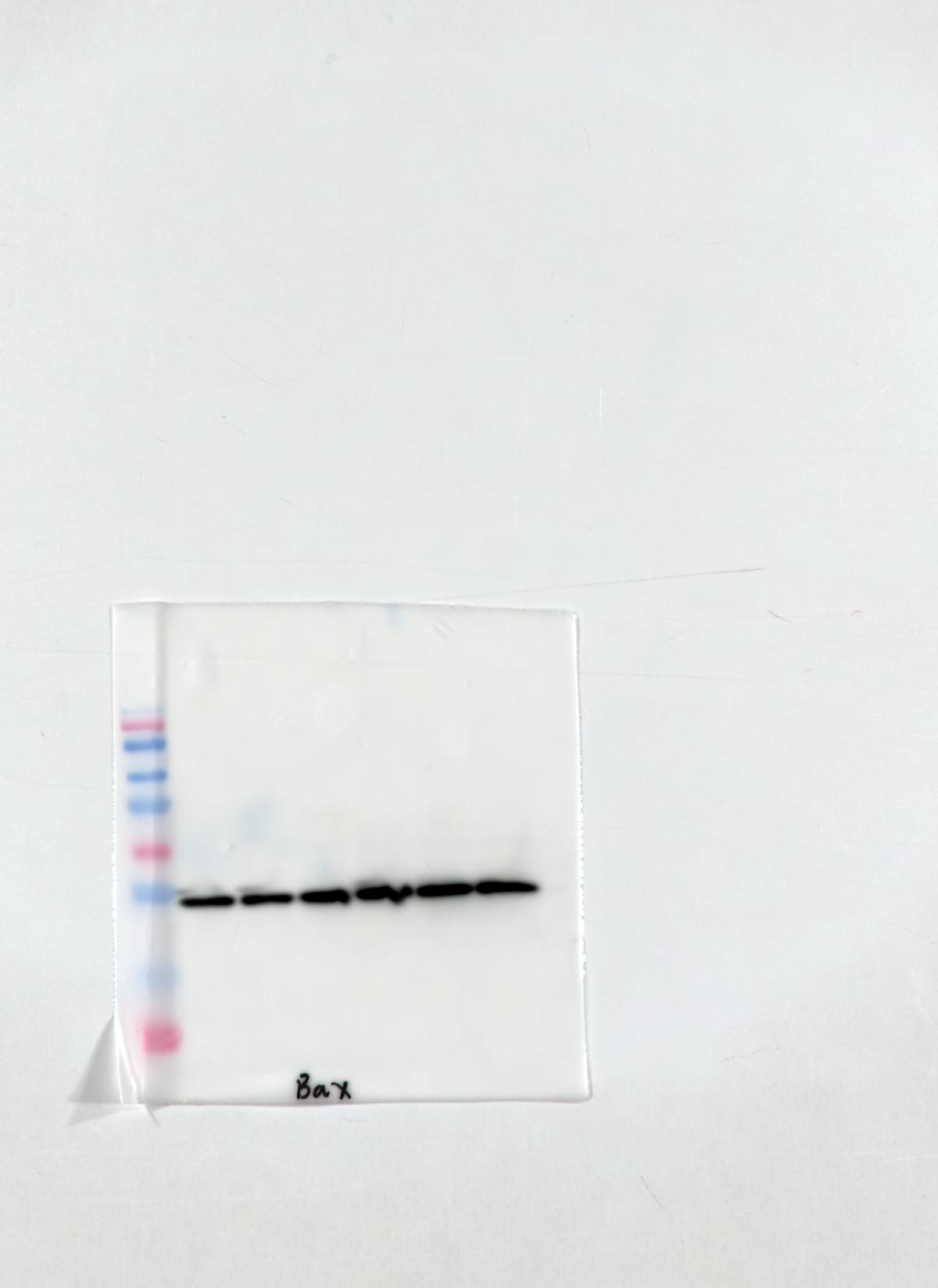

Supplement: Supplementary file 1 [file biomolecules-15-00538-s001.zip › original image/Fig5A.Bax.jpg]

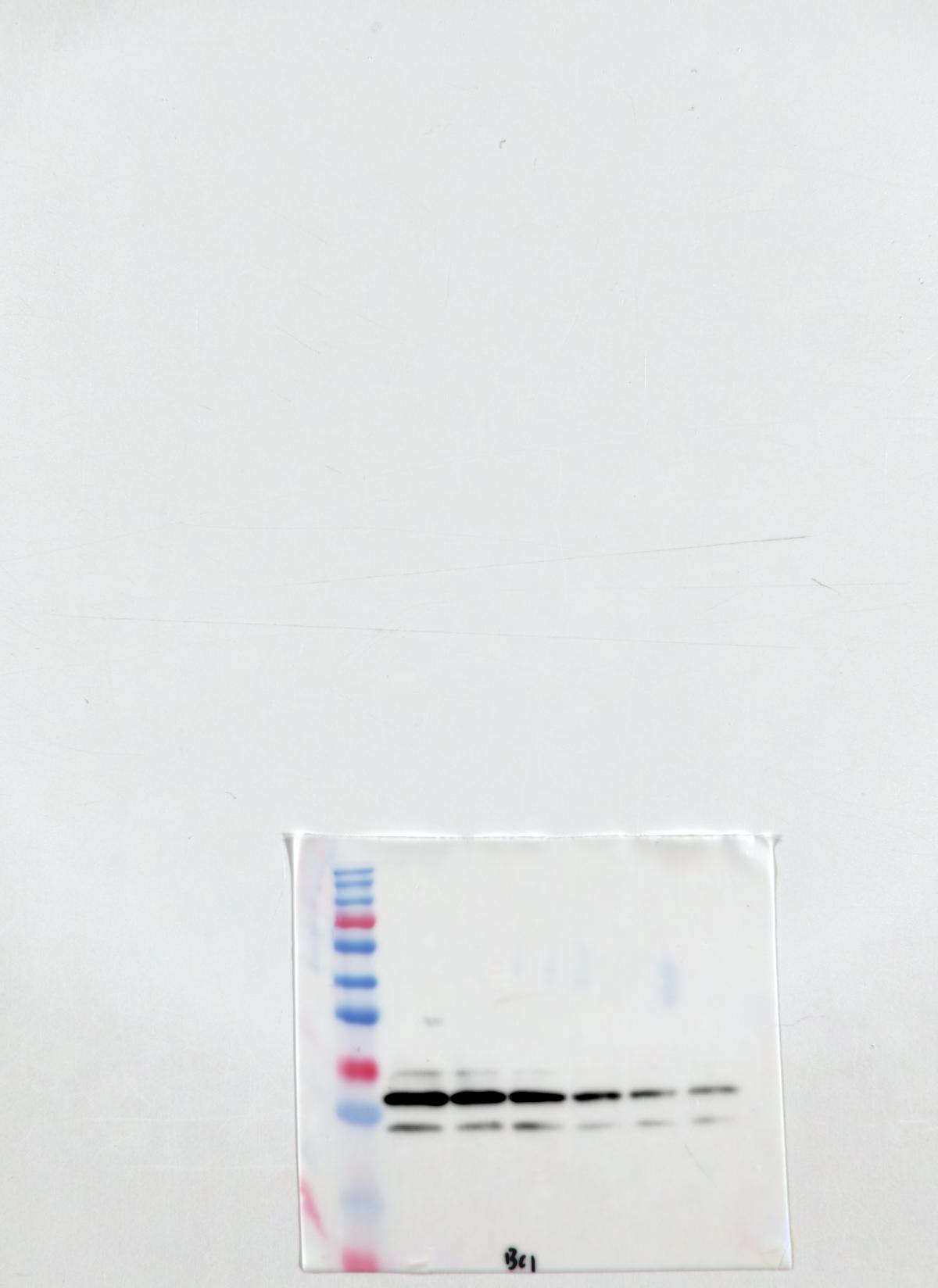

Supplement: Supplementary file 1 [file biomolecules-15-00538-s001.zip › original image/Fig5A.Bcl-2.jpg]

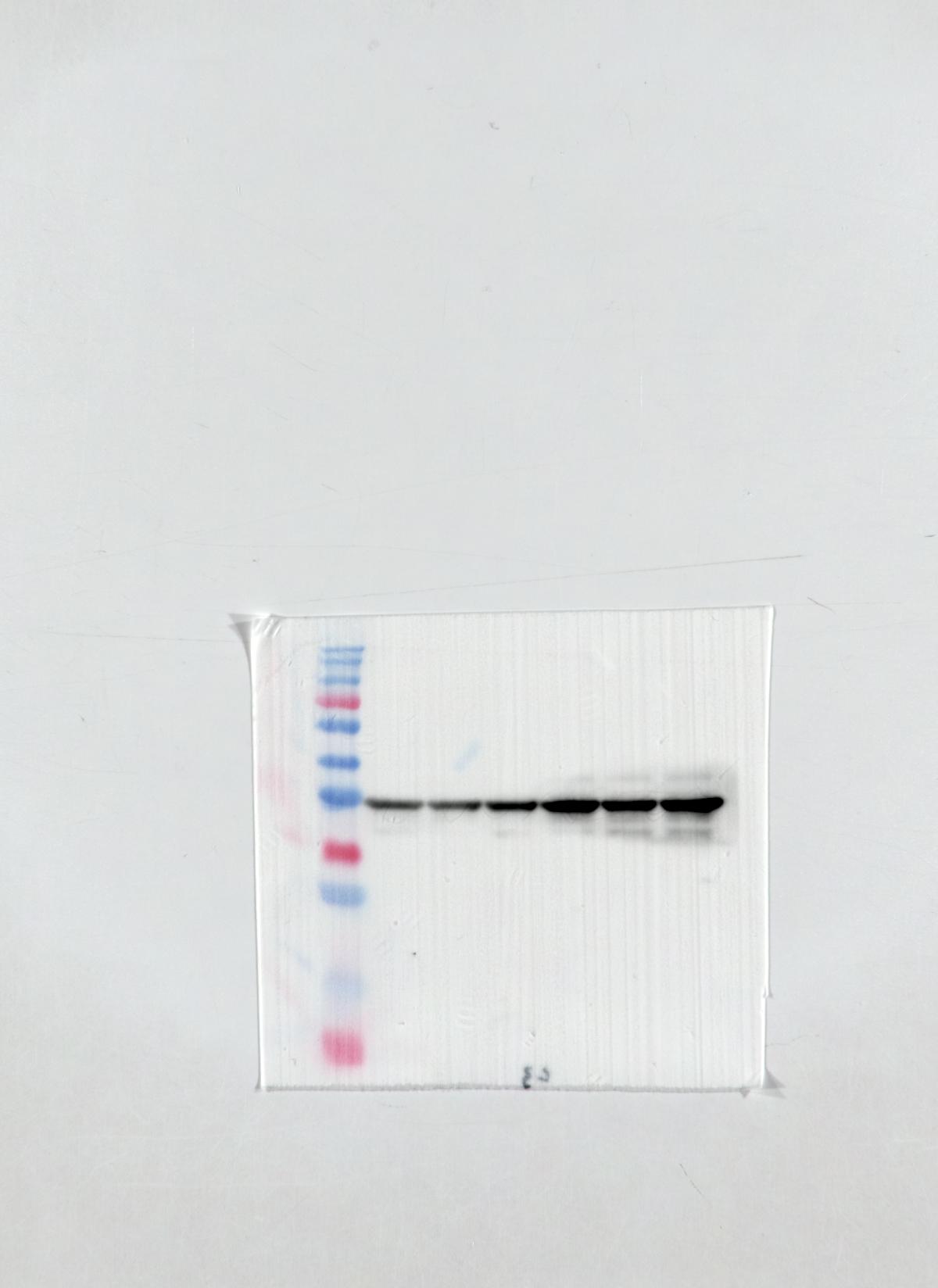

Supplement: Supplementary file 1 [file biomolecules-15-00538-s001.zip › original image/Fig5A.Caspase3.jpg]

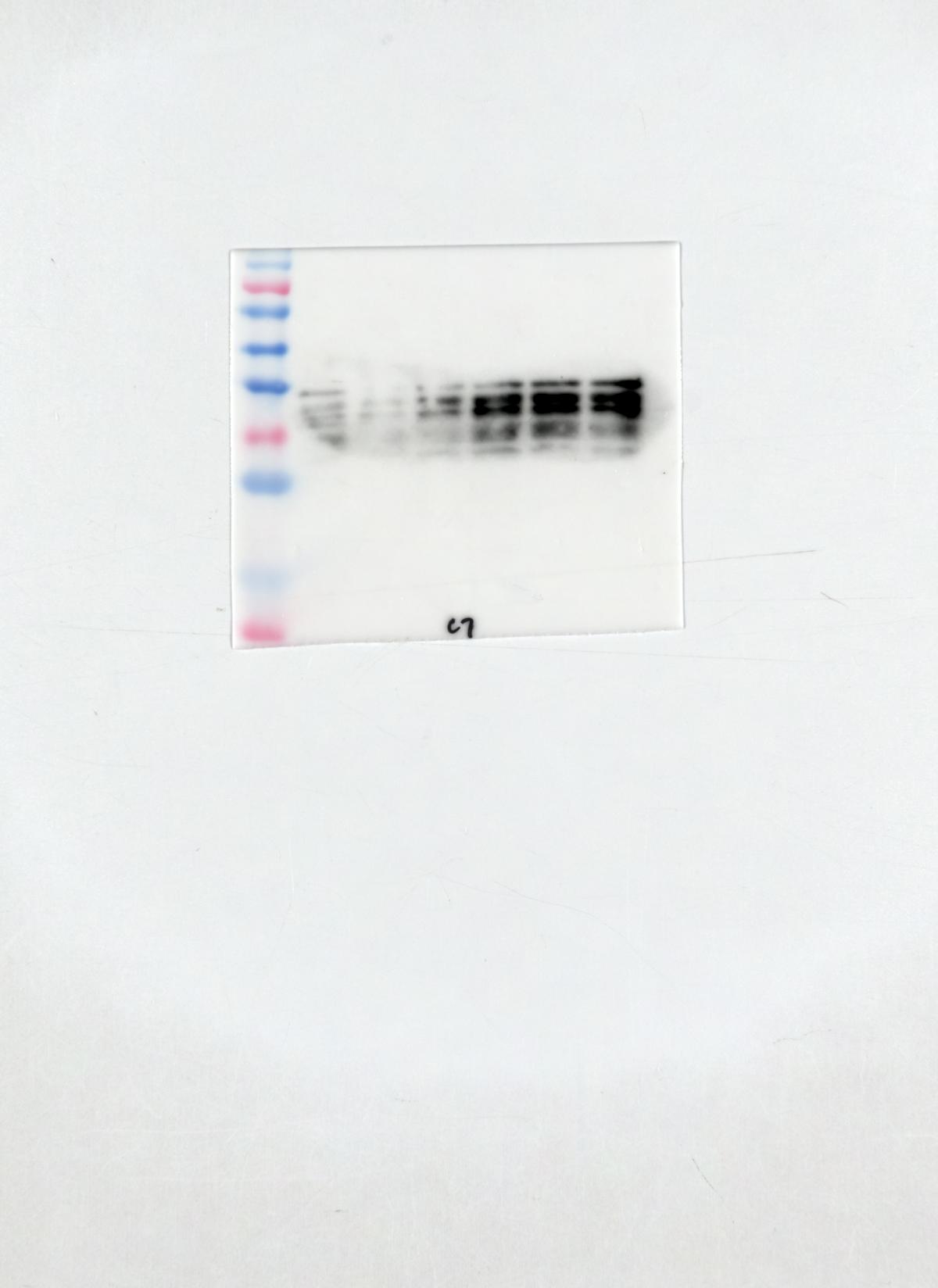

Supplement: Supplementary file 1 [file biomolecules-15-00538-s001.zip › original image/Fig5A.Caspase7.jpg]

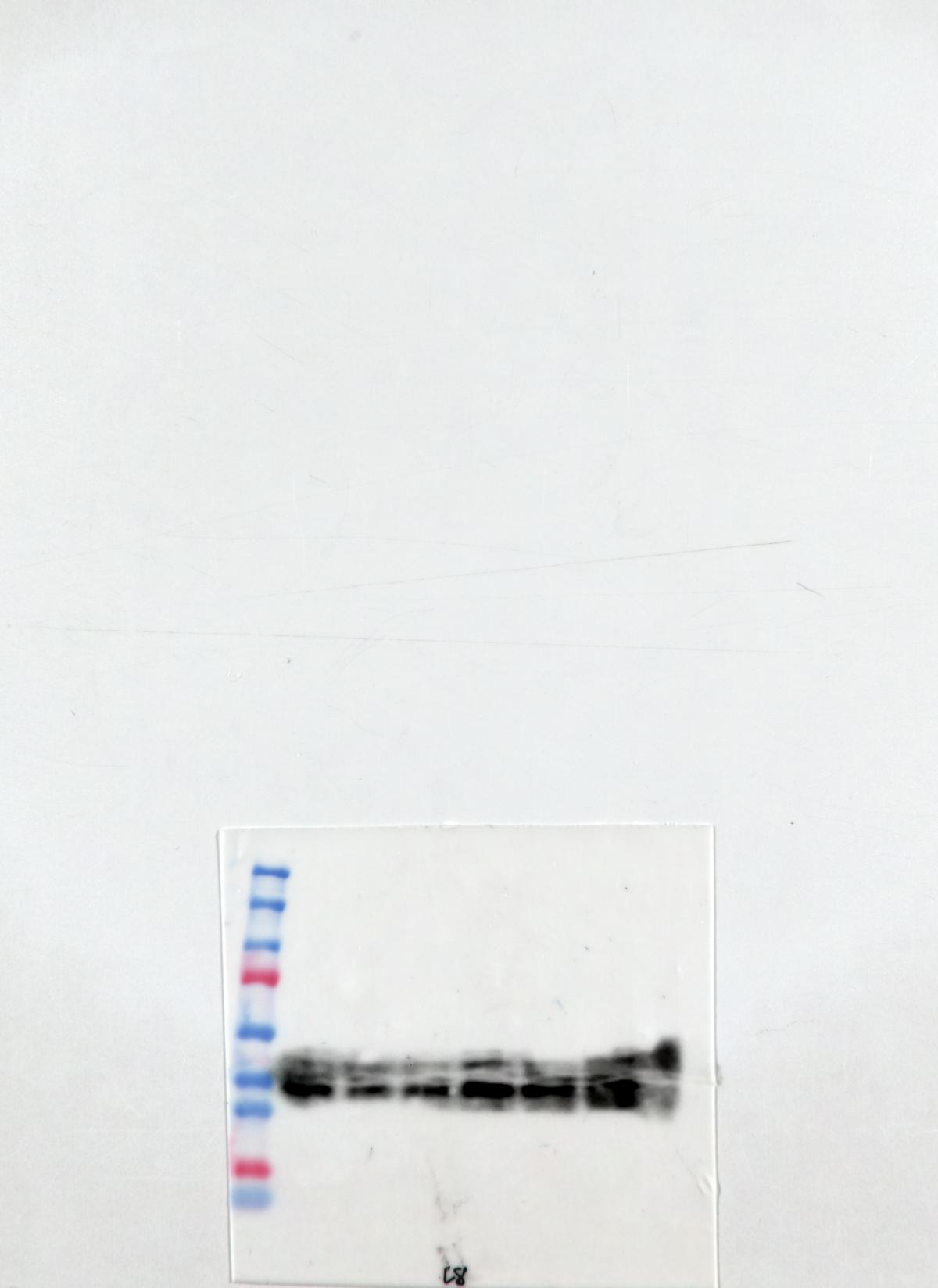

Supplement: Supplementary file 1 [file biomolecules-15-00538-s001.zip › original image/Fig5A.Caspase8.jpg]

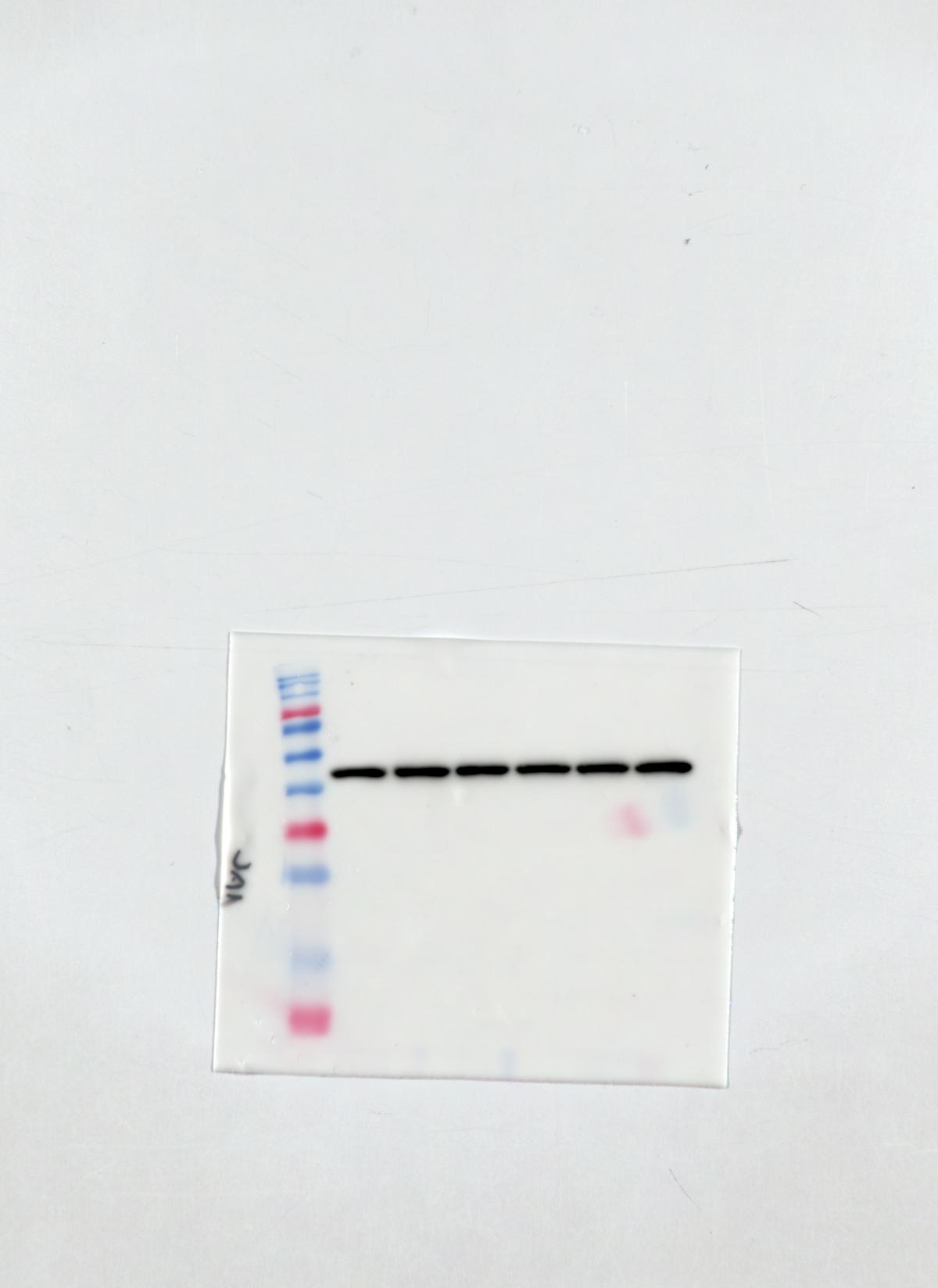

Supplement: Supplementary file 1 [file biomolecules-15-00538-s001.zip › original image/Fig5A.a┬-actin.jpg]

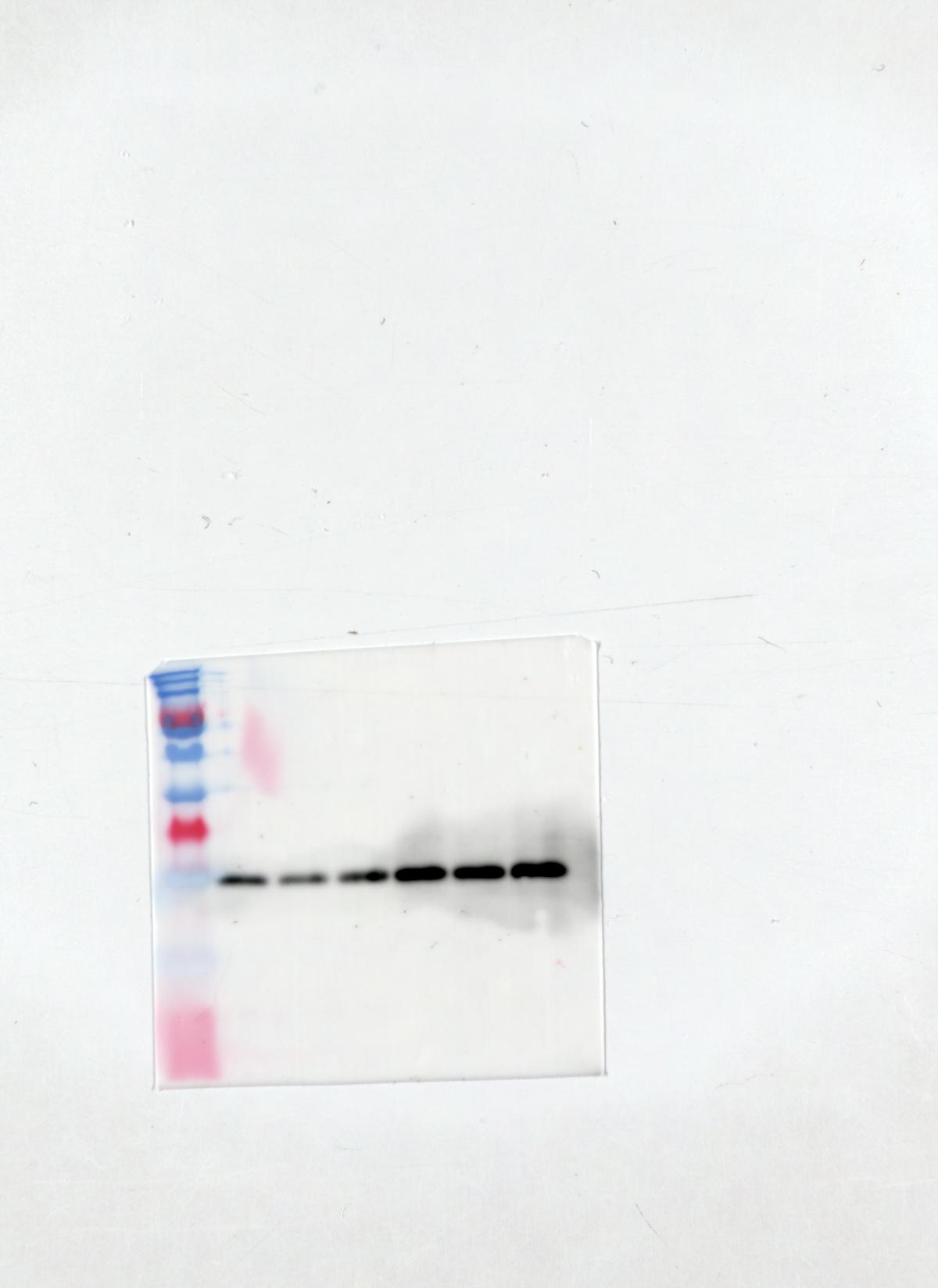

Supplement: Supplementary file 1 [file biomolecules-15-00538-s001.zip › original image/Fig5C.Bax.jpg]

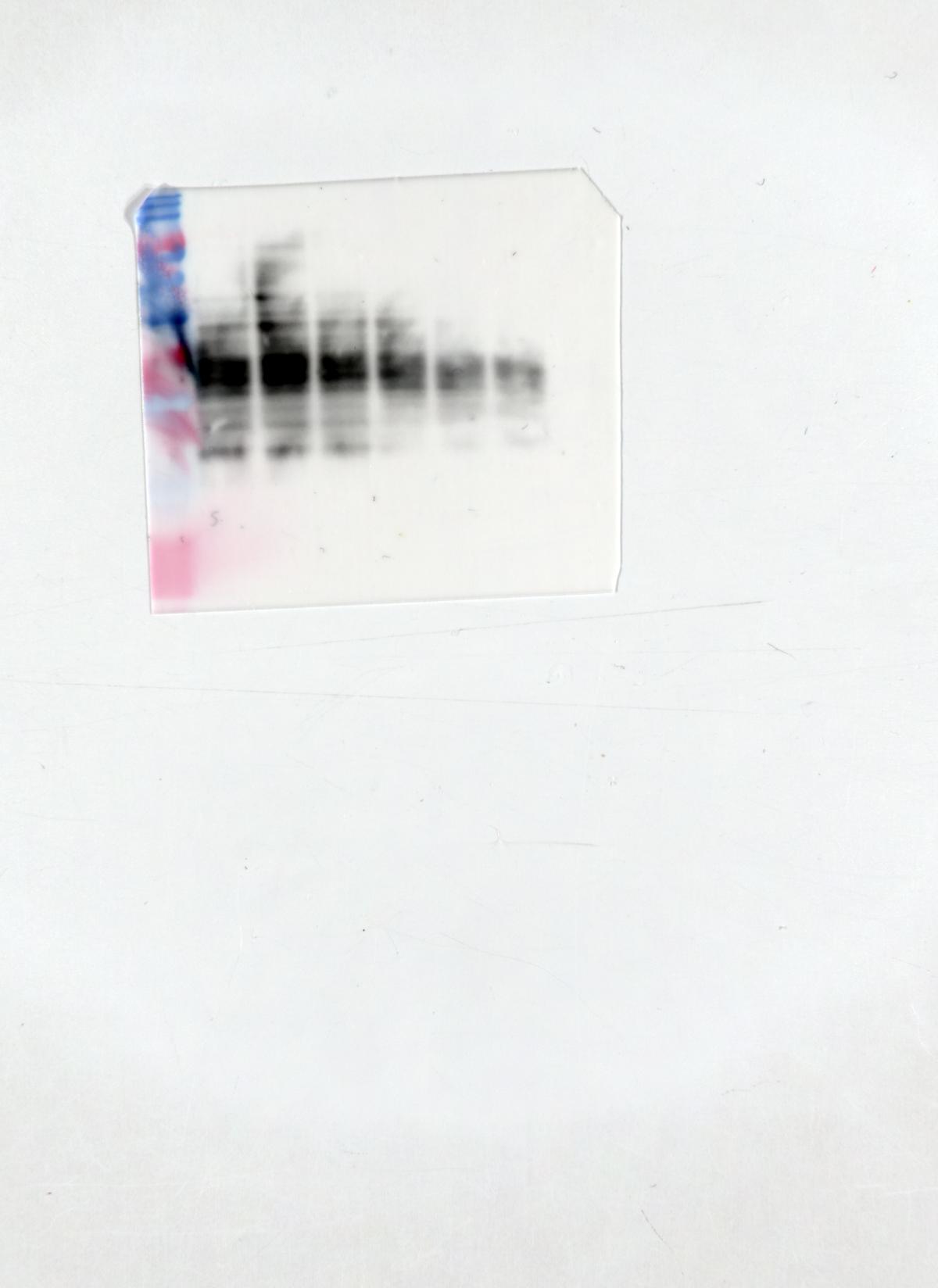

Supplement: Supplementary file 1 [file biomolecules-15-00538-s001.zip › original image/Fig5C.Bcl-2.jpg]

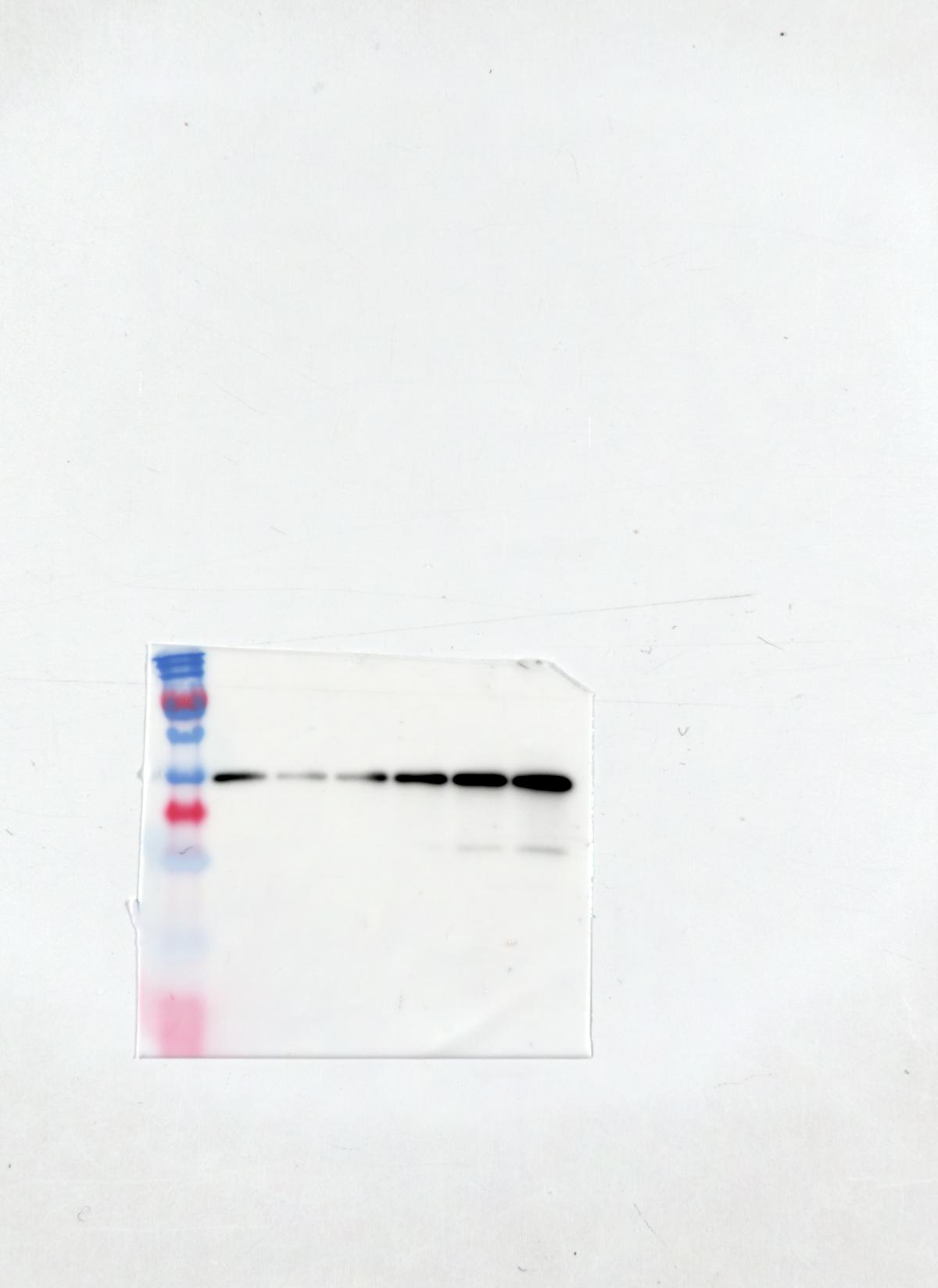

Supplement: Supplementary file 1 [file biomolecules-15-00538-s001.zip › original image/Fig5C.Caspase3.jpg]

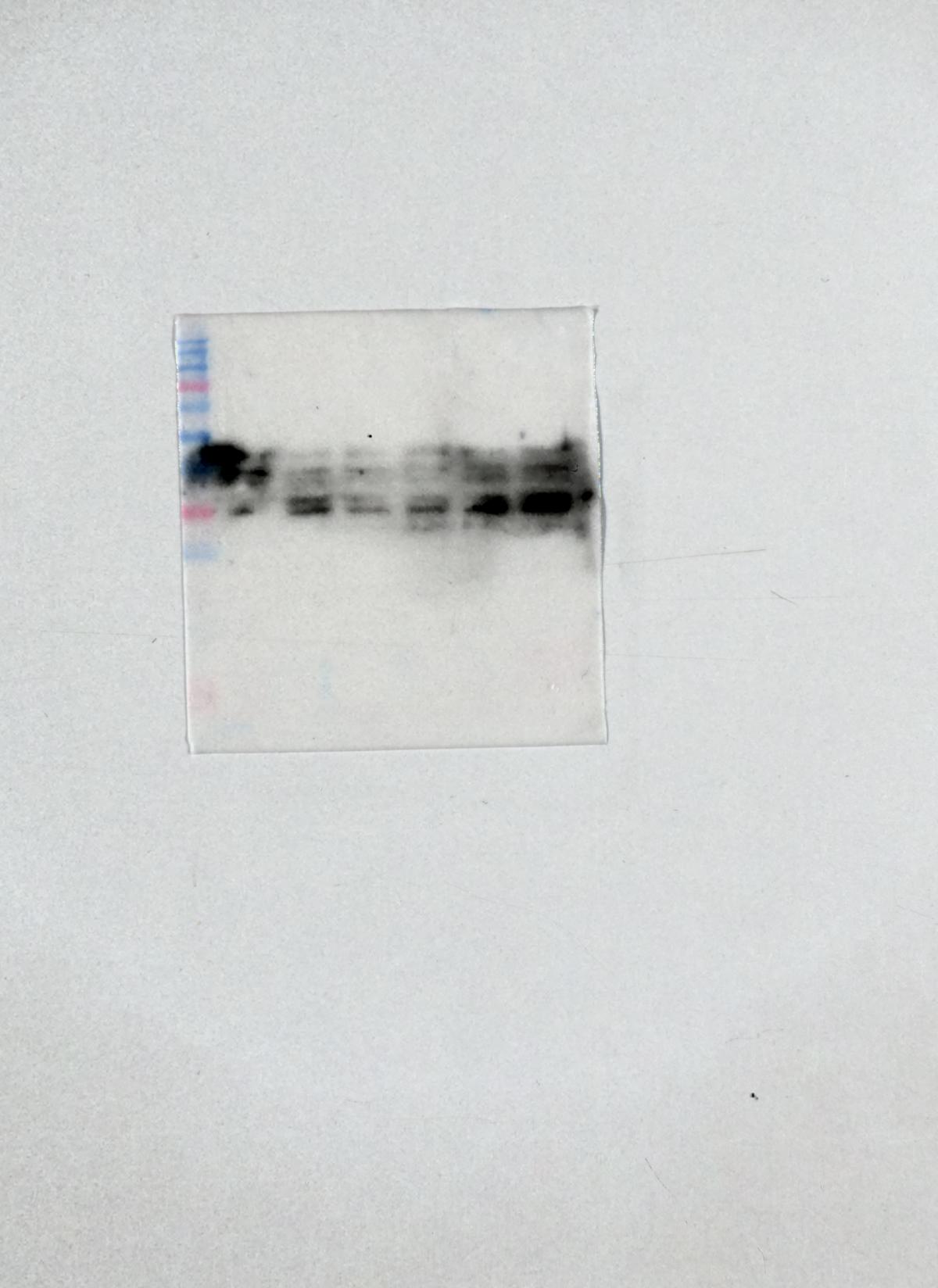

Supplement: Supplementary file 1 [file biomolecules-15-00538-s001.zip › original image/Fig5C.Caspase7.jpg]

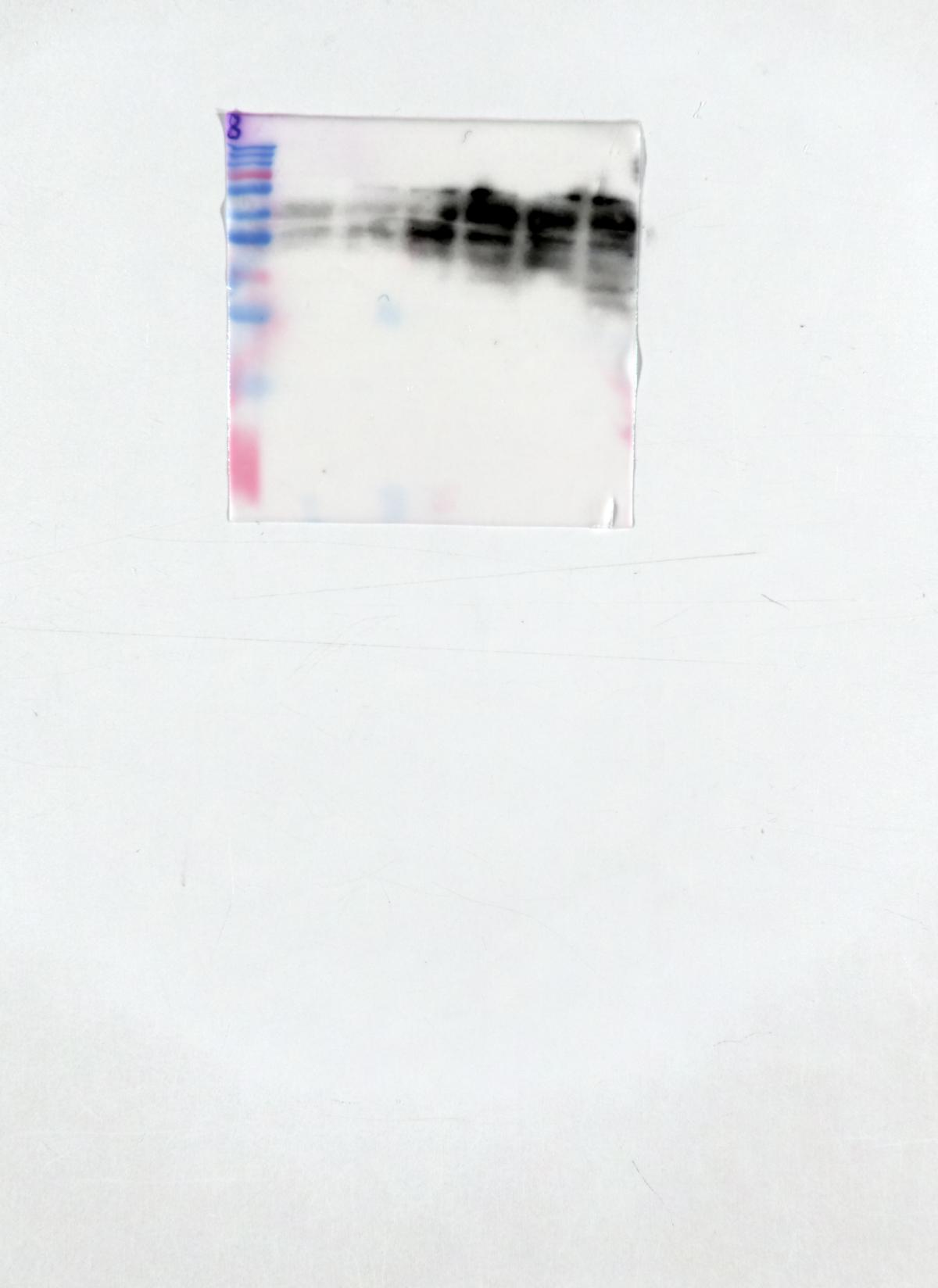

Supplement: Supplementary file 1 [file biomolecules-15-00538-s001.zip › original image/Fig5C.Caspase8.jpg]

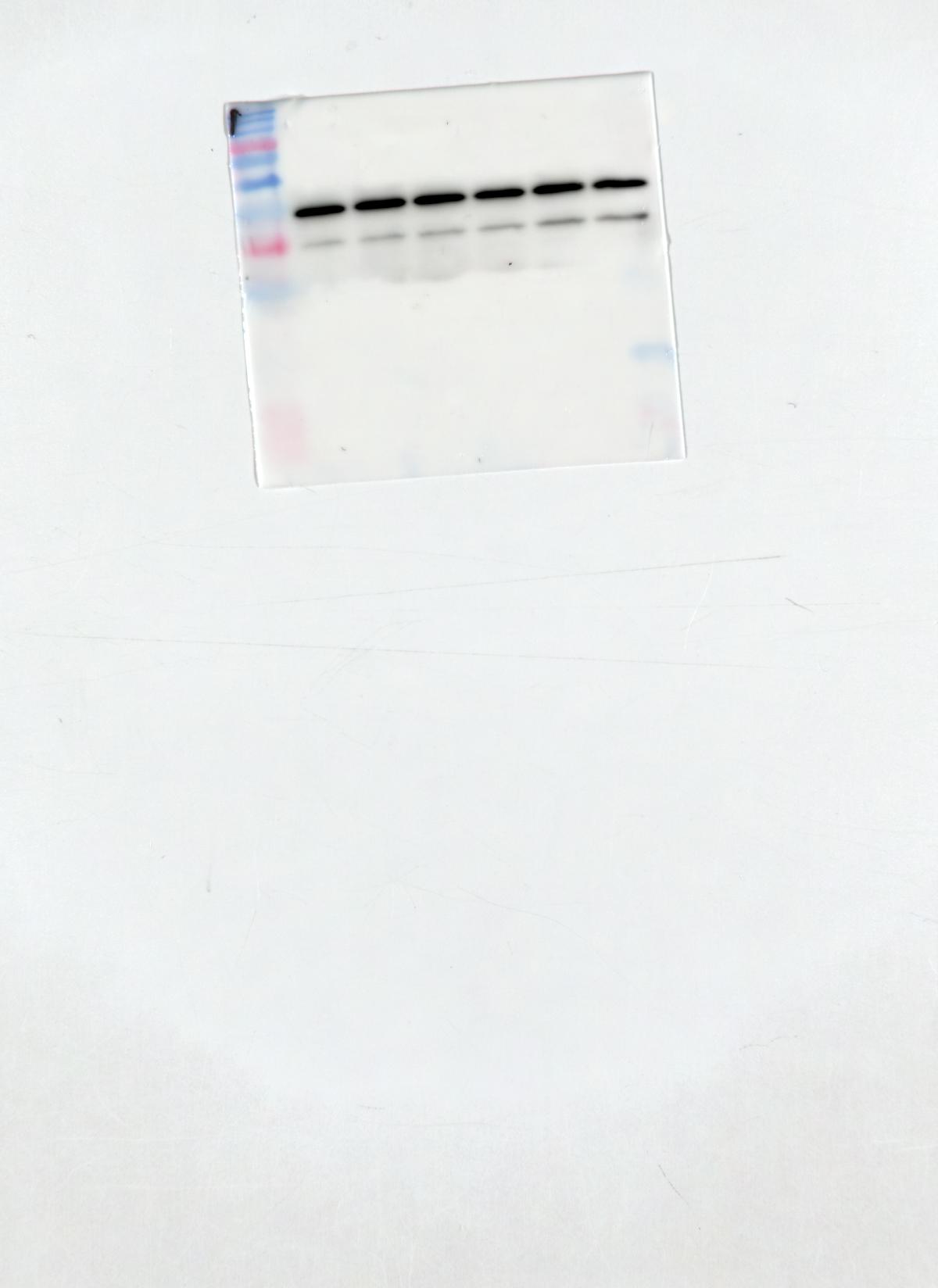

Supplement: Supplementary file 1 [file biomolecules-15-00538-s001.zip › original image/Fig5C.GAPDH.jpg]

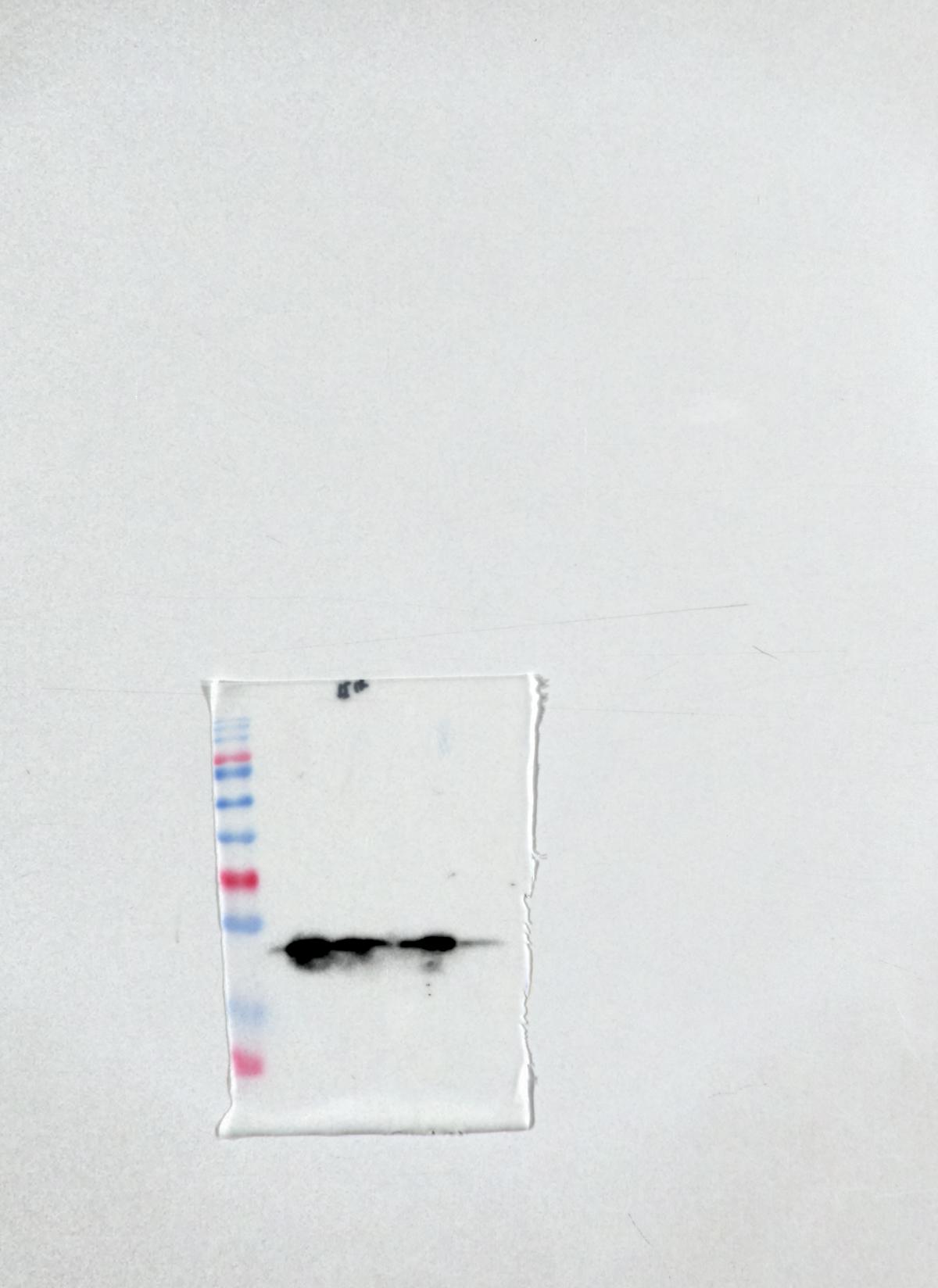

Supplement: Supplementary file 1 [file biomolecules-15-00538-s001.zip › original image/Fig6C.ARPC3.jpg]

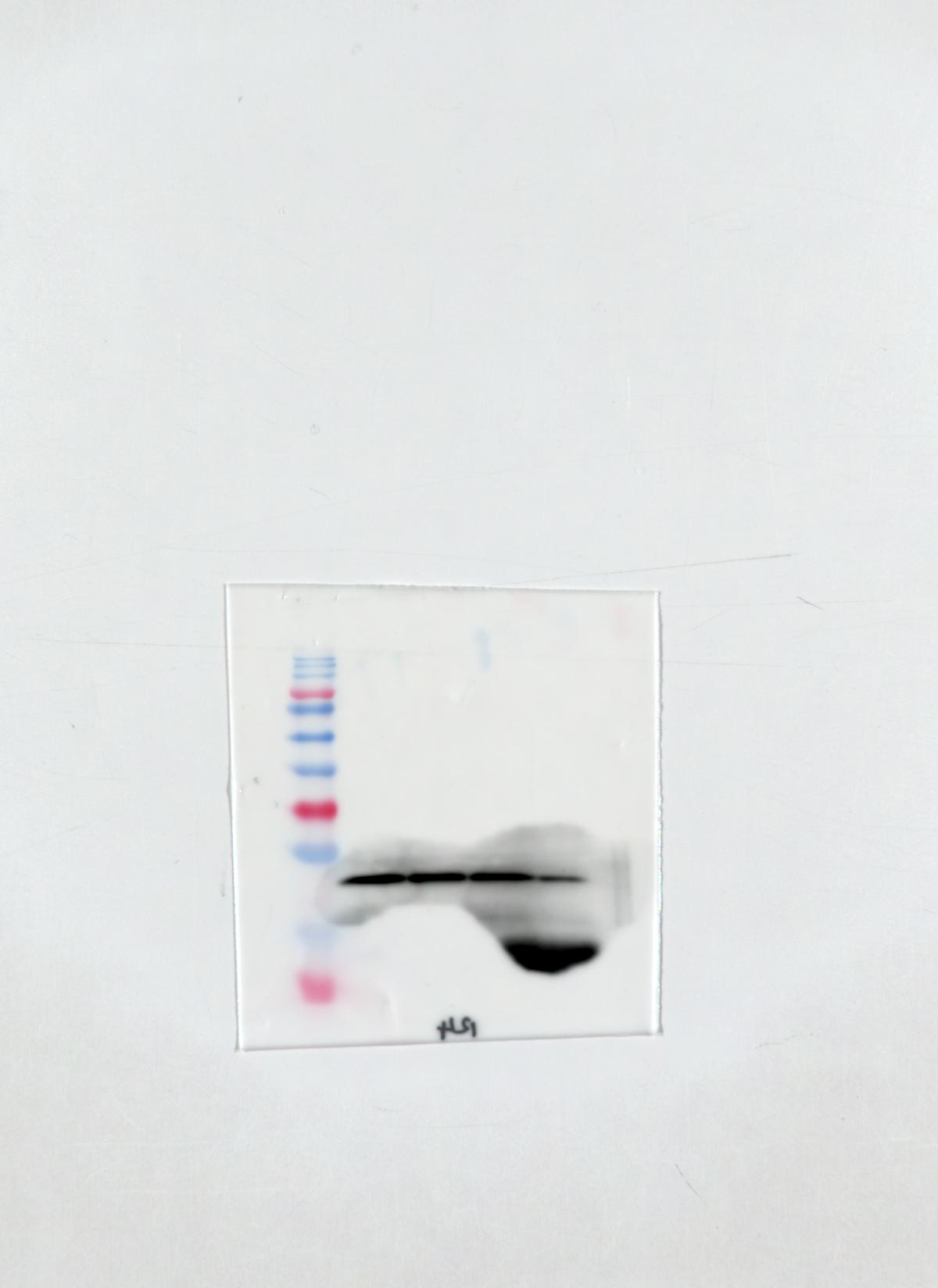

Supplement: Supplementary file 1 [file biomolecules-15-00538-s001.zip › original image/Fig6C.ARPC4.jpg]

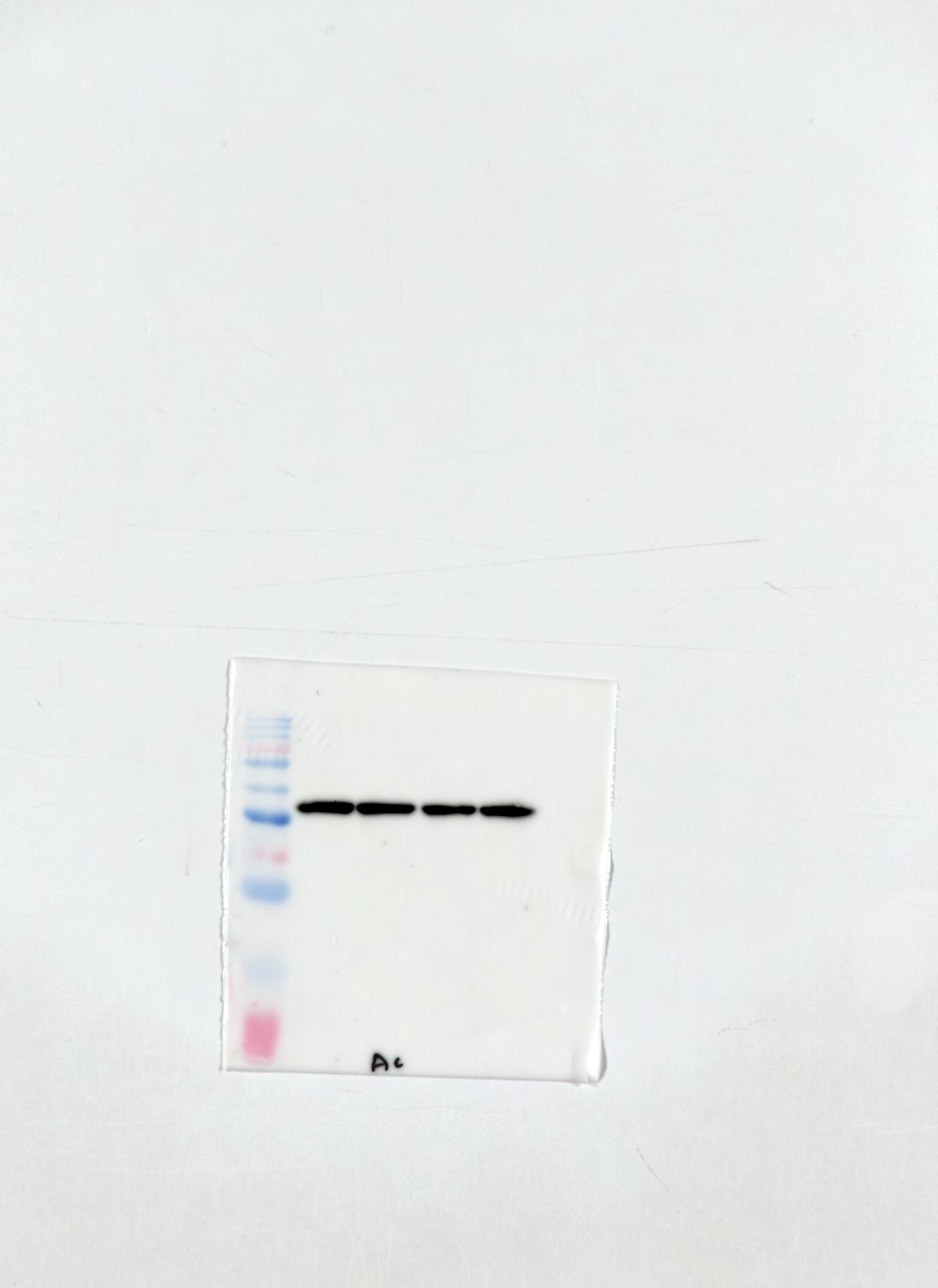

Supplement: Supplementary file 1 [file biomolecules-15-00538-s001.zip › original image/Fig6C.a┬-actin.jpg]

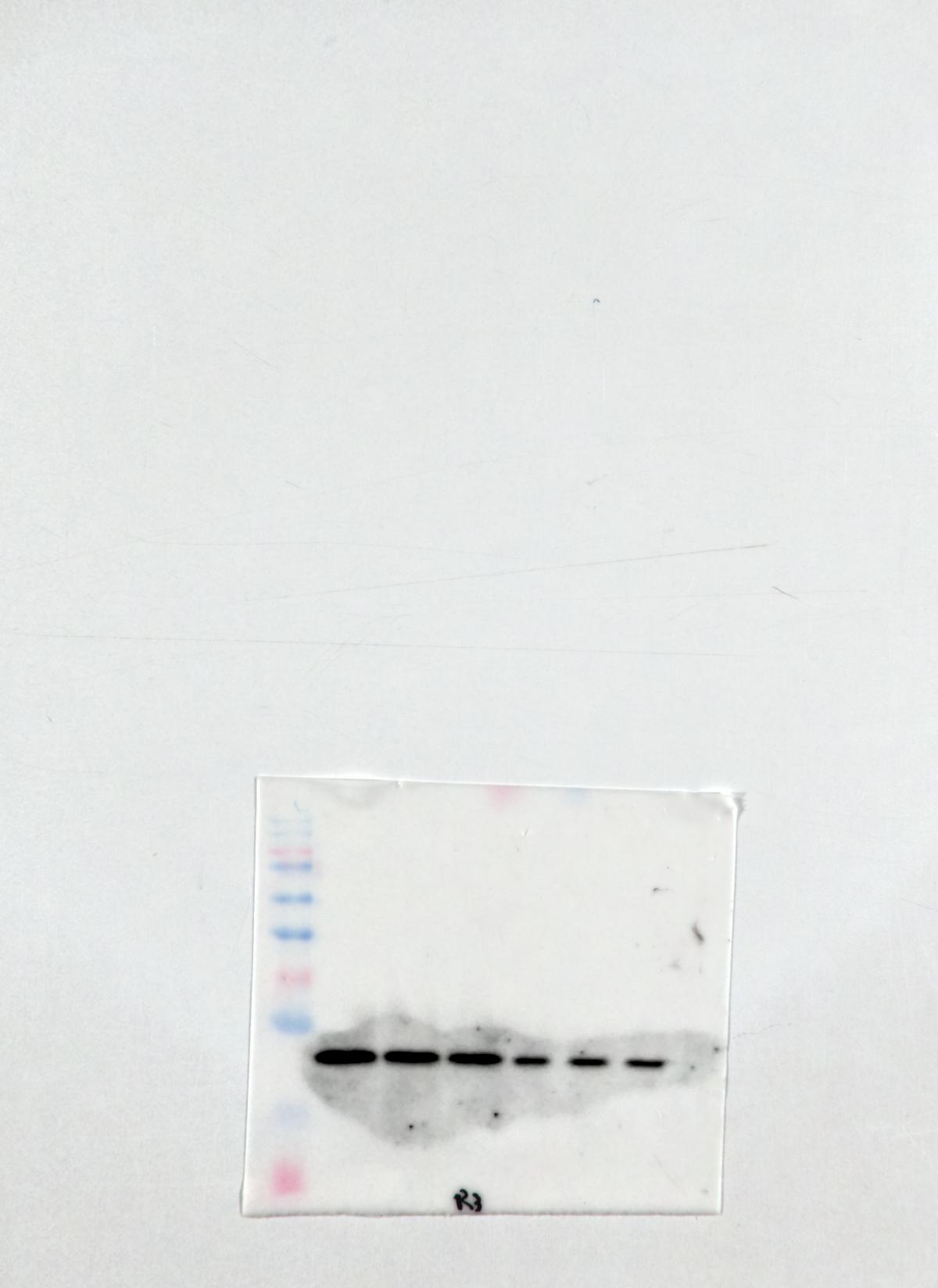

Supplement: Supplementary file 1 [file biomolecules-15-00538-s001.zip › original image/Fig7A.ARPC3.jpg]

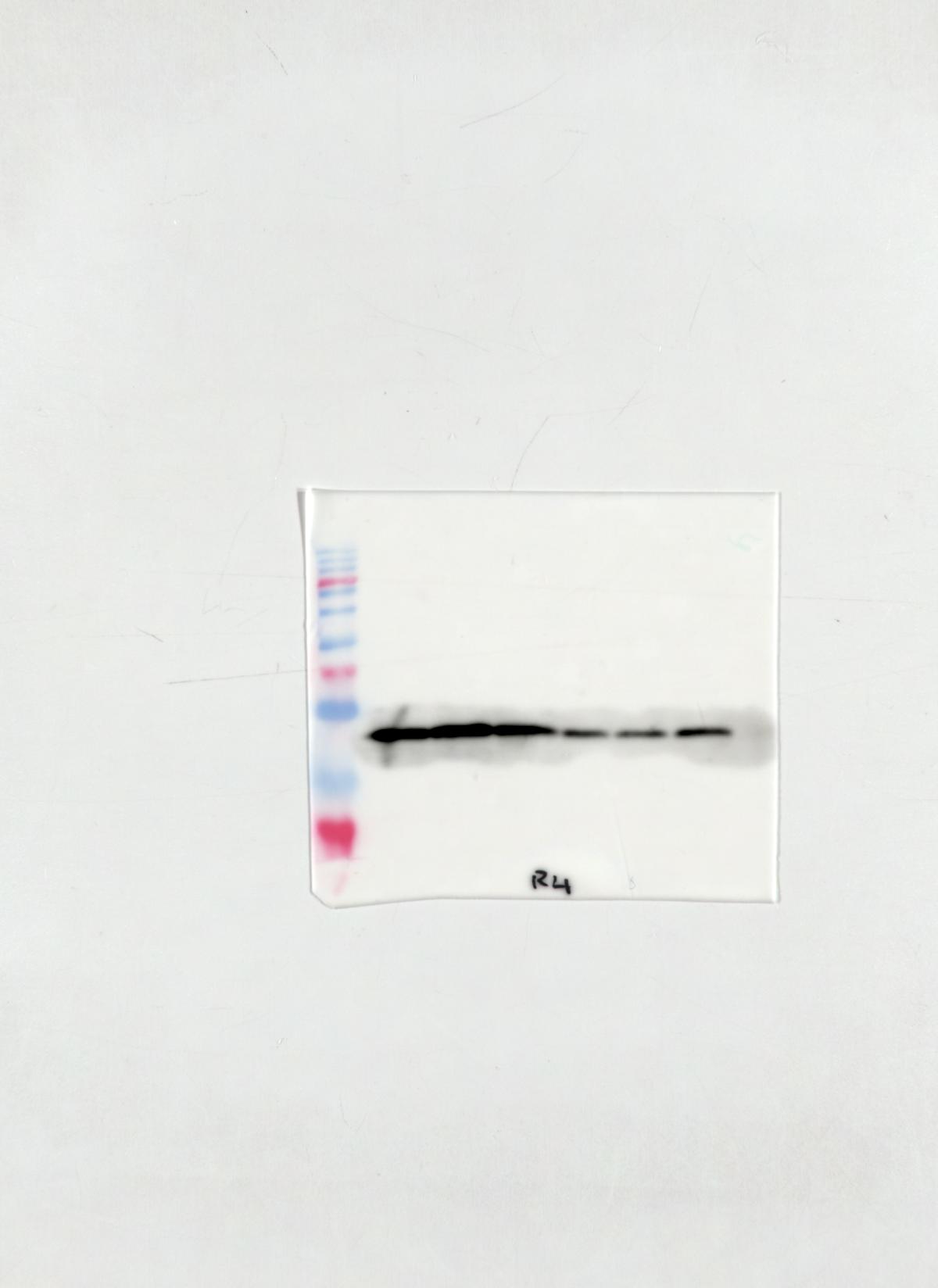

Supplement: Supplementary file 1 [file biomolecules-15-00538-s001.zip › original image/Fig7A.ARPC4.jpg]

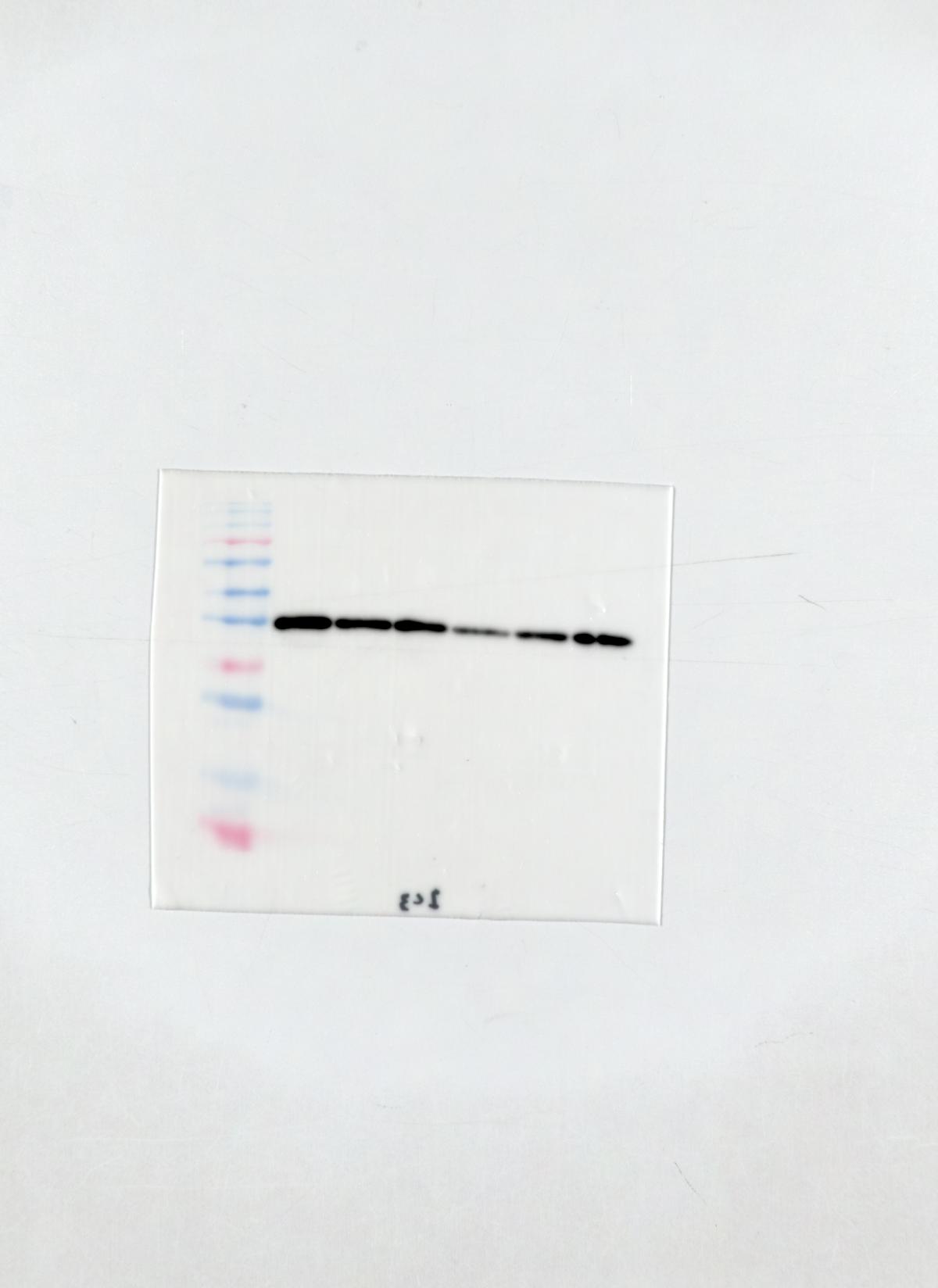

Supplement: Supplementary file 1 [file biomolecules-15-00538-s001.zip › original image/Fig7A.Caspase3.jpg]

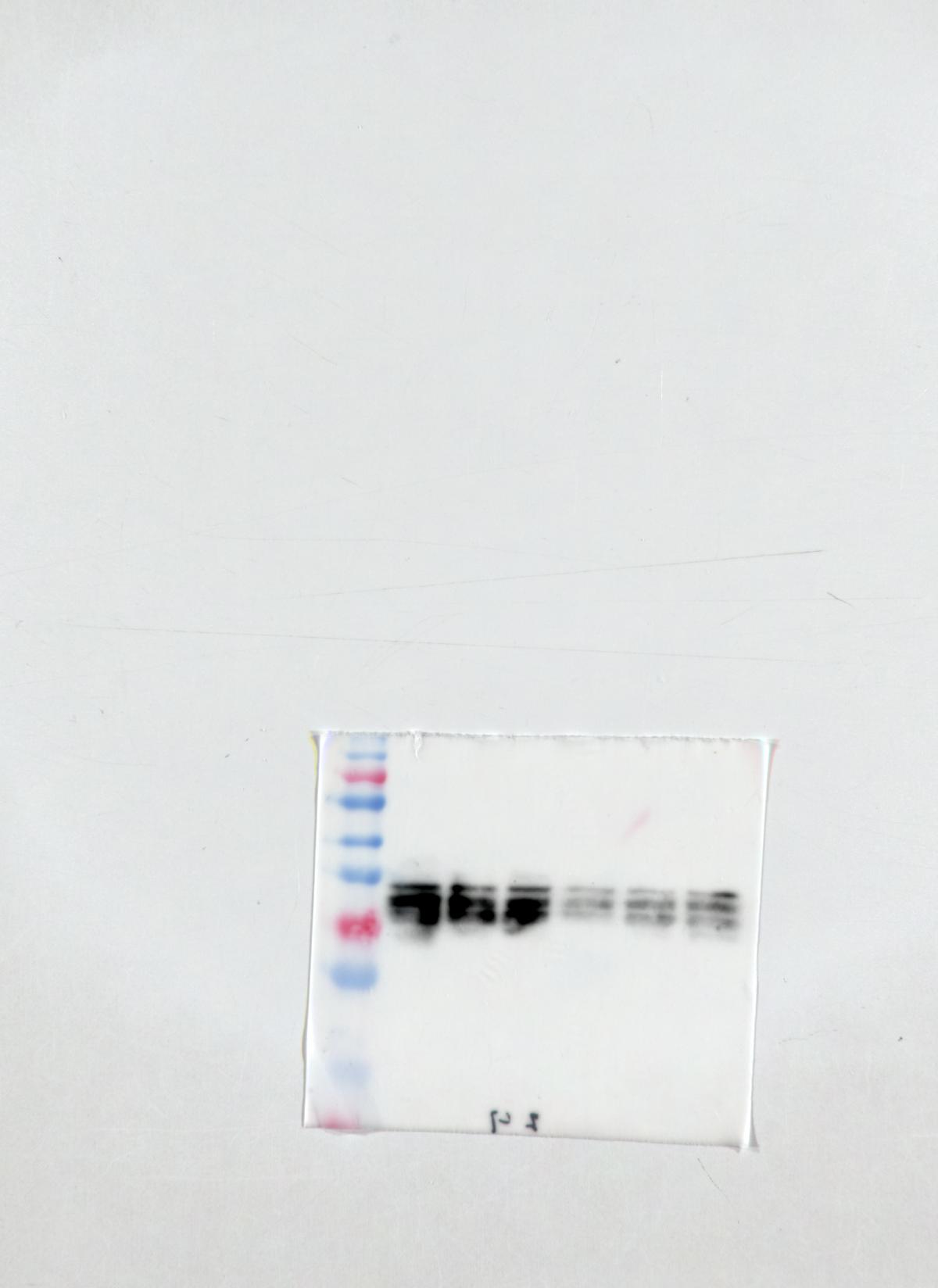

Supplement: Supplementary file 1 [file biomolecules-15-00538-s001.zip › original image/Fig7A.Caspase7.jpg]

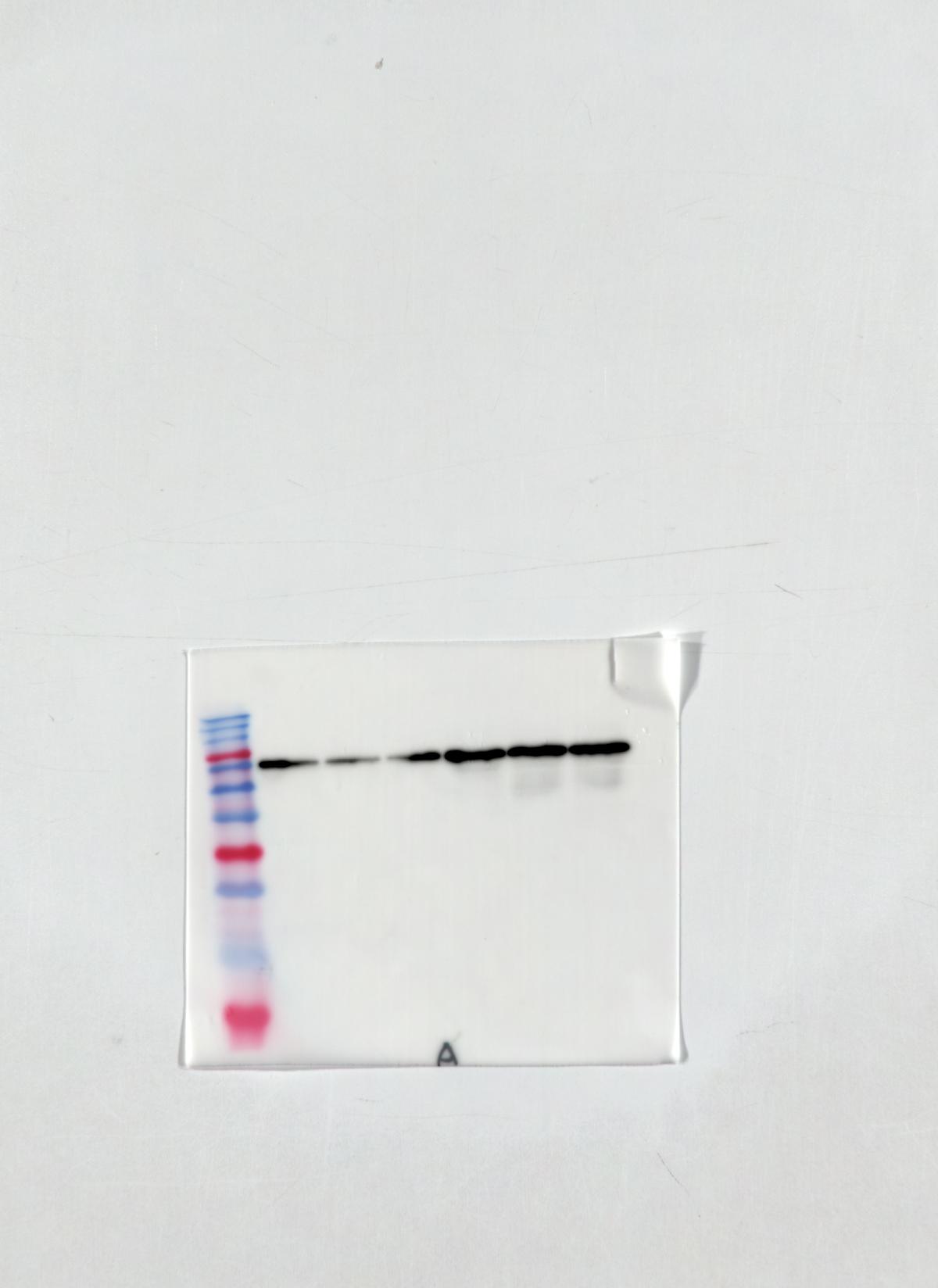

Supplement: Supplementary file 1 [file biomolecules-15-00538-s001.zip › original image/Fig7A.HSPA1A.jpg]

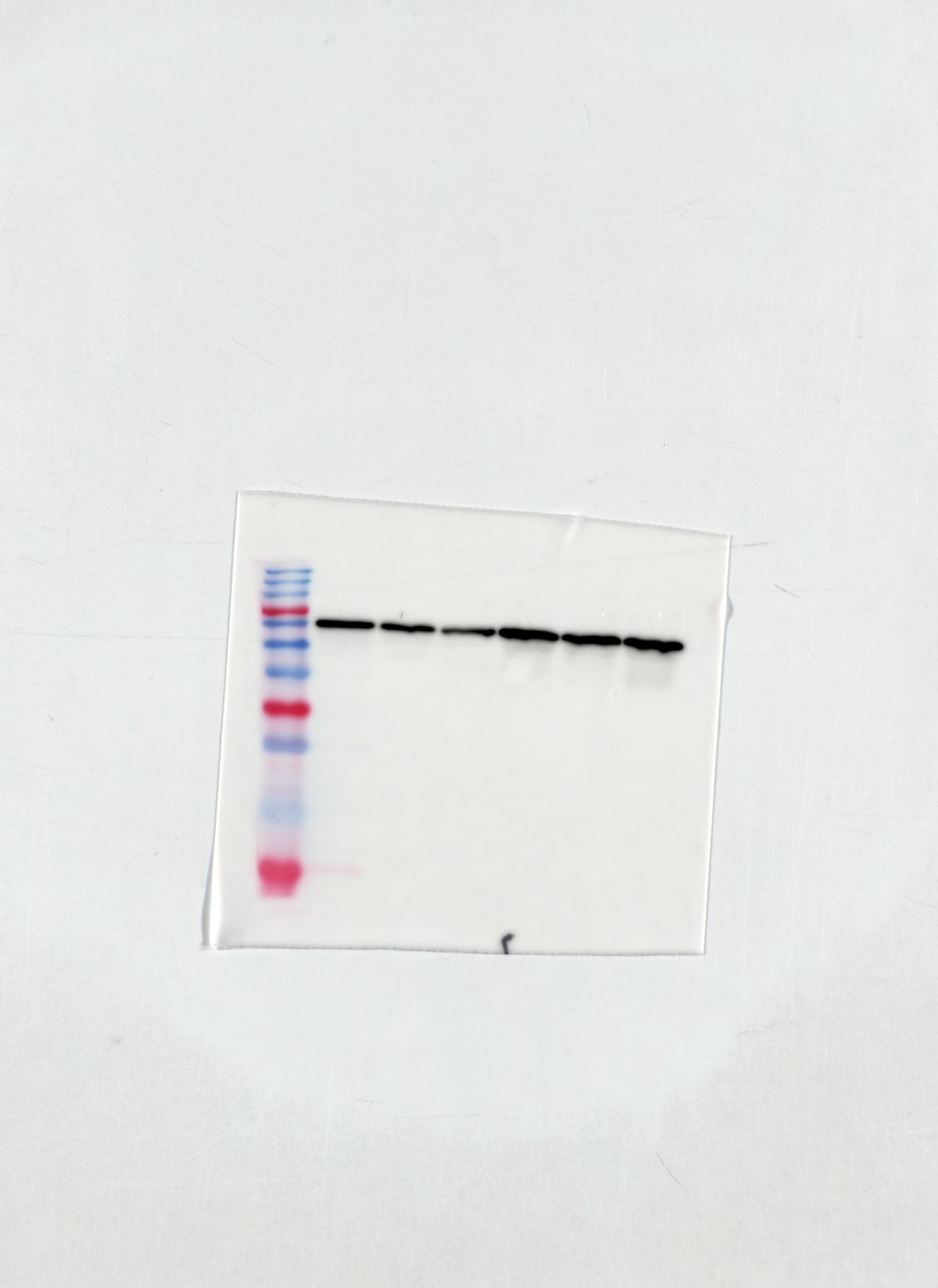

Supplement: Supplementary file 1 [file biomolecules-15-00538-s001.zip › original image/Fig7A.HSPA1L.jpg]

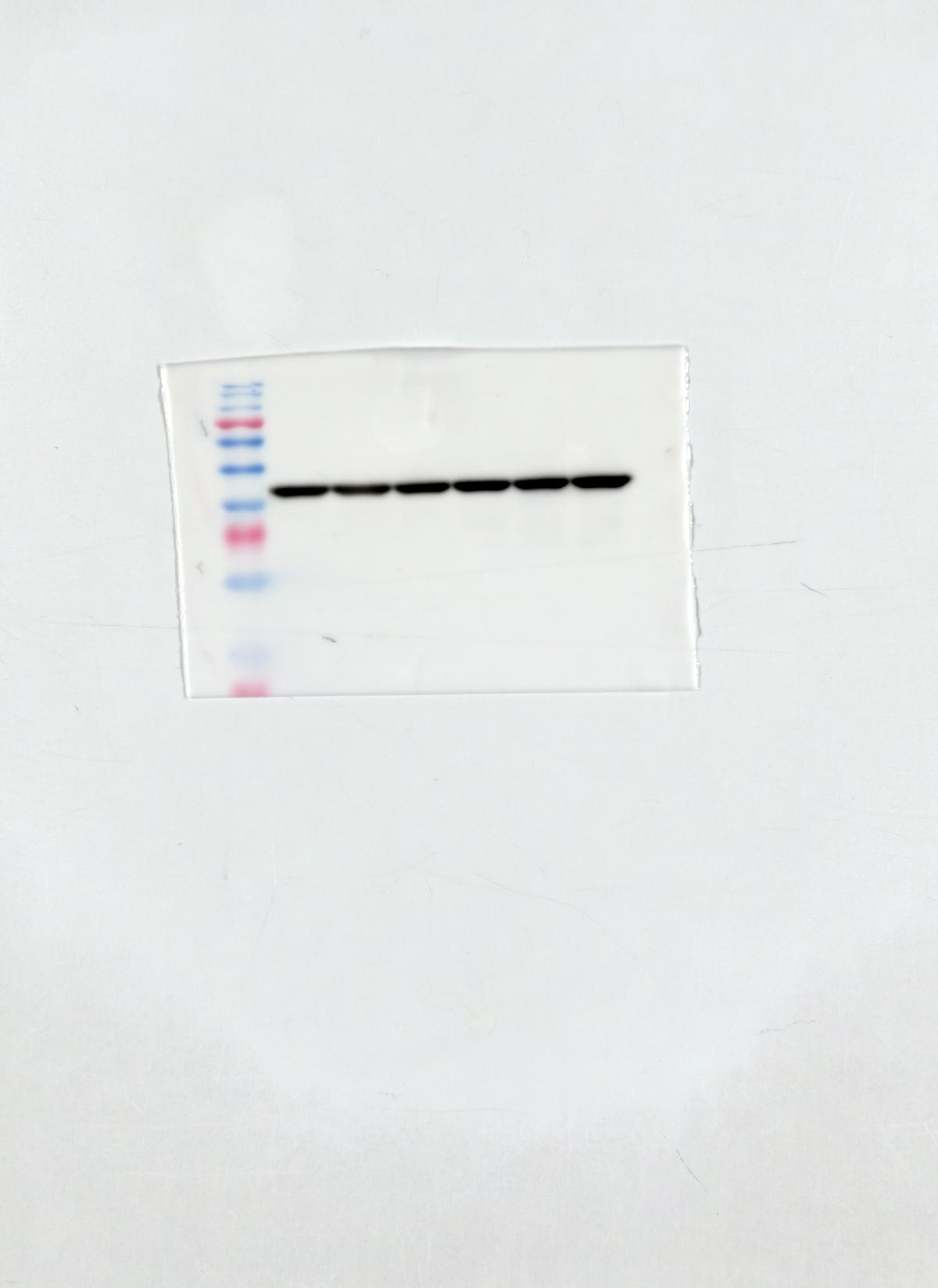

Supplement: Supplementary file 1 [file biomolecules-15-00538-s001.zip › original image/Fig7A.a┬-actin.jpg]

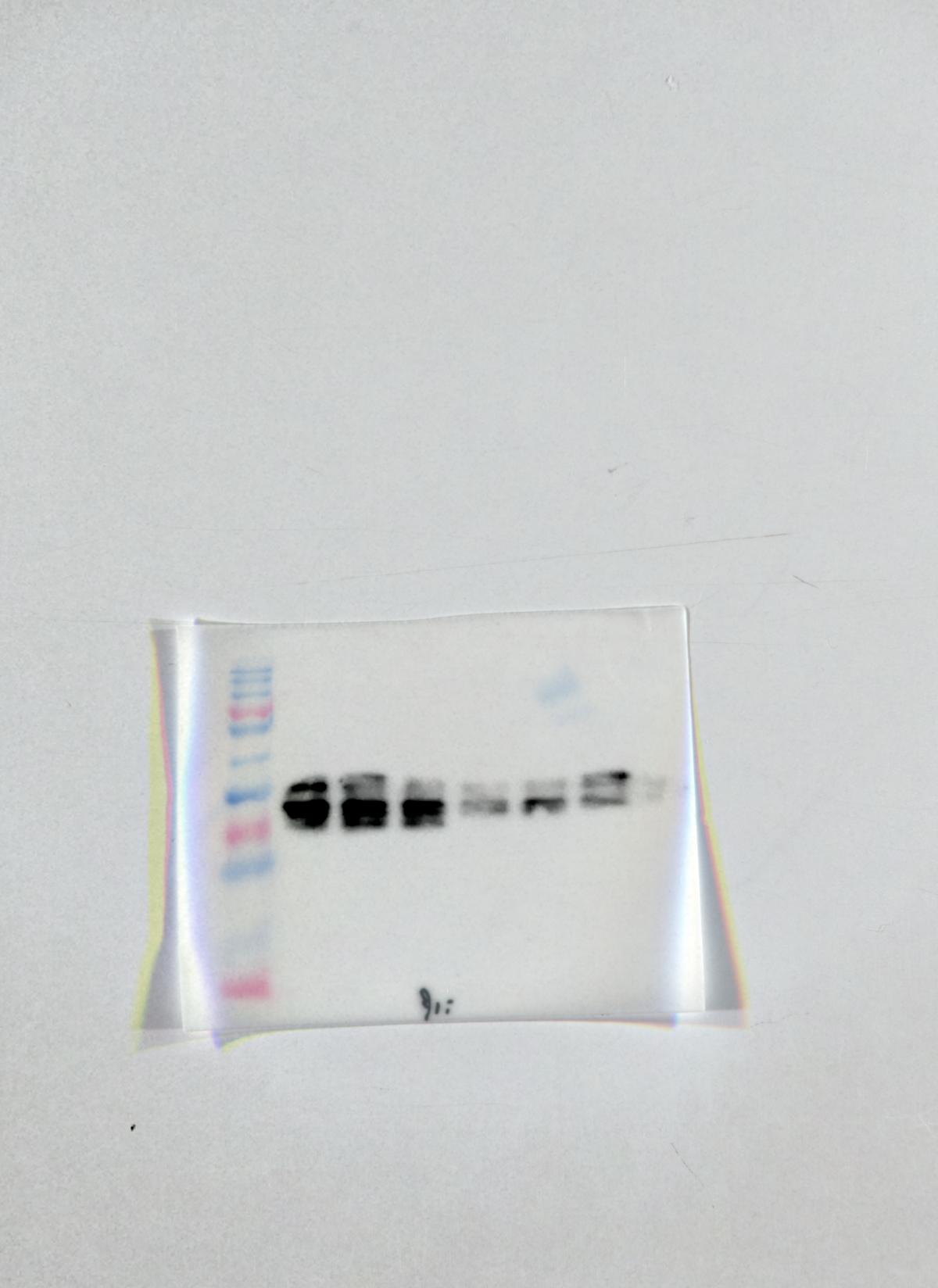

Supplement: Supplementary file 1 [file biomolecules-15-00538-s001.zip › original image/Fig9A.IL-1a┬.jpg]

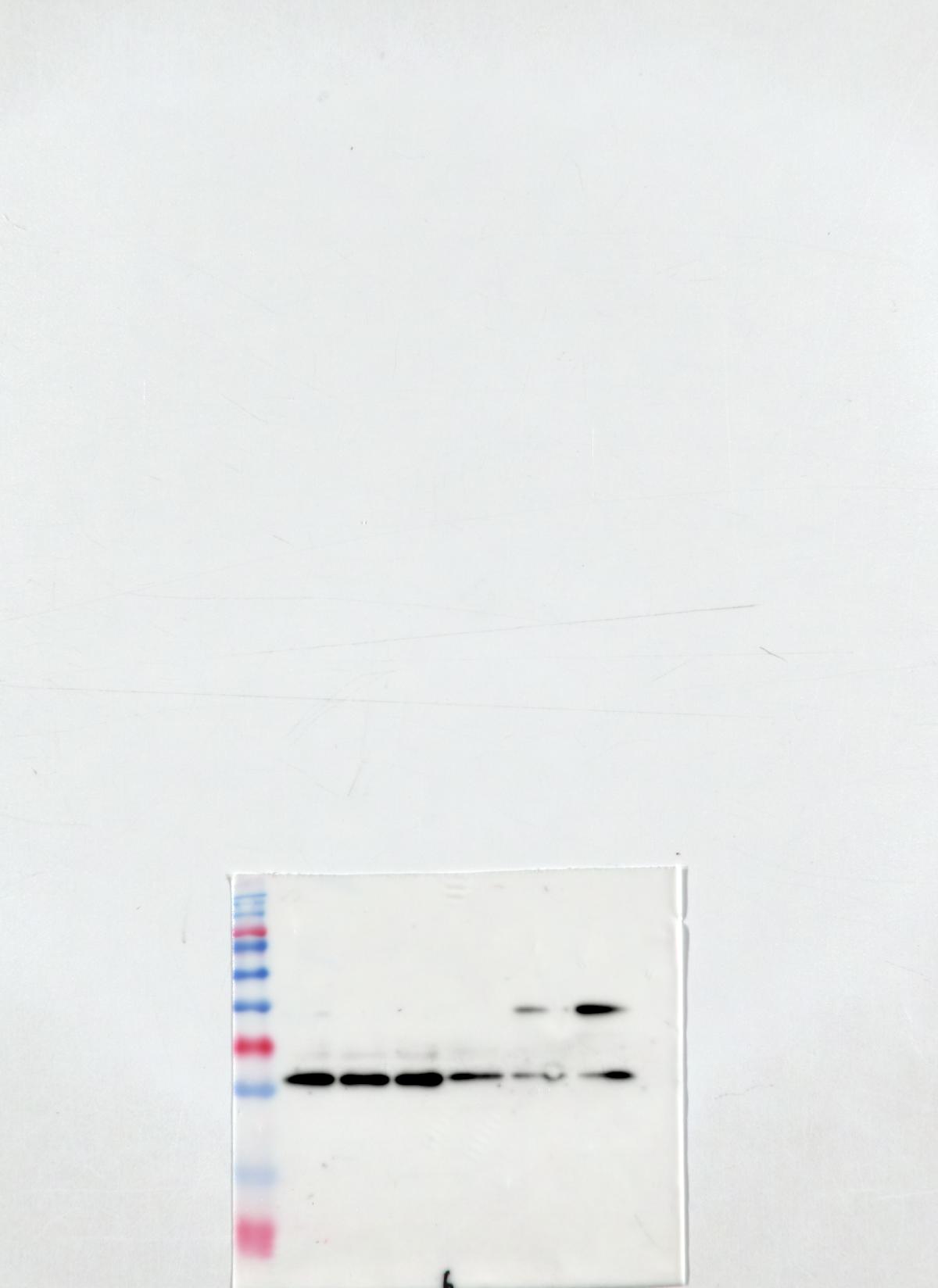

Supplement: Supplementary file 1 [file biomolecules-15-00538-s001.zip › original image/Fig9A.IL-6.jpg]

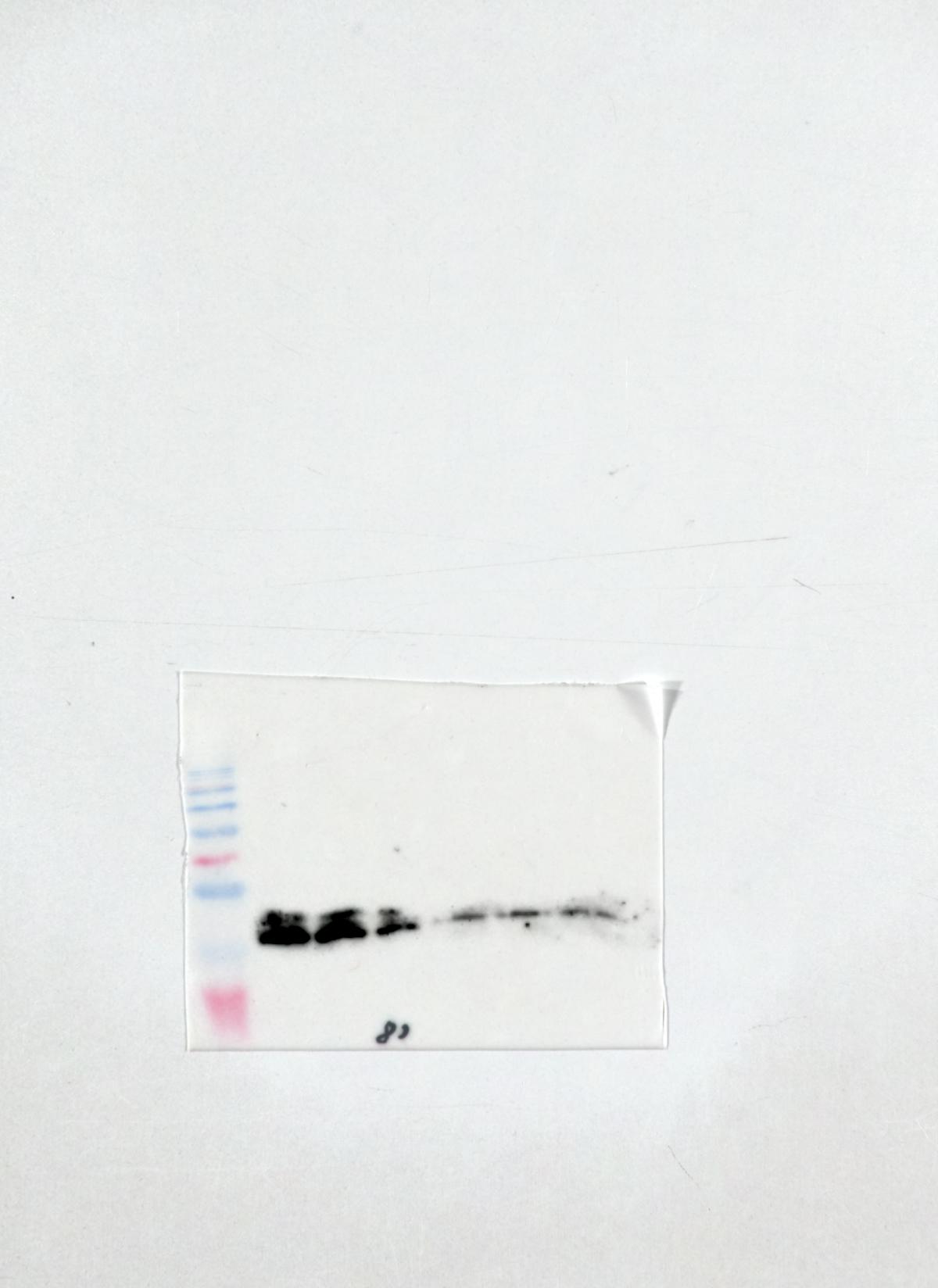

Supplement: Supplementary file 1 [file biomolecules-15-00538-s001.zip › original image/Fig9A.IL-8.jpg]

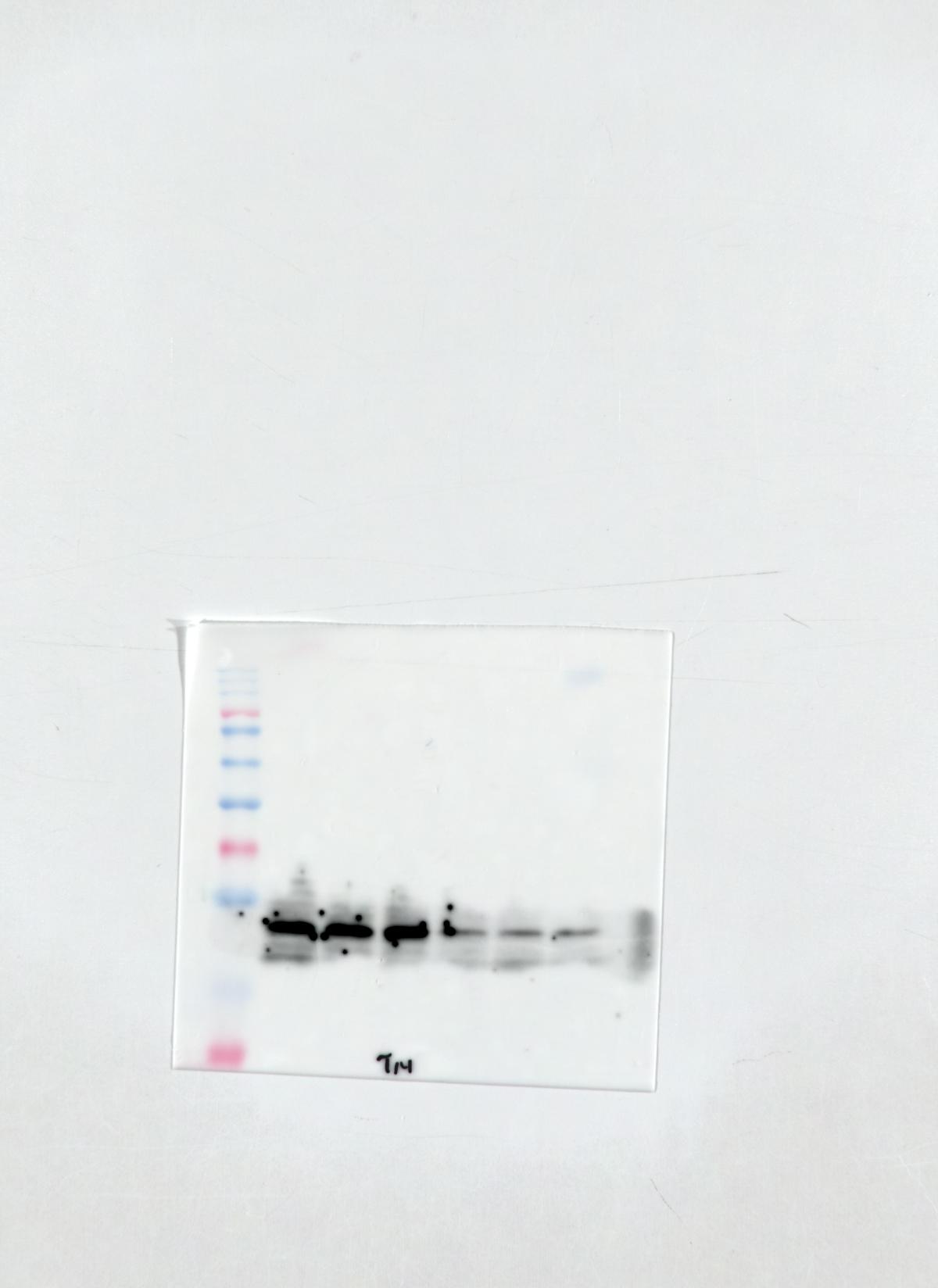

Supplement: Supplementary file 1 [file biomolecules-15-00538-s001.zip › original image/Fig9A.TNF-a┴.jpg]

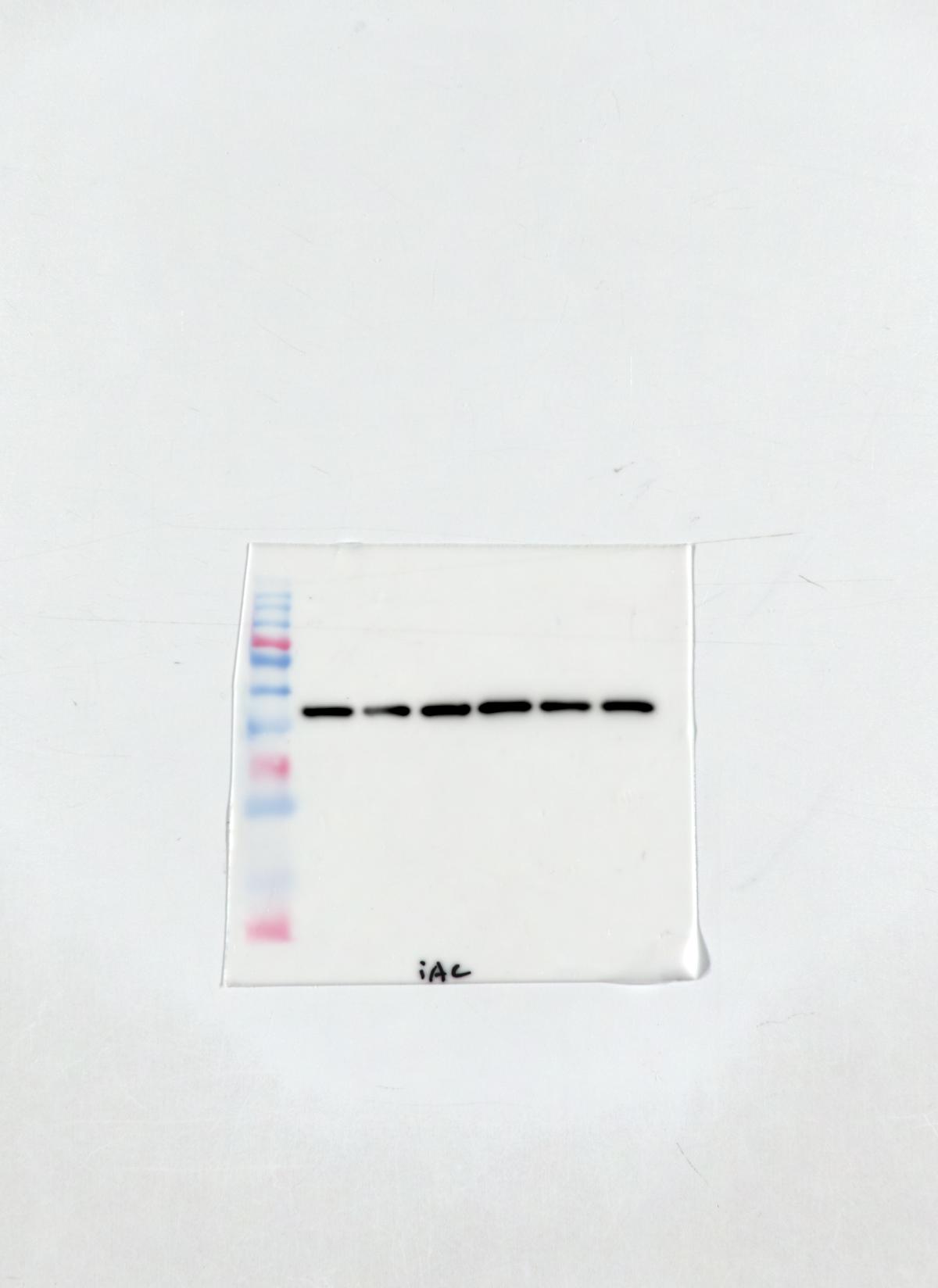

Supplement: Supplementary file 1 [file biomolecules-15-00538-s001.zip › original image/Fig9A.a┬-actin.jpg]
